# Introducing an *rbc*L and a *trn*L reference library to aid in the metabarcoding analysis of foraged plants from two semi-arid eastern South African savanna bioregions

**DOI:** 10.1371/journal.pone.0286144

**Published:** 2023-05-19

**Authors:** Danielle Botha, Mornè du Plessis, Frances Siebert, Sandra Barnard

**Affiliations:** 1 Unit for Environmental Sciences and Management, North-West University, Potchefstroom, South Africa; 2 Core Sequencing Facility, National Institute for Communicable Diseases of the National Health Laboratory Service, Sandringham, Johannesburg, South Africa; Institute for Biological Research, University of Belgrade, SERBIA

## Abstract

Success of a metabarcoding study is determined by the extent of taxonomic coverage and the quality of records available in the DNA barcode reference database used. This study aimed to create an *rbc*L and a *trn*L (UAA) DNA barcode sequence reference database of plant species that are potential herbivore foraging targets and commonly found in semi-arid savannas of eastern South Africa. An area-specific species list of 765 species was compiled according to plant collection records available and areas comparable to an eastern semi-arid South African savanna. Thereafter, *rbc*L and *trn*L sequences of species from this list were mined from GenBank and BOLD sequence databases according to specific quality criteria to ensure accurate taxonomic coverage and resolution. These were supplemented with sequences of 24 species sequenced for this study. A phylogenetic approach, employing Neighbor-Joining, was used to verify the topology of the reference libraries to known angiosperm phylogeny. The taxonomic reliability of these reference libraries was evaluated by testing for the presence of a barcode gap, identifying a data-appropriate identification threshold, and determining the identification accuracy of reference sequences via primary distance-based criteria. The final *rbc*L reference dataset consisted of 1238 sequences representing 318 genera and 562 species. The final *trn*L dataset consisted of 921 sequences representing 270 genera and 461 species. Barcode gaps were found for 76% of the taxa in the *rbc*L barcode reference dataset and 68% of the taxa in the *trn*L barcode reference dataset. The identification success rate, calculated with the *k*-nn criterion was 85.86% for the *rbc*L dataset and 73.72% for the *trn*L dataset. The datasets for *rbc*L and *trn*L combined during this study are not presented as complete DNA reference libraries, but rather as two datasets that should be used in unison to identify plants present in the semi-arid eastern savannas of South Africa.

## 1. Introduction

Dietary analysis is a fundamental part of constructing habitat selection and utilization models as well as assessing the influence of land use type on the plant community and how this, in turn, can influence foraging strategies [1, 2]. Determining and analysing food items in ecosystems will also aid in identifying key environmental resources for the design of reliable conservation and management strategies [3, 4]. To determine the composition of animal diets and how this composition reflects the plant community in question, animal faeces can be examined to identify food items [2, 5], as well as the relative abundance thereof ingested per individual sampled [6].

A non-intrusive approach such as DNA metabarcoding allows for species identification from heterogeneous and degraded environmental samples based on the amplification and subsequent analysis of DNA barcodes [7, 8], which are short gene sequences from a standardized region of the genome that displays low intraspecies and high interspecies variability [9, 10]. DNA metabarcoding provides an attractive alternative to the traditional methods of large-scale species identifications and it can be applied to microscopic [11], cryptic [12], and digested materials [2, 13]. Along with the choice of a barcode to use for the identification of plant species from herbivore diets, herbivore dietary analyses require a comprehensive barcode DNA reference database of sequences from potential dietary items.

In the case of herbivore diet analysis, few studies include the combined use of the core plant barcoding regions, *rbc*L and *mat*K [2, 13, 14], as recommended by the CBOL Plant Working Group [15] due to the required ability of the barcoding regions to amplify degraded and low copy number DNA from faecal samples [16, 17]. Although *mat*K may prove useful in single species detection, its application in metabarcoding may be limited since it may lack the conserved sites suitable for PCR primer binding, is notoriously difficult to amplify in some plant groups, and its length (approximately 800 bp) is ill-suited to most metabarcoding studies [18]. However, the r*bc*L and *trn*L (UAA) DNA barcodes have been proposed as two single-locus gene regions that can be used for the analysis of herbivorous- and omnivorous animal diets via DNA metabarcoding [2, 13, 19–21]. These two barcodes were chosen as the core barcoding regions for this study due to their coverage of different savanna plant species. These barcodes are well represented on public reference databases with 307 756 *rbc*L and 337 987 *trn*L plant sequences on the non-redundant (nt) nucleotide database of the National Centre for Biotechnology Information (NCBI) [22] as of the 7th of November 2022. Advantageous characteristics of *rbc*L include the universality of its primers [16, 23], the conservative coding nature of the region that can be used to reconstruct deep evolutionary relationships, high recoverability, and specimen identification up to family level [5, 19, 24]. In turn, *trn*L is a chloroplastic gene that yields small DNA fragments with the whole intron (254–767 bp) being highly conserved among land plants [19, 25]. It has shown great success in dietary studies due to its ability to resolve taxonomy up to the species level from degraded samples [5, 19, 23, 24].

Except for the choice of the barcoding marker to be used, the efficiency and usefulness of metabarcoding as a tool are underpinned by the extent of taxonomic coverage and the quality of records available in the DNA barcode sequence reference database to which the query sequences will be compared with and identified [26, 27]. However, there is a lack of plant taxonomic sequence information for some ecological regions on global DNA sequence databases [28]. According to Gill *et al*. [28], as of 2019, DNA sequences available on the Barcode of Life Data (BOLD) database represent only ~9% of the vascular plants in Africa, while Africa compromises ~20% of the earth’s landmass and owns approximately 15% of the global plant diversity [29]. In recent decades, African savannas have become a major source of livestock production at the expense of a once-diverse population of native large mammalian herbivores (LMH) [30], and restoration of these degraded populations has become an important conservation goal [2, 31, 32]. The dietary analysis of the composition of herbivorous diets in savanna ecosystems will highlight key savanna plant species that will have significant implications for field management and conservation efforts [33–37]. Savannas are usually characterised by the coexistence of two different plant species, trees and grasses, competing for the same limited resources. However, there are not many ecological studies on the function of forbs, or non-graminoid herbaceous vascular plants [38, 39] in savannas, even though they contribute more than 70% to herbaceous species richness and play a significant role in the diets of various herbivorous guilds [40]. Forbs have therefore been neglected in vegetation studies and knowledge of their ecological role in savanna ecosystems is limited [40], resulting in their weak sequence representation on global DNA barcode databases.

Except for weak species representation of certain geographical areas, it is well known that public DNA reference databases may contain sequencing errors and unverified taxonomies because of a lack of voucher specimen information and links to metadata. This is especially true for GenBank where the curation of the database is left largely to scientists submitting sequences [17, 41]. Since the DNA reference database determines the accuracy of taxonomic assignments, i.e., the success of a barcode in distinguishing between species [42], it is necessary to use a region-specific reference database of curated barcode sequences. This is achieved by creating a subset of the data available on public DNA databases that are manually curated for sequence quality, taxonomic validity, and geographic validity, as well as to test the efficiency of this subset reference database to discriminate between and within species with similarity, distance, and tree-based methods. The curation of geographically valid, i.e., reference databases containing only taxa present at the study site, is advocated by Lamb *et al*. [43], where they proved that the use of such a database will significantly improve the similarity between taxonomic assignments made from NGS (Next Generation Sequencing) data and the actual vegetation from the sampled ecosystem, in contrast to the use of global or region-specific reference databases. This study is the first to create and test barcode-specific reference libraries for plant species occurring in eastern semi-arid savannas of South Africa with special attention to forb species. Since these landscapes are severely impacted by not only large herbivores but also livestock production [44], these reference libraries will enhance future studies focused on determining the floristic diversity of samples from this ecosystem. These findings will have significant implications for field management and conservation practices specific to the eastern semi-arid savannas of South Africa limiting the influence of non-target taxonomies and low-quality sequences from public databases.

This study aims to (i) create comprehensive, study-area-specific *rbc*L and *trn*L (UAA) DNA barcode reference databases of plant species commonly found in a semi-arid South African savanna with special attention to forb species, and (ii) evaluate the efficiency of these barcode reference databases in discriminating between and within species by testing for the presence of barcode gaps, identifying a data appropriate identification threshold as well as determining the identification accuracy of reference sequences via primary distance-based criteria. The fulfilment of these aims will result in two DNA barcode reference databases that can be used confidently in unison to delineate between species found in the faeces of herbivores foraging in a semi-arid eastern South African savanna as well as to use the successful taxonomic identifications to make certain deductions about the plant community to aid in management and conservations strategies.

## 2. Materials and methods

### 2.1. Study area and barcode reference list assembly

**Fig 1:**
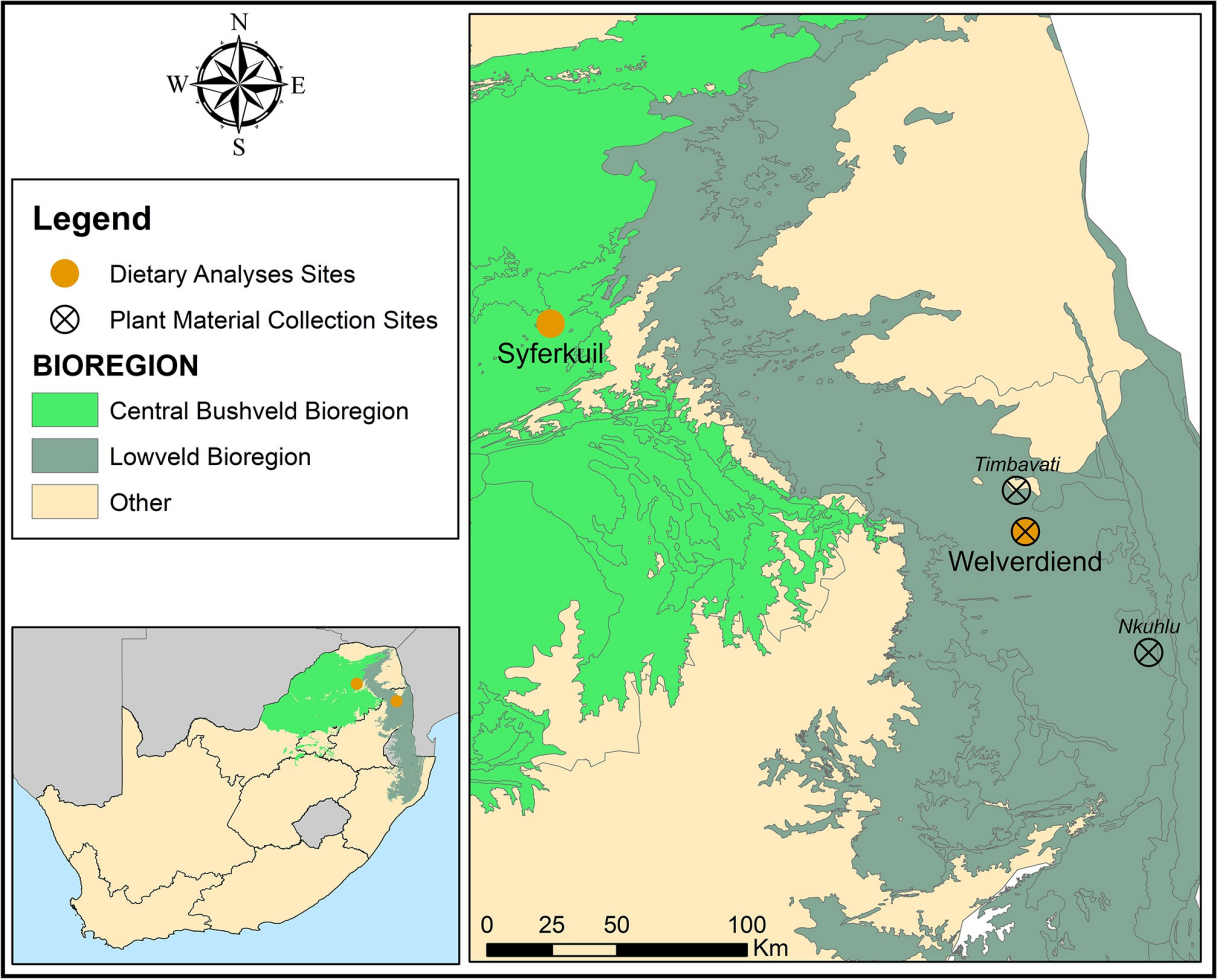
Map showing the sites used for the dietary metabarcoding analysis (Syferkuil and Welverdiend), and collection of plant material (Timbavati, Nkuhlu and Welverdiend) were used for the extraction of POSA species lists. The map was generated in ArcMap 10.8.1 by W. Muller using open-source shapefiles (SANBI (2012) [56], Municipal Demarcation Board [57] and the National Geographic Society [58]).

The compilation of a DNA barcode reference database of species found in semi-arid eastern South African savannas is challenging since scientific publications listing all taxa likely to be encountered in the diets of herbivores foraging in these ecosystems were largely unavailable. The study was therefore limited to the Central Bushveld and Lowveld bioregions in the Limpopo and Mpumalanga provinces in South Africa, as they make out a large proportion of the eastern semi-arid savannas of South Africa receiving a mean annual precipitation of below ~650 mm [45]. Study sites that were later targeted for herbivore faecal samples were located within these two bioregions, namely the rural village of Welverdiend (Limpopo), Syferkuil experimental farm (Limpopo), the Nkuhlu long-term monitoring sites in the Kruger National Park (Mpumalanga) and the Timbavati Private Nature Reserve (Limpopo) (Fig 1). A comprehensive list of all plant species recorded in the areas surrounding the faecal sampling sites was generated through the implementation of a grid search in the Plants of South Africa (POSA) database. Lists of plant species were extracted from a grid of ~10 km radius from the sampling sites to capture the representative flora of each sampling site. In addition to the use of the POSA database, plant species lists were also compiled after plant surveys in these savannas. Unknown species were collected and identified at the A.P. Goossens Herbarium of the North-West University (NWU). Fieldwork and specimen collections conducted were non-invasive and did not involve any capture or disturbance of animals. The only plant material collected was used for barcoding analyses and as voucher specimens. Access to the protected areas complied with regulation 4(1) of the regulations under the National Environmental Management: Protected Areas Act No. 57 of 2003. Permission was granted by the South African National Parks Department, which is responsible for the management and conservation of protected areas and wildlife in South Africa. The North-West University Animal Production Sciences Research Ethics Committee approved the study as zero-risk and granted written permission for its completion. The ethics number is NWU-00177-18-A5. This species list was further optimized by including species from other studies on African savannas [2, 28], based on local expert knowledge [e.g., 40, 46–53]. The species listed by these various sources formed the comprehensive species list used to represent eastern semi-arid South African savannas (S1 Table).

This species list (S1 Table) was then used to mine the species for which *rbc*L and/or *trn*L (UAA) DNA records were available on public databases. These sequences were mined according to specific quality criteria to ensure accurate taxonomic coverage and resolution. These criteria included sourcing a maximum of three individuals per species per marker, not including sequences from the same herbarium, and giving preference to sequences with voucher specimens and sequences with the least ambiguous bases. Accession numbers of species with barcodes that match the quality criteria and have been sequenced with the *rbc*La primers, *rbc*La-F: ATGTCACCACAAACAGAGACTAAAGC [54] and *rbc*La-R: GTAAAATCAAGTCCACCRCG [55] as well as with the *trn*L primers [25], *trn*L-c: CGAAATCGGTAGACGCTACG and *trn*L-d: GGGGATAGAGGGACTTGAAC, can be found in S2 Table. The public databases used to source sequences in this study were the GenBank nucleotide database (www.ncbi.nlm.nih.gov) and the BOLD System public database (www.boldsystems.org). Twenty-four barcode sequences generated during this study with the same primers, from plants found in semi-arid eastern South African Savanna ecosystems, were added to the compiled reference databases (S2 Table). Most of these sequences are known to not occur at all, or not frequently on public DNA sequence databases. Leaf material and voucher specimens of woody and herbaceous plants were collected from Welverdiend, and neighbouring landscapes (Fig 1). Botanists at the NWU examined the voucher specimens to confirm their taxonomy and they were entered into the local Goossens Herbarium of the NWU. These barcode sequences were added to the GenBank nucleotide database. GenBank accession numbers for the *rbc*L barcode range from MZ461574.1 to MZ461594.1 and the *trn*L barcode from MZ461547.1 to MZ461570.1. (S3 Table).

### 2.2. DNA isolation and sequencing

A pipeline for sample collection, DNA isolation, and sequencing of *rbc*L and *trn*L barcodes was established in 2015. Leaf material from 24 plant species for which barcode sequences were not available on any of the public databases was collected between 26 March and 6 April 2015. Approximately 150 mg of fresh leaf material was homogenized in liquid nitrogen after which genomic DNA was extracted from each sample, using the Qiagen DNeasy Plant Extraction kit according to the manufacturer’s protocol. The *rbc*L and *trn*Lc-d barcoding regions were amplified in a 25 μL reaction mix containing 50ng DNA template, 1X KAPA Taq ReadyMix, 1μM of each primer (listed above) and ddH_2_O. Thermocycling was carried out using C1000 Thermal Cycler (BioRad, US). The PCR (Polymerase Chain Reaction) conditions were as follows: the initial denaturation step (94° for 3 min) was followed by 30 cycles of 30 s at 94°C, 30 s at 50°C for the *rbc*L primers and 30 S at 55°C for the *trn*L primers respectively, 30 s at 72°C, followed by a final elongation step of 10 min at 72°C. PCR products were visualized on a 1.5% agarose gel. The amplicons were subsequently sequenced by the Central Analytical Facilities of the University of Stellenbosch, South Africa. Sequencing reactions were performed with the same primers as those used for PCR using the BigDye Terminator V1.3 cycle sequencing kit (Applied Biosystems, USA). This was followed by fluorescence-based DNA analysis using capillary electrophoresis technology of the Applied Biosystems 3500 Genetic Analyser. Sequences were analysed and trimmed using Sequencing Analysis V5.3.1 (Applied Biosystems).

### 2.3. Phylogeny

The candidate barcode reference sequences for both markers to be used for phylogenetic analysis were respectively aligned using Geneious (v. 2021.2.2) [59] via MUltiple Sequence Comparison by Log-Expectation (MUSCLE) [60], with a maximum number of eight iterations. The sequences for the *trn*L marker were not split into families, as they will be for downstream analysis, due to the requirement of robust representations of the alignment and clusters formed for the subsequent “quality” analysis.

The aligned candidate barcode reference sequences were used to reconstruct Neighbor-Joining (NJ) phylogenetic trees for the *rbc*L and *trn*L dataset, as implemented by Geneious with bootstrap testing of 1000 replicates using the Jukes-Cantor model. Within angiosperms, *Amborella* is reported as the sister to all remaining flowering plants [61, 62], and accordingly, the *rbc*L and *trn*L (UAA) genes of *Amborella trichopoda* were included as an outgroup for the *rbc*L and *trn*L sequences and aligned with the rest of the datasets. The individuals in both the *rbc*L and *trn*L phylogenetic tree were also reviewed manually to validate clusters and sub-clusters that were formed. This evaluation was put in place to improve further analyses recommending the removal of sequences showing an evident deviation from their cluster, be it a cluster of life forms (such as trees, shrubs, grasses, or forbs) or plant families. The sequences that were identified to have problematic placements were then replaced with another individual sequence of the species with equal quality if possible, or it was removed from further analysis.

### 2.4. Assembly of the DNA barcode reference database

DNA sequences for both marker datasets were exported to RStudio [63] and aligned to prepare sequences for downstream analysis. Alignment was again facilitated with MUSCLE, but without the outgroup sequences, and subsequent determination of genetic distance and barcode-gap analysis took place. All sequences for *rbc*L were aligned simultaneously using the *AlignSeqs()* function in the DECIPHER package (v.2.0) [64], and *trn*L sequences were split into families [28] and accordingly aligned with the same function due to the often highly divergent sequences displayed for distantly related species. Distance matrices for both datasets were calculated using the Kimura-2 parameter (K2P) model [65] which is the built-in model of evolution in the APE package’s *dist*.*dna()* function (v.5.0) [66].

### 2.5. Barcode gap analysis

The barcode analysis for each marker dataset used the K2P model to calculate genetic distances for each sequence per marker and was done using the R package SPIDER (v.1.5) [67]. A barcode gap analysis is performed by two approaches, as recommended [68], which will allow for barcode gap analyses of the complete datasets, as well as barcode gaps for individual sequences represented in the respective datasets. The first approach compares the median of inter–and intraspecific distances for each marker dataset and assesses the significance of the differences between genetic distances with a Wilcoxon rank-sum test with the R function *wilcox*.*test()* in the R STATS package. The second approach follows the determination of the presence of a barcode gap following [69], where the difference between the maximum intraspecific distance and the minimum interspecific distance is calculated for each sequence per marker dataset. Both approaches were applied during this study.

### 2.6. Analysis with distance-based criteria

The optimisation of the species identification threshold for each marker dataset is performed to increase identification success [42]. During this study, we used the *threshOpt()* function in the SPIDER package of R (v.1.5) [67]: to systematically subject the marker dataset to a range of threshold values (between 0.1 to 0.2% K2P distance). The optimal identification threshold was then calculated based on the number of true positive, false positive, true negative, and false negative identifications for each threshold in the range. The optimal threshold was identified as the value predicting the lowest cumulative error, i.e., the lowest amount of false-positive and negative identifications according to [67].

The predicted accuracy of taxonomic assignation of the reference sequences was determined using three distance-based analyses via the SPIDER package of R (v.1.5) [67]. These primary distance-based criteria included the nearest neighbour (*k*-nn) criterion [70], Meier’s best close match (BCM) criterion [71] as well as BOLD identification criterion [72]. The functions by which these measures are executed:*nearNeighbor()*, *bestCloseMatch()*, and *threshID()*, respectively, performed an internal taxonomic assignation, where individual sequences were treated as unknown queries and the rest of the marker dataset was treated as the DNA reference database to match corresponding taxonomies within the identified optimal threshold.

## 3. Results

### 3.1. DNA reference dataset: phylogenetic analysis and summary statistics

**Fig 2:**
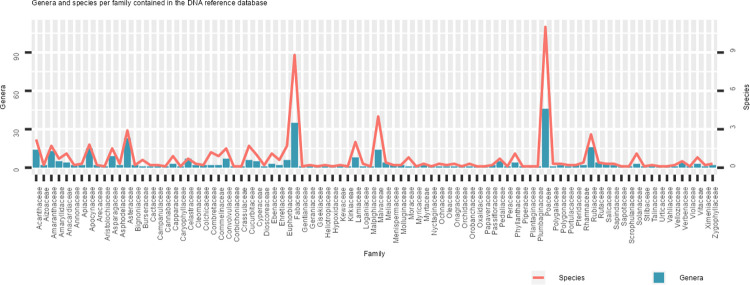
The composition of the final DNA sequence reference database in terms of the number of genera (histogram) and species (line plot) per family.

The final species list (S1 Table) consists of 765 species, belonging to 387 genera, 99 families and representing 3 classes (Magnoliopsida, Liliopsida, and Polypodiopsida) in the phylum Tracheophyta. Of these 765 species, sequences for 118 species could not be found for either the *rbc*L or *trn*L barcode on the public databases GenBank and BOLD. Upon inspection of the NJ trees of *rbc*L and *trn*L respectively, it was advised to replace 265 sequences with other sequences from the same *species* of better quality since they did not group with other members of their family or life form. Sequences for 59 individuals could be replaced, but 206 sequences had to be removed permanently. This led to a loss of taxonomic coverage in some species, now only represented by sequences from < 3 individuals and the complete loss of 57 unique species from the reference datasets. The remaining species and accession numbers, or process identities (process IDs) in the case of BOLD sequences, can be found in the S2 Table which now constitutes the complete list of DNA reference sequences. The composition of the complete reference databases is illustrated in Fig 2, where the family Poaceae is the largest representative with 47 genera and 109 species. The second-largest representative family is the Fabaceae with 35 genera and 87 species. The *rbc*L reference dataset consists of 1238 sequences with a mean length of 756 bp representing 318 genera and 562 species. The *rbc*L barcode is the most conserved with 600 (20.2%) identical sites and a pairwise identity of 86.8%. The *trn*L dataset consists of 921 sequences with a mean length of 699 bp which represent 270 genera and 461 species. There are 89 (1.7%) identical sites and the pairwise identity was 42.1%. Naturally, there is an overlap of species between the datasets as sequences were mined for the same species for both markers: sequences for 345 species of 224 genera are found in both marker datasets. Savana plants are represented by a diverse range of species as also reflected by the sum of the branch lengths of the trees constructed. The sum of the branch length for the *rbc*L NJ tree is 382.343 and 108.23 for the *trn*L NJ tree.

### 3.2. Reference dataset analysis

**Fig 3:**
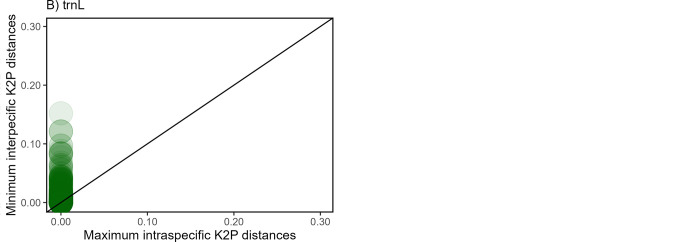
Scatterplots of the Barcode gap s for species present (A) in the *rbc*L dataset and (B) *trn*L dataset. Scatterplots show the relationships between minimum interspecific distance (y-axis) and maximum intraspecific distance (x-axis) where the barcode gap is indicated by points falling above the 1:1 slope. High densities of plots are indicated by the darker colours.

According to the first approach of the barcode gap analysis, by which the presence of a barcode was assessed for both marker datasets by the comparisons of medians for inter-and intraspecific genetic distances, it was found that for the *rbc*L dataset, the interspecific K2P distances ranged from 0,00–0,94 with a median of 0.01. The interspecific distances were statistically larger than the intraspecific K2P distances (Wilcoxon test: P < 2.2e -16) with ranges of 0–1.45 and a median of 0.00. The *trn*L dataset followed the same trend with generally larger interspecific distances (ranges from 0,00–1,01 and a median of 0,01) than intraspecific distances (ranges: 0–1,07; median: 0; Wilcoxon test: p < 2.2e -16). The second approach [71], revealed a positive difference between maximum intra- and minimum interspecific K2P distances for 76% of the *rbc*L sequences and 68% for the *trn*L sequences. These results are reported as scatterplots (Fig 3) where the sequences that showed a gap appear above the 1:1 slope.

The default threshold of species optimisation used by identification algorithms such as BLAST and BOLD is 1%, which may not always be appropriate for all reference datasets. Accordingly, the determined optimal threshold for the *rbc*L dataset was 0.4% and that of the *trn*L dataset was 0.6%, which clearly indicates that the identification accuracy of the datasets would not have been accurately portrayed by the default threshold value of 1%. These optimal threshold values will be used during the simulated taxonomic identification via the three distance-based analyses which will aid in predicting the accuracy of the reference datasets.

The nearest-neighbour (*k*-NN) criterion performed the best among the distance-based measures during the identification simulations (Table 1). The identification success rate, calculated as the number of matches that share the same species index as the query was 85.86% for the complete *rbc*L dataset and 73.72% for the *trn*L dataset. These identification success rates can be increased by excluding singletons, resulting in an identification success rate of 90.78% and therefore an increase of 4.92% for the *rbc*L dataset. Excluding singletons led to an increase of 5.33% resulting in a success rate of 79.05% for the *trn*L dataset. Meier’s [71] best close match (BCM) criterion performed similarly to the *k*-NN identification criterion with an identification success rate of 82.66% correct matches with the optimized threshold value for the exclusion of singletons for the *rbc*L dataset and 64.03% for the *trn*L dataset with matches performed in the optimized threshold with the exclusion of singletons. The BOLD identification criterion failed to show the same level of identification success rates with the inclusion of an optimized identification threshold. The BOLD identification criterion performed the worst among the other measures with the highest discriminating power shown for the *trn*L dataset with the exclusion of singletons, reaching 53.20% with the default threshold value of 1%.

**Table 1:** Discriminatory power of the *rbc*L and *trn*L reference datasets predicted by the application of three distance-based measures: Nearest neighbour, Meier’s best close match, as well as BOLD identification criterion, for both default and optimized thresholds for the inclusion and exclusion of singletons for the respective datasets. All three analyses were performed to identify queries within the respective identification thresholds to genus level.

Dataset	Singleton	Total number of taxa	Threshold value (%)	Nearest Neighbour *		Best Close Match **				BOLD Identification ***			
				False	True	Ambiguous	Correct	Incorrect	No ID	Ambiguous	Correct	Incorrect	No ID
*rbc*L	Included	1238	1	175	1063	104	968	72	94	498	604	42	94
			0,4	NA	NA	102	877	38	221	194	794	29	221
	Excluded	1171	1	108	1063	104	968	48	51	498	604	18	51
			0,4	NA	NA	102	877	29	163	194	7943	20	1631
*trn*L	Included	921	1	242	679	49	550	93	229	183	457	52	229
			0,6	NA	NA	49	508	77	287	129	459	46	287
	Excluded	859	1	180	679	49	550	76	184	183	457	35	184
			0,6	NA	NA	49	508	63	239	129	459	32	239

NA–The identification simulation using the nearest-neighbour criterion does not allow for supplying an alternative identification threshold and only uses the built-in default of 1%.

*Nearest-Neighbour (*k*-NN) criterion result definitions

False–the nearest species index is not the same as the tested individual; True–the nearest species index is the same as the tested individual. Note that the optimal threshold could not be changed from its default in the nearestNeighbour() function.

**Best Close Match (BCM) criterion result definitions:

Ambiguous–Correct and incorrect species are identified as closest matches within the identification threshold; Correct–all close matches within the identification threshold of the query are the same species; Incorrect–all matches within the identification threshold are different species than the query; No ID–no matches could be made within the identification threshold for any individual.

***BOLD identification criterion result definitions:

Ambiguous–Correct and incorrect species are identified as closest matches within the identification threshold; Correct–all close matches within the identification threshold of the query are the same species; Incorrect–all matches within the identification threshold are different species than the query; No ID–no matches could be made within the identification threshold for any individual.

## 4. Discussion

### 4.1. Reference library development

Despite several studies in which DNA barcoding was applied in the analyses of herbivore diets [3, 73, 74], a focus on herbivore diets in African savannas remains limited [2, 26]. Until present, none focused on South African savannas, and there is not currently any robust DNA reference sequence library available for the semi-arid eastern South African Savanna. During this study, we have curated two DNA reference libraries consisting of an *rbc*L and a *trn*L dataset respectively, with special attention to forb species neglected in previous studies and models concerning savanna ecology and definitions. This is the first step towards facilitating metabarcoding studies aimed at South African savanna ecosystems, specifically for the identification of plant materials obtained in herbivorous faeces.

The ideal construction of a reference library would be to sample, identify, and sequence the relevant barcodes of all the species on the species list. However, this would be a costly and laborious research campaign. Mining sequences from global databases is a much more cost-effective option. The method illustrated here can be reproduced for any metabarcoding study aimed at representing the composition of an ecosystem without manually sequencing all specimens. The use of geographically restricted reference databases as proposed in this study may sacrifice the discovery of rare, novel, or invasive species that are not recorded in species lists describing the floristic diversity of the chosen study area in which the metabarcoding study will be conducted. Furthermore, errors made in the early stages of the process or incorrect identifications submitted onto BOLD or GenBank, which condition subsequent choices, may have a devastating impact on the final result. The use of GenBank as a taxonomic resource has been questioned since there exists an absence of preserved voucher specimens, non-justified species identifications, and low-quality data [71, 75]. The same is true for BOLD, although this database is better curated due to higher quality submission standards, it may still contain incorrectly identified taxa and low-quality sequence data because it serves as a workbench for barcode research [71, 72, 76]. However, it has also been shown that the proportion of mislabelled sequences for barcodes on GenBank and BOLD is low and that taxonomic errors are small [75–77]. Being aware of these limitations enables the user to create certain download criteria which result in a database of sequences with known localities, known voucher specimens as well as the inclusion of barcodes with the least ambiguous bases. These databases, as well as any database mined from global reference databases, are not impervious to imperfection, but this method of using a species list and applying download quality criteria will certainly lift the standard above a taxonomic assignation against whole databases such as GenBank and BOLD. Due to the rigorous testing and validation efforts, the barcode databases developed during this study represent a step towards improving reliable identifications for eDNA samples.

The phylogenetic analysis served as a re-identification strategy of sequences included in the *rbc*L and *trn*L reference databases. The exclusion of singletons, as recommended by the identification rates of the various distance-based measures (Table 1) was not considered in this part of the study, as this would have led to a great loss of taxonomic coverage and identifications as seen by a drop of 5.41% and 7.22% in the total taxonomy included in the *rbc*L and *trn*L databases, respectively. Excluding singletons from the reference datasets would lead to a loss of the families of flowering plants, namely: Aristolochiaceae, Dioscoreaceae, Gentianaceae, Kirkiaceae, Passifloraceae, Piperaceae, Plantaginaceae, Urticaceae in the *rbc*L dataset as well as the loss of Hypoxidaceae, Menispermaceae, Nyctaginaceae, Passifloraceae, Piperaceae, Rutaceae, Scrophulariaceae and Stilbaceae in the *trn*L dataset. We, therefore, sacrificed some identification success for the inclusion of singletons, and therefore an accurate representation, in terms of species present, in a South African savanna. The NJ trees revealed that sequences formed cohesive clusters of orders for the *rbc*L dataset. However, an obvious low taxonomic resolution for some orders in the *trn*L dataset was again obvious, such as for the orders Poales, Commelinales and Fabales. Similar results were also shown by Gill *et al*. [28] for the families Poaceae, Fabaceae, and Malvaceae. Low taxonomic resolutions were expected since many genera which are known to occur in South African savannas are under-sampled in global reference databases or are yet to be barcoded with either *rbc*L or *trn*L, leading to a lack of representatives for certain genera or the inclusion of singletons in the reference database. Species represented in the South African savannas are very diverse as can be seen from the sum of the branch lengths of the NJ trees, namely 108.23 for the 922 *trn*L-barcode taxa and 382.34 for the 1239 *rbc*L-barcode taxa.

The lowest level of taxonomic resolution achieved in metabarcoding studies is typically genus-level [73, 78], and the assignation of taxonomy at lower taxonomic levels is often infeasible and inaccurate. Therefore, to ensure that these databases of barcode sequences are robust enough to be used as an identification tool the barcode gap was analysed together with the barcode phylogeny for each dataset. Disparities between inter-and intraspecific distances between and among species are defined as a barcoding gap that, if present at a locus, enables the reliable differentiation of species [42, 67]. A barcode gap was evident in both approaches used and for both datasets. A barcode gap for 76% of the species was obtained for the *rbc*L barcode sequences and 68% of the species for the *trn*L sequences. This implies that reliable species differentiation would not be possible for 24% of the species in the *rbc*L dataset and 32% in the *trn*L dataset. However, the lack of a barcode gap for a considerable proportion of reference sequences has also been reported in other DNA-barcode studies [28, 45, 79]. Gill *et al*. [28] reported a barcode gap for only 73% of the species investigated for the *rbc*L primer and 79% for the *trn*L-F primer in their barcode library of semi-arid East African savanna plants. Mishra *et al*. [77] have shown that even within one genus (*Terminalia* Linn.) the three different barcodes of *mat*K, ITS and *rbc*L contained a barcode gap for <70% of the species. As stated by Gill *et al*. [28] savannas are comprised of a diverse range of species that are prone to the absence of a barcoding gap.

The distance-based measures of *k*-nn, BCM, and BOLD identification criteria infer identification success rates by considering the K2P distance matrix of each dataset to simulate taxonomic identifications of one sequence against the rest of the databases to match taxonomies within the identification threshold [66]. Usually, the distance-based matrices are applied at a species level, but for this metabarcoding study, it was decided to predict the identification success of the datasets based on genus-level identifications (Table 1). The best success rates across all three methods were seen with the exclusion of singletons, in this case, genera represented by only one individual. The analysis of the identification of success rates, with the exclusion of singletons, is also a prerequisite for the alignment that precedes the barcode gap analysis. Singletons are typically a problem in barcoding studies since the identification simulations will treat the singleton as a query, and it will not have a match available in the reference dataset and will result in either “incorrect” or “no identification” [66].

These analyses revealed that the *rbc*L and *trn*L datasets have a predicted identification success rate of 90.78% and 79.05% with the *k*-NN method, respectively. This would imply that a barcode gap analysis is not always an accurate predictor of the species discrimination success of sequences in a reference database, as concluded in certain studies [69, 78, 79]. This is also reflected by the findings in this study as illustrated by the inconsistencies between identification success rate simulations and the evaluation of the barcode gap. However, in this case, the barcode gap analysis can be accepted as a more efficient tool to predict database accuracy since it displays the identification success rate achieved by species-level identifications, whereas the distance-based analysis implemented genus-level taxonomic assignments. In addition, the discrimination success rates demonstrated by both datasets are above (*rbc*L = 76%) or very close (*trn*L = 68%) to the discrimination success rate of 72% as proposed by the CBOL Plant Working Group [15]. This implies that an adequate discrimination success rate would be possible up to the lowest taxonomic level of genus when taking the predicted identification success rates into account. The collection, vouchering and sequencing of more species, especially forb species from South African savannas will aid in more comprehensive public reference databases that can then be used as a sequence mining source to better the resolution of smaller, study-area-specific databases by the addition of new species and the minimization of singletons of other species. Until a standardized plant barcoding region can be agreed upon, reference databases used for metabarcoding studies will not be universally applicable and the choice of barcoding loci is largely left to the researcher based on the targeted taxonomies as well as the nature of the environmental sample. In the meantime, robust DNA barcode reference libraries are essential to moving ecological studies forward.

## 5. Conclusion

During this study, we developed two DNA barcode reference libraries that together are robust enough to identify taxons on the list of species that we curated for some foraged plants including forbs from the semi-arid eastern South African savanna. All the different tests used to validate the use and accuracy of these libraries indicate that they can be used with confidence to assign taxonomies for plants found in the selected bioregions of the eastern savannas of South Africa. The datasets for *rbc*L and *trn*L, to be used for species identification from herbivorous faecal samples, are not presented as a complete DNA reference library, but rather as two databases that should be used in unison to identify species foraged on in the semi-arid eastern South African Savanna. Although the application is study-area-specific, these libraries can also be of use in other similar systems. We envisage that these reference databases will add to other similar research done not only on the local flora but that the methodology applied to compile and validate this resource will advance metabarcoding studies, especially those targeting savannas elsewhere on the African continent.

## Supporting information

S1 Table List of species compiled in this study for the Central Bushveld and Lowveld bioregions in the Limpopo and Mpumalanga provinces in South Africa using the POSA database, related studies and field surveys of the two bioregions. (DOCX)

S2 Table List of species, for which *rbc*L and/or *trn*L barcode sequences were available in public databases, included in the *rbc*L and *trn*L reference datasets with their selected accession numbers (GenBank) and/or process IDs (BOLD). (DOCX)

S3 Table List of species, for which *rbc*L and/or *trn*L barcode sequences were not available in public databases, that were sequenced at the NWU with the *rbc*L and *trn*L barcode, submitted to GenBank, and included in the *rbc*L and *trn*L reference datasets respectively. (DOCX)

S1 File (DOCX)
