# Supplementary material for: Introducing an rbcL and a trnL reference library to aid in the metabarcoding analysis of foraged plants from two semi-arid eastern South African savanna bioregions
Source: PLoS One. 2023 May 19;18(5):e0286144. doi: 10.1371/journal.pone.0286144 (PMC10198553; doi:10.1371/journal.pone.0286144)
Supplement: S1 Table — (DOCX) [file pone.0286144.s001.docx]

Supplementary Table 1: List of species compiled in this study for the Central

Bushveld and Lowveld bioregions in the Limpopo and Mpumalanga provinces in

South Africa using the POSA database, related studies and field surveys of the two

bioregions.

| Phylum | Class | Order | Family | Genus | Species |
| --- | --- | --- | --- | --- | --- |
| Tracheo-phyta | Magnoliopsida | Lamiales | Acanthaceae | *Asystasia* | *Asystasia mysorensis* |
| Tracheo-phyta | Magnoliopsida | Lamiales | Acanthaceae | *Asystasia* | *Asystasia retrocarpa* |
| Tracheo-phyta | Magnoliopsida | Lamiales | Acanthaceae | *Barleria* | *Barleria affinis* |
| Tracheo-phyta | Magnoliopsida | Lamiales | Acanthaceae | *Barleria* | *Barleria crossandriformis* |
| Tracheo-phyta | Magnoliopsida | Lamiales | Acanthaceae | *Barleria* | *Barleria elegans* |
| Tracheo-phyta | Magnoliopsida | Lamiales | Acanthaceae | *Barleria* | *Barleria oxyphylla* |
| Tracheo-phyta | Magnoliopsida | Lamiales | Acanthaceae | *Barleria* | *Barleria prionitis* |
| Tracheo-phyta | Magnoliopsida | Lamiales | Acanthaceae | *Barleria* | *Barleria senensis* |
| Tracheo-phyta | Magnoliopsida | Lamiales | Acanthaceae | *Blepharis* | *Blepharis innocua* |
| Tracheo-phyta | Magnoliopsida | Lamiales | Acanthaceae | *Blepharis* | *Blepharis integrifolia* |
| Tracheo-phyta | Magnoliopsida | Lamiales | Acanthaceae | *Blepharis* | *Blepharis subvolubilis* |
| Tracheo-phyta | Magnoliopsida | Lamiales | Acanthaceae | *Crabbea* | *Crabbea velutina* |
| Tracheo-phyta | Magnoliopsida | Lamiales | Acanthaceae | *Crossandra* | *Crossandra mucronata* |
| Tracheo-phyta | Magnoliopsida | Lamiales | Acanthaceae | *Dyschoriste* | *Dyschoriste fischeri* |
| Tracheo-phyta | Magnoliopsida | Lamiales | Acanthaceae | *Ecbolium* | *Ecbolium glabratum* |
| Tracheo-phyta | Magnoliopsida | Lamiales | Acanthaceae | *Elytraria* | *Elytraria acaulis* |
| Tracheo-phyta | Magnoliopsida | Lamiales | Acanthaceae | *Hygrophila* | *Hygrophila auriculata* |
| Tracheo-phyta | Magnoliopsida | Lamiales | Acanthaceae | *Hypoestes* | *Hypoestes forskaolii* |
| Tracheo-phyta | Magnoliopsida | Lamiales | Acanthaceae | *Justicia* | *Justicia anagalloides* |
| Tracheo-phyta | Magnoliopsida | Lamiales | Acanthaceae | *Justicia* | *Justicia betonica* |
| Tracheo-phyta | Magnoliopsida | Lamiales | Acanthaceae | *Justicia* | *Justicia flava* |
| Tracheo-phyta | Magnoliopsida | Lamiales | Acanthaceae | *Justicia* | *Justicia matammensis* |
| Tracheo-phyta | Magnoliopsida | Lamiales | Acanthaceae | *Justicia* | *Justicia protracta* |
| Tracheo-phyta | Magnoliopsida | Lamiales | Acanthaceae | *Monechma* | *Monechma debile* |
| Tracheo-phyta | Magnoliopsida | Lamiales | Acanthaceae | *Peristrophe* | *Peristrophe cernua* |
| Tracheo-phyta | Magnoliopsida | Lamiales | Acanthaceae | *Rhinacanthus* | *Rhinacanthus xerophilus* |
| Tracheo-phyta | Magnoliopsida | Lamiales | Acanthaceae | *Ruellia* | *Ruellia cordata* |
| Tracheo-phyta | Magnoliopsida | Lamiales | Acanthaceae | *Ruellia* | *Ruellia patula* |
| Tracheo-phyta | Magnoliopsida | Lamiales | Acanthaceae | *Ruellia* | *Ruellia prostrata* |
| Tracheo-phyta | Magnoliopsida | Lamiales | Acanthaceae | *Thunbergia* | *Thunbergia alata* |
| Tracheo-phyta | Magnoliopsida | Caryophyllales | Aizoaceae | *Aizoon* | *Aizoon canariense* |
| Tracheo-phyta | Magnoliopsida | Caryophyllales | Aizoaceae | *Trianthema* | *Trianthema salsoloides* |
| Tracheo-phyta | Magnoliopsida | Caryophyllales | Aizoaceae | *Trianthema* | *Trianthema triquetra* |
| Tracheo-phyta | Magnoliopsida | Caryophyllales | Aizoaceae | *Zaleya* | *Zaleya pentandra* |
| Tracheo-phyta | Magnoliopsida | Caryophyllales | Amaranthaceae | *Achyranthes* | *Achyranthes aspera* |
| Tracheo-phyta | Magnoliopsida | Caryophyllales | Amaranthaceae | *Achyropsis* | *Achyropsis leptostachya* |
| Tracheo-phyta | Magnoliopsida | Caryophyllales | Amaranthaceae | *Aerva* | *Aerva leucura* |
| Tracheo-phyta | Magnoliopsida | Caryophyllales | Amaranthaceae | *Alternanthera* | *Alternanthera pungens* |
| Tracheo-phyta | Magnoliopsida | Caryophyllales | Amaranthaceae | *Alternanthera* | *Alternanthera sessilis* |
| Tracheo-phyta | Magnoliopsida | Caryophyllales | Amaranthaceae | *Amaranthus* | *Amaranthus hybridus* |
| Tracheo-phyta | Magnoliopsida | Caryophyllales | Amaranthaceae | *Amaranthus* | *Amaranthus praetermissus* |
| Tracheo-phyta | Magnoliopsida | Caryophyllales | Amaranthaceae | *Amaranthus* | *Amaranthus thunbergii* |
| Tracheo-phyta | Magnoliopsida | Caryophyllales | Amaranthaceae | *Celosia* | *Celosia trigyna* |
| Tracheo-phyta | Magnoliopsida | Caryophyllales | Amaranthaceae | *Chenopodium* | *Chenopodium album* |
| Tracheo-phyta | Magnoliopsida | Caryophyllales | Amaranthaceae | *Chenopodium* | *Chenopodium ambrosioides* |
| Tracheo-phyta | Magnoliopsida | Caryophyllales | Amaranthaceae | *Cyathula* | *Cyathula lanceolata* |
| Tracheo-phyta | Magnoliopsida | Caryophyllales | Amaranthaceae | *Gomphrena* | *Gomphrena celosioides* |
| Tracheo-phyta | Magnoliopsida | Caryophyllales | Amaranthaceae | *Guilleminea* | *Guilleminea densa* |
| Tracheo-phyta | Magnoliopsida | Caryophyllales | Amaranthaceae | *Hermbstaedtia* | *Hermbstaedtia odorata* |
| Tracheo-phyta | Magnoliopsida | Caryophyllales | Amaranthaceae | *Kyphocarpa* | *Kyphocarpa angustifolia* |
| Tracheo-phyta | Magnoliopsida | Caryophyllales | Amaranthaceae | *Pupalia* | *Pupalia lappacea* |
| Tracheo-phyta | Magnoliopsida | Caryophyllales | Amaranthaceae | *Sericorema* | *Sericorema remotiflora* |
| Tracheo-phyta | Liliopsida | Asparagales | Amaryllidaceae | *Ammocharis* | *Ammocharis coranica* |
| Tracheo-phyta | Liliopsida | Asparagales | Amaryllidaceae | *Boophone* | *Boophone disticha* |
| Tracheo-phyta | Liliopsida | Asparagales | Amaryllidaceae | *Crinum* | *Crinum buphanoides* |
| Tracheo-phyta | Liliopsida | Asparagales | Amaryllidaceae | *Crinum* | *Crinum macowanii* |
| Tracheo-phyta | Liliopsida | Asparagales | Amaryllidaceae | *Crinum* | *Crinum moorei* |
| Tracheo-phyta | Liliopsida | Asparagales | Amaryllidaceae | *Tulbaghia* | *Tulbaghia leucantha* |
| Tracheo-phyta | Magnoliopsida | Sapindales | Anacardiaceae | *Lannea* | *Lannea discolor* |
| Tracheo-phyta | Magnoliopsida | Sapindales | Anacardiaceae | *Lannea* | *Lannea edulis* |
| Tracheo-phyta | Magnoliopsida | Sapindales | Anacardiaceae | *Lannea* | *Lannea schweinfurthii* |
| Tracheo-phyta | Magnoliopsida | Sapindales | Anacardiaceae | *Ozoroa* | *Ozoroa albicans* |
| Tracheo-phyta | Magnoliopsida | Sapindales | Anacardiaceae | *Ozoroa* | *Ozoroa engleri* |
| Tracheo-phyta | Magnoliopsida | Sapindales | Anacardiaceae | *Ozoroa* | *Ozoroa insignis* |
| Tracheo-phyta | Magnoliopsida | Sapindales | Anacardiaceae | *Ozoroa* | *Ozoroa paniculosa* |
| Tracheo-phyta | Magnoliopsida | Sapindales | Anacardiaceae | *Sclerocarya* | *Sclerocarya birrea* |
| Tracheo-phyta | Magnoliopsida | Sapindales | Anacardiaceae | *Searsia* | *Searsia dentata* |
| Tracheo-phyta | Magnoliopsida | Sapindales | Anacardiaceae | *Searsia* | *Searsia gueinzii* |
| Tracheo-phyta | Magnoliopsida | Sapindales | Anacardiaceae | *Searsia* | *Searsia pentheri* |
| Tracheo-phyta | Magnoliopsida | Sapindales | Anacardiaceae | *Searsia* | *Searsia pyroides* |
| Tracheo-phyta | Magnoliopsida | Magnoliales | Annonaceae | *Hexalobus* | *Hexalobus monopetalus* |
| Tracheo-phyta | Magnoliopsida | Magnoliales | Annonaceae | *Monanthotaxis* | *Monanthotaxis caffra* |
| Tracheo-phyta | Magnoliopsida | Apiales | Apiaceae | *Centella* | *Centella asiatica* |
| Tracheo-phyta | Magnoliopsida | Apiales | Apiaceae | *Pappea* | *Pappea capensis* |
| Tracheo-phyta | Magnoliopsida | Gentianales | Apocynaceae | *Adenium* | *Adenium multiflorum* |
| Tracheo-phyta | Magnoliopsida | Gentianales | Apocynaceae | *Adenium* | *Adenium swazicum* |
| Tracheo-phyta | Magnoliopsida | Gentianales | Apocynaceae | *Carissa* | *Carissa bispinosa* |
| Tracheo-phyta | Magnoliopsida | Gentianales | Apocynaceae | *Carissa* | *Carissa edulis* |
| Tracheo-phyta | Magnoliopsida | Gentianales | Apocynaceae | *Catharanthus* | *Catharanthus roseus* |
| Tracheo-phyta | Magnoliopsida | Gentianales | Apocynaceae | *Cryptolepis* | *Cryptolepis oblongifolia* |
| Tracheo-phyta | Magnoliopsida | Gentianales | Apocynaceae | *Cynanchum* | *Cynanchum viminale* |
| Tracheo-phyta | Magnoliopsida | Gentianales | Apocynaceae | *Fockea* | *Fockea angustifolia* |
| Tracheo-phyta | Magnoliopsida | Gentianales | Apocynaceae | *Gomphocarpus* | *Gomphocarpus tomentosus* |
| Tracheo-phyta | Magnoliopsida | Gentianales | Apocynaceae | *Huernia* | *Huernia hystrix* |
| Tracheo-phyta | Magnoliopsida | Gentianales | Apocynaceae | *Pentarrhinum* | *Pentarrhinum insipidum* |
| Tracheo-phyta | Magnoliopsida | Gentianales | Apocynaceae | *Pergularia* | *Pergularia daemia* |
| Tracheo-phyta | Magnoliopsida | Gentianales | Apocynaceae | *Raphionacme* | *Raphionacme elata* |
| Tracheo-phyta | Magnoliopsida | Gentianales | Apocynaceae | *Riocreuxia* | *Riocreuxia torulosa* |
| Tracheo-phyta | Magnoliopsida | Gentianales | Apocynaceae | *Secamone* | *Secamone parvifolia* |
| Tracheo-phyta | Magnoliopsida | Gentianales | Apocynaceae | *Stapelia* | *Stapelia gigantea* |
| Tracheo-phyta | Magnoliopsida | Gentianales | Apocynaceae | *Stomatostemma* | *Stomatostemma monteiroae* |
| Tracheo-phyta | Magnoliopsida | Gentianales | Apocynaceae | *Tacazzea* | *Tacazzea apiculata* |
| Tracheo-phyta | Liliopsida | Alismatales | Araceae | *Stylochaeton* | *Stylochaeton natalensis* |
| Tracheo-phyta | Liliopsida | Arecales | Arecaceae | *Hyphaene* | *Hyphaene coriacea* |
| Tracheo-phyta | Liliopsida | Arecales | Arecaceae | *Phoenix* | *Phoenix reclinata* |
| Tracheo-phyta | Magnoliopsida | Piperales | Aristolochiaceae | *Aristolochia* | *Aristolochia elegans* |
| Tracheo-phyta | Liliopsida | Asparagales | Asparagaceae | *Albuca* | *Albuca setosa* |
| Tracheo-phyta | Liliopsida | Asparagales | Asparagaceae | *Asparagus* | *Asparagus africanus* |
| Tracheo-phyta | Liliopsida | Asparagales | Asparagaceae | *Asparagus* | *Asparagus burchellii* |
| Tracheo-phyta | Liliopsida | Asparagales | Asparagaceae | *Asparagus* | *Asparagus crassicladus* |
| Tracheo-phyta | Liliopsida | Asparagales | Asparagaceae | *Asparagus* | *Asparagus exuvialis* |
| Tracheo-phyta | Liliopsida | Asparagales | Asparagaceae | *Asparagus* | *Asparagus falcatus* |
| Tracheo-phyta | Liliopsida | Asparagales | Asparagaceae | *Asparagus* | *Asparagus plumosus* |
| Tracheo-phyta | Liliopsida | Asparagales | Asparagaceae | *Asparagus* | *Asparagus retrofractus* |
| Tracheo-phyta | Liliopsida | Asparagales | Asparagaceae | *Asparagus* | *Asparagus suaveolens* |
| Tracheo-phyta | Liliopsida | Asparagales | Asparagaceae | *Chlorophytum* | *Chlorophytum galpinii* |
| Tracheo-phyta | Liliopsida | Asparagales | Asparagaceae | *Dipcadi* | *Dipcadi viride* |
| Tracheo-phyta | Liliopsida | Asparagales | Asparagaceae | *Urginea* | *Drimia altissima* |
| Tracheo-phyta | Liliopsida | Asparagales | Asparagaceae | *Drimia* | *Drimia uniflora* |
| Tracheo-phyta | Liliopsida | Asparagales | Asparagaceae | *Eriospermum* | *Eriospermum flagelliforme* |
| Tracheo-phyta | Liliopsida | Asparagales | Asparagaceae | *Ledebouria* | *Ledebouria revoluta* |
| Tracheo-phyta | Liliopsida | Asparagales | Asparagaceae | *Ornithogalum* | *Ornithogalum seineri* |
| Tracheo-phyta | Liliopsida | Asparagales | Asparagaceae | *Sansevieria* | *Sansevieria hyacinthoides* |
| Tracheo-phyta | Liliopsida | Asparagales | Asparagaceae | *Sansevieria* | *Sansevieria pearsonii* |
| Tracheo-phyta | Liliopsida | Asparagales | Asphodelaceae | *Aloe* | *Aloe chabaudii* |
| Tracheo-phyta | Liliopsida | Asparagales | Asphodelaceae | *Aloe* | *Aloe marlothii* |
| Tracheo-phyta | Liliopsida | Asparagales | Asphodelaceae | *Bulbine* | *Bulbine abyssinica* |
| Tracheo-phyta | Magnoliopsida | Asterales | Asteraceae | *Acanthospermum* | *Acanthospermum hispidum* |
| Tracheo-phyta | Magnoliopsida | Asterales | Asteraceae | *Ageratina* | *Ageratina altissima* |
| Tracheo-phyta | Magnoliopsida | Asterales | Asteraceae | *Ageratum* | *Ageratum conyzoides* |
| Tracheo-phyta | Magnoliopsida | Asterales | Asteraceae | *Ambrosia* | *Ambrosia artemisiifolia* |
| Tracheo-phyta | Magnoliopsida | Asterales | Asteraceae | *Aspilia* | *Aspilia mossambicensis* |
| Tracheo-phyta | Magnoliopsida | Asterales | Asteraceae | *Baccharoides* | *Baccharoides adoensis* |
| Tracheo-phyta | Magnoliopsida | Asterales | Asteraceae | *Bidens* | *Bidens bipinnata* |
| Tracheo-phyta | Magnoliopsida | Asterales | Asteraceae | *Bidens* | *Bidens biternata* |
| Tracheo-phyta | Magnoliopsida | Asterales | Asteraceae | *Bidens* | *Bidens pilosa* |
| Tracheo-phyta | Magnoliopsida | Asterales | Asteraceae | *Cirsium* | *Cirsium vulgare* |
| Tracheo-phyta | Magnoliopsida | Asterales | Asteraceae | *Conyza* | *Conyza scabrida* |
| Tracheo-phyta | Magnoliopsida | Asterales | Asteraceae | *Dicoma* | *Dicoma galpinii* |
| Tracheo-phyta | Magnoliopsida | Asterales | Asteraceae | *Emilia* | *Emilia transvaalensis* |
| Tracheo-phyta | Magnoliopsida | Asterales | Asteraceae | *Felicia* | *Felicia bechuanica* |
| Tracheo-phyta | Magnoliopsida | Asterales | Asteraceae | *Geigeria* | *Geigeria burkei* |
| Tracheo-phyta | Magnoliopsida | Asterales | Asteraceae | *Geigeria* | *Geigeria ornativa* |
| Tracheo-phyta | Magnoliopsida | Asterales | Asteraceae | *Helichrysum* | *Helichrysum argyrosphaerum* |
| Tracheo-phyta | Magnoliopsida | Asterales | Asteraceae | *Helichrysum* | *Helichrysum candolleanum* |
| Tracheo-phyta | Magnoliopsida | Asterales | Asteraceae | *Helichrysum* | *Helichrysum miconiifolium* |
| Tracheo-phyta | Magnoliopsida | Asterales | Asteraceae | *Hirpicium* | *Hirpicium bechuanense* |
| Tracheo-phyta | Magnoliopsida | Asterales | Asteraceae | *Nidorella* | *Nidorella resedifolia* |
| Tracheo-phyta | Magnoliopsida | Asterales | Asteraceae | *Parthenium* | *Parthenium hysterophorus* |
| Tracheo-phyta | Magnoliopsida | Asterales | Asteraceae | *Pegolettia* | *Pegolettia senegalensis* |
| Tracheo-phyta | Magnoliopsida | Asterales | Asteraceae | *Pluchea* | *Pluchea bojeri* |
| Tracheo-phyta | Magnoliopsida | Asterales | Asteraceae | *Pseudoconyza* | *Pseudoconyza viscosa* |
| Tracheo-phyta | Magnoliopsida | Asterales | Asteraceae | *Pseudognaphalium* | *Pseudognaphalium luteoalbum* |
| Tracheo-phyta | Magnoliopsida | Asterales | Asteraceae | *Schkuhria* | *Schkuhria pinnata* |
| Tracheo-phyta | Magnoliopsida | Asterales | Asteraceae | *Sphaeranthus* | *Sphaeranthus incisus* |
| Tracheo-phyta | Magnoliopsida | Asterales | Asteraceae | *Tagetes* | *Tagetes minuta* |
| Tracheo-phyta | Magnoliopsida | Asterales | Asteraceae | *Tridax* | *Tridax procumbens* |
| Tracheo-phyta | Magnoliopsida | Asterales | Asteraceae | *Vernonia* | *Vernonia colorata* |
| Tracheo-phyta | Magnoliopsida | Asterales | Asteraceae | *Vernonia* | *Vernonia fastigiata* |
| Tracheo-phyta | Magnoliopsida | Asterales | Asteraceae | *Vernonia* | *Vernonia glabra* |
| Tracheo-phyta | Magnoliopsida | Asterales | Asteraceae | *Vernonia* | *Vernonia oligocephala* |
| Tracheo-phyta | Magnoliopsida | Asterales | Asteraceae | *Vernonia* | *Vernonia poskeana* |
| Tracheo-phyta | Magnoliopsida | Asterales | Asteraceae | *Xanthium* | *Xanthium strumarium* |
| Tracheo-phyta | Magnoliopsida | Asterales | Asteraceae | *Zinnia* | *Zinnia peruviana* |
| Tracheo-phyta | Magnoliopsida | Lamiales | Bignoniaceae | *Kigelia* | *Kigelia africana* |
| Tracheo-phyta | Magnoliopsida | Lamiales | Bignoniaceae | *Rhigozum* | *Rhigozum zambesiacum* |
| Tracheo-phyta | Magnoliopsida | Sapindales | Burseraceae | *Commiphora* | *Commiphora africana* |
| Tracheo-phyta | Magnoliopsida | Sapindales | Burseraceae | *Commiphora* | *Commiphora angolensis* |
| Tracheo-phyta | Magnoliopsida | Sapindales | Burseraceae | *Commiphora* | *Commiphora edulis* |
| Tracheo-phyta | Magnoliopsida | Sapindales | Burseraceae | *Commiphora* | *Commiphora glandulosa* |
| Tracheo-phyta | Magnoliopsida | Sapindales | Burseraceae | *Commiphora* | *Commiphora mollis* |
| Tracheo-phyta | Magnoliopsida | Sapindales | Burseraceae | *Commiphora* | *Commiphora pyracanthoides* |
| Tracheo-phyta | Magnoliopsida | Caryophyllales | Cactaceae | *Cereus* | *Cereus peruvianus* |
| Tracheo-phyta | Magnoliopsida | Caryophyllales | Cactaceae | *Opuntia* | *Opuntia ficus-indica* |
| Tracheo-phyta | Magnoliopsida | Caryophyllales | Cactaceae | *Opuntia* | *Opuntia stricta* |
| Tracheo-phyta | Magnoliopsida | Asterales | Campanulaceae | *Wahlenbergia* | *Wahlenbergia krebsii* |
| Tracheo-phyta | Magnoliopsida | Asterales | Campanulaceae | *Wahlenbergia* | *Wahlenbergia undulata* |
| Tracheo-phyta | Magnoliopsida | Rosales | Cannabaceae | *Trema* | *Trema orientale* |
| Tracheo-phyta | Magnoliopsida | Brassicales | Capparaceae | *Boscia* | *Boscia albitrunca* |
| Tracheo-phyta | Magnoliopsida | Brassicales | Capparaceae | *Boscia* | *Boscia angustifolia* |
| Tracheo-phyta | Magnoliopsida | Brassicales | Capparaceae | *Boscia* | *Boscia mossambicensis* |
| Tracheo-phyta | Magnoliopsida | Brassicales | Capparaceae | *Capparis* | *Capparis sepiaria* |
| Tracheo-phyta | Magnoliopsida | Brassicales | Capparaceae | *Capparis* | *Capparis tomentosa* |
| Tracheo-phyta | Magnoliopsida | Brassicales | Capparaceae | *Maerua* | *Maerua angolensis* |
| Tracheo-phyta | Magnoliopsida | Brassicales | Capparaceae | *Maerua* | *Maerua juncea* |
| Tracheo-phyta | Magnoliopsida | Brassicales | Capparaceae | *Maerua* | *Maerua parvifolia* |
| Tracheo-phyta | Magnoliopsida | Caryophyllales | Caryophyllaceae | *Pollichia* | *Pollichia campestris* |
| Tracheo-phyta | Magnoliopsida | Celastrales | Celastraceae | *Elaeodendron* | *Elaeodendron transvaalense* |
| Tracheo-phyta | Magnoliopsida | Celastrales | Celastraceae | *Gymnosporia* | *Gymnosporia heterophylla* |
| Tracheo-phyta | Magnoliopsida | Celastrales | Celastraceae | *Gymnosporia* | *Gymnosporia polyacantha* |
| Tracheo-phyta | Magnoliopsida | Celastrales | Celastraceae | *Gymnosporia* | *Gymnosporia senegalensis* |
| Tracheo-phyta | Magnoliopsida | Celastrales | Celastraceae | *Maytenus* | *Maytenus acuminata* |
| Tracheo-phyta | Magnoliopsida | Celastrales | Celastraceae | *Mystroxylon* | *Mystroxylon aethiopica* |
| Tracheo-phyta | Magnoliopsida | Celastrales | Celastraceae | *Mystroxylon* | *Mystroxylon aethiopicum* |
| Tracheo-phyta | Magnoliopsida | Celastrales | Celastraceae | *Hippocratea* | *Pristimera longipetiolata* |
| Tracheo-phyta | Magnoliopsida | Celastrales | Celastraceae | *Robsonodendron* | *Robsonodendron eucleiforme* |
| Tracheo-phyta | Magnoliopsida | Brassicales | Cleomaceae | *Cleome* | *Cleome hirta* |
| Tracheo-phyta | Magnoliopsida | Brassicales | Cleomaceae | *Cleome* | *Cleome macrophylla* |
| Tracheo-phyta | Magnoliopsida | Brassicales | Cleomaceae | *Cleome* | *Cleome monophylla* |
| Tracheo-phyta | Magnoliopsida | Brassicales | Cleomaceae | *Gynandropsis* | *Gynandropsis gynandra* |
| Tracheo-phyta | Liliopsida | Liliales | Colchicaceae | *Camptorrhiza* | *Camptorrhiza strumosa* |
| Tracheo-phyta | Liliopsida | Liliales | Colchicaceae | *Gloriosa* | *Gloriosa superba* |
| Tracheo-phyta | Magnoliopsida | Myrtales | Combretaceae | *Combretum* | *Combretum apiculatum* |
| Tracheo-phyta | Magnoliopsida | Myrtales | Combretaceae | *Combretum* | *Combretum collinum* |
| Tracheo-phyta | Magnoliopsida | Myrtales | Combretaceae | *Combretum* | *Combretum erythrophyllum* |
| Tracheo-phyta | Magnoliopsida | Myrtales | Combretaceae | *Combretum* | *Combretum hereroense* |
| Tracheo-phyta | Magnoliopsida | Myrtales | Combretaceae | *Combretum* | *Combretum imberbe* |
| Tracheo-phyta | Magnoliopsida | Myrtales | Combretaceae | *Combretum* | *Combretum microphyllum* |
| Tracheo-phyta | Magnoliopsida | Myrtales | Combretaceae | *Combretum* | *Combretum molle* |
| Tracheo-phyta | Magnoliopsida | Myrtales | Combretaceae | *Combretum* | *Combretum mossambicense* |
| Tracheo-phyta | Magnoliopsida | Myrtales | Combretaceae | *Combretum* | *Combretum zeyheri* |
| Tracheo-phyta | Magnoliopsida | Myrtales | Combretaceae | *Terminalia* | *Terminalia phanerophlebia* |
| Tracheo-phyta | Magnoliopsida | Myrtales | Combretaceae | *Terminalia* | *Terminalia prunioides* |
| Tracheo-phyta | Magnoliopsida | Myrtales | Combretaceae | *Terminalia* | *Terminalia sericea* |
| Tracheo-phyta | Liliopsida | Commelinales | Commelinaceae | *Commelina* | *Commelina africana* |
| Tracheo-phyta | Liliopsida | Commelinales | Commelinaceae | *Commelina* | *Commelina benghalensis* |
| Tracheo-phyta | Liliopsida | Commelinales | Commelinaceae | *Commelina* | *Commelina diffusa* |
| Tracheo-phyta | Liliopsida | Commelinales | Commelinaceae | *Commelina* | *Commelina eckloniana* |
| Tracheo-phyta | Liliopsida | Commelinales | Commelinaceae | *Commelina* | *Commelina erecta* |
| Tracheo-phyta | Liliopsida | Commelinales | Commelinaceae | *Commelina* | *Commelina forskaolii* |
| Tracheo-phyta | Liliopsida | Commelinales | Commelinaceae | *Commelina* | *Commelina subulata* |
| Tracheo-phyta | Liliopsida | Commelinales | Commelinaceae | *Murdannia* | *Murdannia simplex* |
| Tracheo-phyta | Magnoliopsida | Solanales | Convolvulaceae | *Convolvulus* | *Convolvulus farinosus* |
| Tracheo-phyta | Magnoliopsida | Solanales | Convolvulaceae | *Convolvulus* | *Convolvulus sagittatus* |
| Tracheo-phyta | Magnoliopsida | Solanales | Convolvulaceae | *Evolvulus* | *Evolvulus alsinoides* |
| Tracheo-phyta | Magnoliopsida | Solanales | Convolvulaceae | *Ipomoea* | *Ipomoea cairica* |
| Tracheo-phyta | Magnoliopsida | Solanales | Convolvulaceae | *Ipomoea* | *Ipomoea coptica* |
| Tracheo-phyta | Magnoliopsida | Solanales | Convolvulaceae | *Ipomoea* | *Ipomoea crassipes* |
| Tracheo-phyta | Magnoliopsida | Solanales | Convolvulaceae | *Ipomoea* | *Ipomoea lapathifolia* |
| Tracheo-phyta | Magnoliopsida | Solanales | Convolvulaceae | *Ipomoea* | *Ipomoea obscura* |
| Tracheo-phyta | Magnoliopsida | Solanales | Convolvulaceae | *Ipomoea* | *Ipomoea plebeia* |
| Tracheo-phyta | Magnoliopsida | Solanales | Convolvulaceae | *Ipomoea* | *Ipomoea robertsiana* |
| Tracheo-phyta | Magnoliopsida | Solanales | Convolvulaceae | *Ipomoea* | *Ipomoea sinensis* |
| Tracheo-phyta | Magnoliopsida | Solanales | Convolvulaceae | *Ipomoea* | *Ipomoea transvaalensis* |
| Tracheo-phyta | Magnoliopsida | Solanales | Convolvulaceae | *Jacquemontia* | *Jacquemontia tamnifolia* |
| Tracheo-phyta | Magnoliopsida | Solanales | Convolvulaceae | *Merremia* | *Merremia kentrocaulos* |
| Tracheo-phyta | Magnoliopsida | Solanales | Convolvulaceae | *Merremia* | *Merremia palmata* |
| Tracheo-phyta | Magnoliopsida | Solanales | Convolvulaceae | *Seddera* | *Seddera capensis* |
| Tracheo-phyta | Magnoliopsida | Solanales | Convolvulaceae | *Seddera* | *Seddera suffruticosa* |
| Tracheo-phyta | Magnoliopsida | Solanales | Convolvulaceae | *Seddera* | *Senecio speciosus* |
| Tracheo-phyta | Magnoliopsida | Solanales | Convolvulaceae | *Xenostegia* | *Xenostegia tridentata* |
| Tracheo-phyta | Magnoliopsida | Caryophyllales | Corbichoniaceae | *Corbichonia* | *Corbichonia decumbens* |
| Tracheo-phyta | Magnoliopsida | Saxifragales | Crassulaceae | *Cotyledon* | *Cotyledon barbeyi* |
| Tracheo-phyta | Magnoliopsida | Saxifragales | Crassulaceae | *Kalanchoe* | *Kalanchoe paniculata* |
| Tracheo-phyta | Magnoliopsida | Cucurbitales | Cucurbitaceae | *Coccinia* | *Coccinia adoensis* |
| Tracheo-phyta | Magnoliopsida | Cucurbitales | Cucurbitaceae | *Coccinia* | *Coccinia rehmannii* |
| Tracheo-phyta | Magnoliopsida | Cucurbitales | Cucurbitaceae | *Coccinia* | *Coccinia sessilifolia* |
| Tracheo-phyta | Magnoliopsida | Cucurbitales | Cucurbitaceae | *Cucumis* | *Cucumis africanus* |
| Tracheo-phyta | Magnoliopsida | Cucurbitales | Cucurbitaceae | *Cucumis* | *Cucumis anguria* |
| Tracheo-phyta | Magnoliopsida | Cucurbitales | Cucurbitaceae | *Cucumis* | *Cucumis hirsutus* |
| Tracheo-phyta | Magnoliopsida | Cucurbitales | Cucurbitaceae | *Cucumis* | *Cucumis metuliferus* |
| Tracheo-phyta | Magnoliopsida | Cucurbitales | Cucurbitaceae | *Cucumis* | *Cucumis zeyheri* |
| Tracheo-phyta | Magnoliopsida | Cucurbitales | Cucurbitaceae | *Kedrostis* | *Kedrostis africana* |
| Tracheo-phyta | Magnoliopsida | Cucurbitales | Cucurbitaceae | *Kedrostis* | *Kedrostis foetidissima* |
| Tracheo-phyta | Magnoliopsida | Cucurbitales | Cucurbitaceae | *Kedrostis* | *Kedrostis leloja* |
| Tracheo-phyta | Magnoliopsida | Cucurbitales | Cucurbitaceae | *Lagenaria* | *Lagenaria sphaerica* |
| Tracheo-phyta | Magnoliopsida | Cucurbitales | Cucurbitaceae | *Momordica* | *Momordica balsamina* |
| Tracheo-phyta | Magnoliopsida | Cucurbitales | Cucurbitaceae | *Momordica* | *Momordica boivinii* |
| Tracheo-phyta | Magnoliopsida | Cucurbitales | Cucurbitaceae | *Momordica* | *Momordica charantia* |
| Tracheo-phyta | Magnoliopsida | Cucurbitales | Cucurbitaceae | *Momordica* | *Momordica foetida* |
| Tracheo-phyta | Magnoliopsida | Cucurbitales | Cucurbitaceae | *Zehneria* | *Zehneria scabra* |
| Tracheo-phyta | Liliopsida | Poales | Cyperaceae | *Bulbostylis* | *Bulbostylis burchellii* |
| Tracheo-phyta | Liliopsida | Poales | Cyperaceae | *Bulbostylis* | *Bulbostylis hispidula* |
| Tracheo-phyta | Liliopsida | Poales | Cyperaceae | *Cyperus* | *Cyperus castaneus* |
| Tracheo-phyta | Liliopsida | Poales | Cyperaceae | *Cyperus* | *Cyperus compressus* |
| Tracheo-phyta | Liliopsida | Poales | Cyperaceae | *Cyperus* | *Cyperus cyperoides* |
| Tracheo-phyta | Liliopsida | Poales | Cyperaceae | *Cyperus* | *Cyperus esculentus* |
| Tracheo-phyta | Liliopsida | Poales | Cyperaceae | *Cyperus* | *Cyperus indecorus* |
| Tracheo-phyta | Liliopsida | Poales | Cyperaceae | *Cyperus* | *Cyperus obtusiflorus* |
| Tracheo-phyta | Liliopsida | Poales | Cyperaceae | *Cyperus* | *Cyperus pseudovestitus* |
| Tracheo-phyta | Liliopsida | Poales | Cyperaceae | *Cyperus* | *Cyperus rotundus* |
| Tracheo-phyta | Liliopsida | Poales | Cyperaceae | *Cyperus* | *Cyperus rubicundus* |
| Tracheo-phyta | Liliopsida | Poales | Cyperaceae | *Cyperus* | *Cyperus rupestris* |
| Tracheo-phyta | Liliopsida | Poales | Cyperaceae | *Cyperus* | *Cyperus schinzii* |
| Tracheo-phyta | Liliopsida | Poales | Cyperaceae | *Cyperus* | *Cyperus sexangularis* |
| Tracheo-phyta | Liliopsida | Poales | Cyperaceae | *Fimbristylis* | *Fimbristylis complanata* |
| Tracheo-phyta | Liliopsida | Poales | Cyperaceae | *Rhynchospora* | *Rhynchospora brownii* |
| Tracheo-phyta | Liliopsida | Poales | Cyperaceae | *Schoenoplectus* | *Schoenoplectus corymbosus* |
| Tracheo-phyta | Liliopsida | Dioscoreales | Dioscoreaceae | *Dioscorea* | *Dioscorea cotinifolia* |
| Tracheo-phyta | Magnoliopsida | Ericales | Ebenaceae | *Diospyros* | *Diospyros mespiliformis* |
| Tracheo-phyta | Magnoliopsida | Ericales | Ebenaceae | *Euclea* | *Euclea crispa* |
| Tracheo-phyta | Magnoliopsida | Ericales | Ebenaceae | *Euclea* | *Euclea divinorum* |
| Tracheo-phyta | Magnoliopsida | Ericales | Ebenaceae | *Euclea* | *Euclea natalensis* |
| Tracheo-phyta | Magnoliopsida | Ericales | Ebenaceae | *Euclea* | *Euclea schimperi* |
| Tracheo-phyta | Magnoliopsida | Ericales | Ebenaceae | *Euclea* | *Euclea undulata* |
| Tracheo-phyta | Magnoliopsida | Ericales | Ebenaceae | *Royena* | *Royena lycioides* |
| Tracheo-phyta | Magnoliopsida | Boraginales | Ehretiaceae | *Cordia* | *Cordia caffra* |
| Tracheo-phyta | Magnoliopsida | Boraginales | Ehretiaceae | *Cordia* | *Cordia grandicalyx* |
| Tracheo-phyta | Magnoliopsida | Boraginales | Ehretiaceae | *Cordia* | *Cordia monoica* |
| Tracheo-phyta | Magnoliopsida | Boraginales | Ehretiaceae | *Ehretia* | *Ehretia amoena* |
| Tracheo-phyta | Magnoliopsida | Boraginales | Ehretiaceae | *Ehretia* | *Ehretia rigida* |
| Tracheo-phyta | Magnoliopsida | Malpighiales | Euphorbiaceae | *Acalypha* | *Acalypha indica* |
| Tracheo-phyta | Magnoliopsida | Malpighiales | Euphorbiaceae | *Acalypha* | *Acalypha villicaulis* |
| Tracheo-phyta | Magnoliopsida | Malpighiales | Euphorbiaceae | *Croton* | *Croton gratissimus* |
| Tracheo-phyta | Magnoliopsida | Malpighiales | Euphorbiaceae | *Croton* | *Croton megalobotrys* |
| Tracheo-phyta | Magnoliopsida | Malpighiales | Euphorbiaceae | *Dalechampia* | *Dalechampia galpinii* |
| Tracheo-phyta | Magnoliopsida | Malpighiales | Euphorbiaceae | *Euphorbia* | *Euphorbia inaequilatera* |
| Tracheo-phyta | Magnoliopsida | Malpighiales | Euphorbiaceae | *Euphorbia* | *Euphorbia cyathophora* |
| Tracheo-phyta | Magnoliopsida | Malpighiales | Euphorbiaceae | *Euphorbia* | *Euphorbia hirta* |
| Tracheo-phyta | Magnoliopsida | Malpighiales | Euphorbiaceae | *Euphorbia* | *Euphorbia indica* |
| Tracheo-phyta | Magnoliopsida | Malpighiales | Euphorbiaceae | *Euphorbia* | *Euphorbia ingens* |
| Tracheo-phyta | Magnoliopsida | Malpighiales | Euphorbiaceae | *Euphorbia* | *Euphorbia neopolycnemoides* |
| Tracheo-phyta | Magnoliopsida | Malpighiales | Euphorbiaceae | *Euphorbia* | *Euphorbia prostrata* |
| Tracheo-phyta | Magnoliopsida | Malpighiales | Euphorbiaceae | *Euphorbia* | *Euphorbia tirucalli* |
| Tracheo-phyta | Magnoliopsida | Malpighiales | Euphorbiaceae | *Jatropha* | *Jatropha spicata* |
| Tracheo-phyta | Magnoliopsida | Malpighiales | Euphorbiaceae | *Jatropha* | *Jatropha zeyheri* |
| Tracheo-phyta | Magnoliopsida | Malpighiales | Euphorbiaceae | *Ricinus* | *Ricinus communis* |
| Tracheo-phyta | Magnoliopsida | Malpighiales | Euphorbiaceae | *Spirostachys* | *Spirostachys africana* |
| Tracheo-phyta | Magnoliopsida | Malpighiales | Euphorbiaceae | *Tragia* | *Tragia dioica* |
| Tracheo-phyta | Magnoliopsida | Malpighiales | Euphorbiaceae | *Tragia* | *Tragia incisifolia* |
| Tracheo-phyta | Magnoliopsida | Fabales | Fabaceae | *Abrus* | *Abrus precatorius* |
| Tracheo-phyta | Magnoliopsida | Fabales | Fabaceae | *Acacia* | *Acacia borleae* |
| Tracheo-phyta | Magnoliopsida | Fabales | Fabaceae | *Acacia* | *Acacia burkei* |
| Tracheo-phyta | Magnoliopsida | Fabales | Fabaceae | *Acacia* | *Acacia caffra* |
| Tracheo-phyta | Magnoliopsida | Fabales | Fabaceae | *Acacia* | *Acacia erubescens* |
| Tracheo-phyta | Magnoliopsida | Fabales | Fabaceae | *Acacia* | *Acacia robusta* |
| Tracheo-phyta | Magnoliopsida | Fabales | Fabaceae | *Acacia* | *Acacia senegal* |
| Tracheo-phyta | Magnoliopsida | Fabales | Fabaceae | *Aeschynomene* | *Aeschynomene indica* |
| Tracheo-phyta | Magnoliopsida | Fabales | Fabaceae | *Albizia* | *Albizia anthelmintica* |
| Tracheo-phyta | Magnoliopsida | Fabales | Fabaceae | *Albizia* | *Albizia forbesii* |
| Tracheo-phyta | Magnoliopsida | Fabales | Fabaceae | *Albizia* | *Albizia harveyi* |
| Tracheo-phyta | Magnoliopsida | Fabales | Fabaceae | *Albizia* | *Albizia petersiana* |
| Tracheo-phyta | Magnoliopsida | Fabales | Fabaceae | *Albizia* | *Albizia versicolor* |
| Tracheo-phyta | Magnoliopsida | Fabales | Fabaceae | *Alysicarpus* | *Alysicarpus glumaceus* |
| Tracheo-phyta | Magnoliopsida | Fabales | Fabaceae | *Alysicarpus* | *Alysicarpus rugosus* |
| Tracheo-phyta | Magnoliopsida | Fabales | Fabaceae | *Bauhinia* | *Bauhinia galpinii* |
| Tracheo-phyta | Magnoliopsida | Fabales | Fabaceae | *Bolusanthus* | *Bolusanthus speciosus* |
| Tracheo-phyta | Magnoliopsida | Fabales | Fabaceae | *Cassia* | *Cassia abbreviata* |
| Tracheo-phyta | Magnoliopsida | Fabales | Fabaceae | *Chamaecrista* | *Chamaecrista absus* |
| Tracheo-phyta | Magnoliopsida | Fabales | Fabaceae | *Chamaecrista* | *Chamaecrista mimosoides* |
| Tracheo-phyta | Magnoliopsida | Fabales | Fabaceae | *Colophospermum* | *Colophospermum mopane* |
| Tracheo-phyta | Magnoliopsida | Fabales | Fabaceae | *Crotalaria* | *Crotalaria laburnifolia* |
| Tracheo-phyta | Magnoliopsida | Fabales | Fabaceae | *Crotalaria* | *Crotalaria monteiroi* |
| Tracheo-phyta | Magnoliopsida | Fabales | Fabaceae | *Crotalaria* | *Crotalaria podocarpa* |
| Tracheo-phyta | Magnoliopsida | Fabales | Fabaceae | *Crotalaria* | *Crotalaria schinzii* |
| Tracheo-phyta | Magnoliopsida | Fabales | Fabaceae | *Crotalaria* | *Crotalaria sphaerocarpa* |
| Tracheo-phyta | Magnoliopsida | Fabales | Fabaceae | *Crotalaria* | *Crotalaria virgulata* |
| Tracheo-phyta | Magnoliopsida | Fabales | Fabaceae | *Dalbergia* | *Dalbergia melanoxylon* |
| Tracheo-phyta | Magnoliopsida | Fabales | Fabaceae | *Dichrostachys* | *Dichrostachys cinerea* |
| Tracheo-phyta | Magnoliopsida | Fabales | Fabaceae | *Dolichos* | *Dolichos junodii* |
| Tracheo-phyta | Magnoliopsida | Fabales | Fabaceae | *Dolichos* | *Dolichos trilobus* |
| Tracheo-phyta | Magnoliopsida | Fabales | Fabaceae | *Dumasia* | *Dumasia villosa* |
| Tracheo-phyta | Magnoliopsida | Fabales | Fabaceae | *Erythrina* | *Erythrina humeana* |
| Tracheo-phyta | Magnoliopsida | Fabales | Fabaceae | *Erythrina* | *Erythrina latissima* |
| Tracheo-phyta | Magnoliopsida | Fabales | Fabaceae | *Indigastrum* | *Indigastrum costatum* |
| Tracheo-phyta | Magnoliopsida | Fabales | Fabaceae | *Indigofera* | *Indigofera arrecta* |
| Tracheo-phyta | Magnoliopsida | Fabales | Fabaceae | *Indigofera* | *Indigofera astragalina* |
| Tracheo-phyta | Magnoliopsida | Fabales | Fabaceae | *Indigofera* | *Indigofera bainesii* |
| Tracheo-phyta | Magnoliopsida | Fabales | Fabaceae | *Indigofera* | *Indigofera filipes* |
| Tracheo-phyta | Magnoliopsida | Fabales | Fabaceae | *Indigofera* | *Indigofera galpinii* |
| Tracheo-phyta | Magnoliopsida | Fabales | Fabaceae | *Indigofera* | *Indigofera heterotricha* |
| Tracheo-phyta | Magnoliopsida | Fabales | Fabaceae | *Indigofera* | *Indigofera lupatana* |
| Tracheo-phyta | Magnoliopsida | Fabales | Fabaceae | *Indigofera* | *Indigofera lydenburgensis* |
| Tracheo-phyta | Magnoliopsida | Fabales | Fabaceae | *Indigofera* | *Indigofera rhytidocarpa* |
| Tracheo-phyta | Magnoliopsida | Fabales | Fabaceae | *Indigofera* | *Indigofera schimperi* |
| Tracheo-phyta | Magnoliopsida | Fabales | Fabaceae | *Indigofera* | *Indigofera spicata* |
| Tracheo-phyta | Magnoliopsida | Fabales | Fabaceae | *Indigofera* | *Indigofera tinctoria* |
| Tracheo-phyta | Magnoliopsida | Fabales | Fabaceae | *Indigofera* | *Indigofera viciodes* |
| Tracheo-phyta | Magnoliopsida | Fabales | Fabaceae | *Leobordea* | *Leobordea stipulosa* |
| Tracheo-phyta | Magnoliopsida | Fabales | Fabaceae | *Macrotyloma* | *Macrotyloma maranguense* |
| Tracheo-phyta | Magnoliopsida | Fabales | Fabaceae | *Macrotyloma* | *Macrotyloma uniflorum* |
| Tracheo-phyta | Magnoliopsida | Fabales | Fabaceae | *Mundulea* | *Mundulea sericea* |
| Tracheo-phyta | Magnoliopsida | Fabales | Fabaceae | *Neorautanenia* | *Neorautanenia amboensis* |
| Tracheo-phyta | Magnoliopsida | Fabales | Fabaceae | *Ormocarpum* | *Ormocarpum trichocarpum* |
| Tracheo-phyta | Magnoliopsida | Fabales | Fabaceae | *Peltophorum* | *Peltophorum africanum* |
| Tracheo-phyta | Magnoliopsida | Fabales | Fabaceae | *Philenoptera* | *Philenoptera violacea* |
| Tracheo-phyta | Magnoliopsida | Fabales | Fabaceae | *Pterocarpus* | *Pterocarpus angolensis* |
| Tracheo-phyta | Magnoliopsida | Fabales | Fabaceae | *Pterocarpus* | *Pterocarpus rotundifolius* |
| Tracheo-phyta | Magnoliopsida | Fabales | Fabaceae | *Rhynchosia* | *Rhynchosia fleckii* |
| Tracheo-phyta | Magnoliopsida | Fabales | Fabaceae | *Rhynchosia* | *Rhynchosia minima* |
| Tracheo-phyta | Magnoliopsida | Fabales | Fabaceae | *Rhynchosia* | *Rhynchosia monophylla* |
| Tracheo-phyta | Magnoliopsida | Fabales | Fabaceae | *Rhynchosia* | *Rhynchosia totta* |
| Tracheo-phyta | Magnoliopsida | Fabales | Fabaceae | *Schotia* | *Schotia brachypetala* |
| Tracheo-phyta | Magnoliopsida | Fabales | Fabaceae | *Schotia* | *Schotia capitata* |
| Tracheo-phyta | Magnoliopsida | Fabales | Fabaceae | *Senegalia* | *Senegalia galpinii* |
| Tracheo-phyta | Magnoliopsida | Fabales | Fabaceae | *Senegalia* | *Senegalia mellifera* |
| Tracheo-phyta | Magnoliopsida | Fabales | Fabaceae | *Senegalia* | *Senegalia nigrescens* |
| Tracheo-phyta | Magnoliopsida | Fabales | Fabaceae | *Senegalia* | *Senegalia schweinfurthii* |
| Tracheo-phyta | Magnoliopsida | Fabales | Fabaceae | *Senegalia* | *Senegalia welwitschii* |
| Tracheo-phyta | Magnoliopsida | Fabales | Fabaceae | *Senna* | *Senna bicapsularis* |
| Tracheo-phyta | Magnoliopsida | Fabales | Fabaceae | *Senna* | *Senna didymobotrya* |
| Tracheo-phyta | Magnoliopsida | Fabales | Fabaceae | *Senna* | *Senna italica* |
| Tracheo-phyta | Magnoliopsida | Fabales | Fabaceae | *Senna* | *Senna obtusifolia* |
| Tracheo-phyta | Magnoliopsida | Fabales | Fabaceae | *Senna* | *Senna occidentalis* |
| Tracheo-phyta | Magnoliopsida | Fabales | Fabaceae | *Senna* | *Senna petersiana* |
| Tracheo-phyta | Magnoliopsida | Fabales | Fabaceae | *Sesbania* | *Sesbania bispinosa* |
| Tracheo-phyta | Magnoliopsida | Fabales | Fabaceae | *Sphenostylis* | *Sphenostylis angustifolia* |
| Tracheo-phyta | Magnoliopsida | Fabales | Fabaceae | *Stylosanthes* | *Stylosanthes fruticosa* |
| Tracheo-phyta | Magnoliopsida | Fabales | Fabaceae | *Tephrosia* | *Tephrosia longipes* |
| Tracheo-phyta | Magnoliopsida | Fabales | Fabaceae | *Tephrosia* | *Tephrosia multijuga* |
| Tracheo-phyta | Magnoliopsida | Fabales | Fabaceae | *Tephrosia* | *Tephrosia polystachya* |
| Tracheo-phyta | Magnoliopsida | Fabales | Fabaceae | *Tephrosia* | *Tephrosia purpurea* |
| Tracheo-phyta | Magnoliopsida | Fabales | Fabaceae | *Tephrosia* | *Tephrosia rhodesica* |
| Tracheo-phyta | Magnoliopsida | Fabales | Fabaceae | *Tephrosia* | *Tephrosia uniflora* |
| Tracheo-phyta | Magnoliopsida | Fabales | Fabaceae | *Teramnus* | *Teramnus labialis* |
| Tracheo-phyta | Magnoliopsida | Fabales | Fabaceae | *Tylosema* | *Tylosema fassoglense* |
| Tracheo-phyta | Magnoliopsida | Fabales | Fabaceae | *Vachellia* | *Vachellia exuvialis* |
| Tracheo-phyta | Magnoliopsida | Fabales | Fabaceae | *Vachellia* | *Vachellia gerrardii* |
| Tracheo-phyta | Magnoliopsida | Fabales | Fabaceae | *Vachellia* | *Vachellia grandicornuta* |
| Tracheo-phyta | Magnoliopsida | Fabales | Fabaceae | *Vachellia* | *Vachellia karroo* |
| Tracheo-phyta | Magnoliopsida | Fabales | Fabaceae | *Vachellia* | *Vachellia luederitzii* |
| Tracheo-phyta | Magnoliopsida | Fabales | Fabaceae | *Vachellia* | *Vachellia nilotica* |
| Tracheo-phyta | Magnoliopsida | Fabales | Fabaceae | *Vachellia* | *Vachellia tortilis* |
| Tracheo-phyta | Magnoliopsida | Fabales | Fabaceae | *Vachellia* | *Vachellia xanthophloea* |
| Tracheo-phyta | Magnoliopsida | Fabales | Fabaceae | *Vigna* | *Vigna frutescens* |
| Tracheo-phyta | Magnoliopsida | Fabales | Fabaceae | *Vigna* | *Vigna luteola* |
| Tracheo-phyta | Magnoliopsida | Fabales | Fabaceae | *Vigna* | *Vigna unguiculata* |
| Tracheo-phyta | Magnoliopsida | Fabales | Fabaceae | *Vigna* | *Vigna vexillata* |
| Tracheo-phyta | Magnoliopsida | Fabales | Fabaceae | *Xanthocercis* | *Xanthocercis zambesiaca* |
| Tracheo-phyta | Magnoliopsida | Fabales | Fabaceae | *Zornia* | *Zornia glochidiata* |
| Tracheo-phyta | Magnoliopsida | Gentianales | Gentianaceae | *Enicostema* | *Enicostema axillare* |
| Tracheo-phyta | Magnoliopsida | Geraniales | Geraniaceae | *Monsonia* | *Monsonia angustifolia* |
| Tracheo-phyta | Magnoliopsida | Geraniales | Geraniaceae | *Monsonia* | *Monsonia burkeana* |
| Tracheo-phyta | Magnoliopsida | Geraniales | Geraniaceae | *Monsonia* | *Monsonia emarginata* |
| Tracheo-phyta | Magnoliopsida | Geraniales | Geraniaceae | *Monsonia* | *Monsonia glauca* |
| Tracheo-phyta | Magnoliopsida | Caryophyllales | Gisekiaceae | *Gisekia* | *Gisekia africana* |
| Tracheo-phyta | Magnoliopsida | Boraginales | Heliotropiaceae | *Heliotropium* | *Heliotropium ciliatum* |
| Tracheo-phyta | Magnoliopsida | Boraginales | Heliotropiaceae | *Heliotropium* | *Heliotropium ovalifolium* |
| Tracheo-phyta | Magnoliopsida | Boraginales | Heliotropiaceae | *Heliotropium* | *Heliotropium steudneri* |
| Tracheo-phyta | Magnoliopsida | Boraginales | Heliotropiaceae | *Heliotropium* | *Heliotropium strigosum* |
| Tracheo-phyta | Liliopsida | Asparagales | Hypoxidaceae | *Hypoxis* | *Hypoxis hemerocallidea* |
| Tracheo-phyta | Liliopsida | Asparagales | Iridaceae | *Lapeirousia* | *Lapeirousia sandersonii* |
| Tracheo-phyta | Magnoliopsida | Caryophyllales | Kewaceae | *Kewa* | *Kewa bowkeriana* |
| Tracheo-phyta | Magnoliopsida | Caryophyllales | Kewaceae | *Kewa* | *Kewa salsoloides* |
| Tracheo-phyta | Magnoliopsida | Sapindales | Kirkiaceae | *Kirkia* | *Kirkia wilmsii* |
| Tracheo-phyta | Magnoliopsida | Lamiales | Lamiaceae | *Acrotome* | *Acrotome hispida* |
| Tracheo-phyta | Magnoliopsida | Lamiales | Lamiaceae | *Acrotome* | *Acrotome inflata* |
| Tracheo-phyta | Magnoliopsida | Lamiales | Lamiaceae | *Clerodendrum* | *Clerodendrum glabrum* |
| Tracheo-phyta | Magnoliopsida | Lamiales | Lamiaceae | *Clerodendrum* | *Clerodendrum ternatum* |
| Tracheo-phyta | Magnoliopsida | Lamiales | Lamiaceae | *Endostemon* | *Endostemon tereticaulis* |
| Tracheo-phyta | Magnoliopsida | Lamiales | Lamiaceae | *Leonotis* | *Leonotis nepetifolia* |
| Tracheo-phyta | Magnoliopsida | Lamiales | Lamiaceae | *Leonotis* | *Leonotis ocymifolia* |
| Tracheo-phyta | Magnoliopsida | Lamiales | Lamiaceae | *Leucas* | *Leucas capensis* |
| Tracheo-phyta | Magnoliopsida | Lamiales | Lamiaceae | *Leucas* | *Leucas glabrata* |
| Tracheo-phyta | Magnoliopsida | Lamiales | Lamiaceae | *Leucas* | *Leucas martinicensis* |
| Tracheo-phyta | Magnoliopsida | Lamiales | Lamiaceae | *Leucas* | *Leucas neuflizeana* |
| Tracheo-phyta | Magnoliopsida | Lamiales | Lamiaceae | *Leucas* | *Leucas sexdentata* |
| Tracheo-phyta | Magnoliopsida | Lamiales | Lamiaceae | *Ocimum* | *Ocimum americanum* |
| Tracheo-phyta | Magnoliopsida | Lamiales | Lamiaceae | *Ocimum* | *Ocimum filamentosum* |
| Tracheo-phyta | Magnoliopsida | Lamiales | Lamiaceae | *Ocimum* | *Ocimum gratissimum* |
| Tracheo-phyta | Magnoliopsida | Lamiales | Lamiaceae | *Ocimum* | *Ocimum labiatum* |
| Tracheo-phyta | Magnoliopsida | Lamiales | Lamiaceae | *Plectranthus* | *Plectranthus caninus* |
| Tracheo-phyta | Magnoliopsida | Lamiales | Lamiaceae | *Stachys* | *Stachys hyssopoides* |
| Tracheo-phyta | Magnoliopsida | Lamiales | Lamiaceae | *Syncolostemon* | *Syncolostemon elliottii* |
| Tracheo-phyta | Magnoliopsida | Lamiales | Lamiaceae | *Tetradenia* | *Tetradenia riparia* |
| Tracheo-phyta | Magnoliopsida | Laurales | Lauraceae | *Cassytha* | *Cassytha filiformis* |
| Tracheo-phyta | Magnoliopsida | Caryophyllales | Limeaceae | *Limeum* | *Limeum fenestratum* |
| Tracheo-phyta | Magnoliopsida | Caryophyllales | Limeaceae | *Limeum* | *Limeum viscosum* |
| Tracheo-phyta | Magnoliopsida | Malpighiales | Linaceae | *Linum* | *Linum sulcatum* |
| Tracheo-phyta | Magnoliopsida | Gentianales | Loganiaceae | *Strychnos* | *Strychnos madagascariensis* |
| Tracheo-phyta | Magnoliopsida | Gentianales | Loganiaceae | *Strychnos* | *Strychnos spinosa* |
| Tracheo-phyta | Magnoliopsida | Malpighiales | Malpighiaceae | *Sphedamnocarpus* | *Sphedamnocarpus pruriens* |
| Tracheo-phyta | Magnoliopsida | Malpighiales | Malpighiaceae | *Triaspis* | *Triaspis hypericoides* |
| Tracheo-phyta | Magnoliopsida | Malvales | Malvaceae | *Abutilon* | *Abutilon angulatum* |
| Tracheo-phyta | Magnoliopsida | Malvales | Malvaceae | *Abutilon* | *Abutilon austroafricanum* |
| Tracheo-phyta | Magnoliopsida | Malvales | Malvaceae | *Abutilon* | *Abutilon grandiflorum* |
| Tracheo-phyta | Magnoliopsida | Malvales | Malvaceae | *Abutilon* | *Abutilon mauritianum* |
| Tracheo-phyta | Magnoliopsida | Malvales | Malvaceae | *Abutilon* | *Abutilon ramosum* |
| Tracheo-phyta | Magnoliopsida | Malvales | Malvaceae | *Abutilon* | *Abutilon sonneratianum* |
| Tracheo-phyta | Magnoliopsida | Malvales | Malvaceae | *Cienfuegosia* | *Cienfuegosia hildebrandtii* |
| Tracheo-phyta | Magnoliopsida | Malvales | Malvaceae | *Corchorus* | *Corchorus asplenifolius* |
| Tracheo-phyta | Magnoliopsida | Malvales | Malvaceae | *Corchorus* | *Corchorus confusus* |
| Tracheo-phyta | Magnoliopsida | Malvales | Malvaceae | *Corchorus* | *Corchorus trilocularis* |
| Tracheo-phyta | Magnoliopsida | Malvales | Malvaceae | *Dombeya* | *Dombeya rotundifolia* |
| Tracheo-phyta | Magnoliopsida | Malvales | Malvaceae | *Gossypium* | *Gossypium herbaceum* |
| Tracheo-phyta | Magnoliopsida | Malvales | Malvaceae | *Grewia* | *Grewia bicolor* |
| Tracheo-phyta | Magnoliopsida | Malvales | Malvaceae | *Grewia* | *Grewia flava* |
| Tracheo-phyta | Magnoliopsida | Malvales | Malvaceae | *Grewia* | *Grewia flavescens* |
| Tracheo-phyta | Magnoliopsida | Malvales | Malvaceae | *Grewia* | *Grewia hexamita* |
| Tracheo-phyta | Magnoliopsida | Malvales | Malvaceae | *Grewia* | *Grewia monticola* |
| Tracheo-phyta | Magnoliopsida | Malvales | Malvaceae | *Grewia* | *Grewia retinervis* |
| Tracheo-phyta | Magnoliopsida | Malvales | Malvaceae | *Grewia* | *Grewia villosa* |
| Tracheo-phyta | Magnoliopsida | Malvales | Malvaceae | *Hermannia* | *Hermannia boraginiflora* |
| Tracheo-phyta | Magnoliopsida | Malvales | Malvaceae | *Hermannia* | *Hermannia glanduligera* |
| Tracheo-phyta | Magnoliopsida | Malvales | Malvaceae | *Hermannia* | *Hermannia modesta* |
| Tracheo-phyta | Magnoliopsida | Malvales | Malvaceae | *Hibiscus* | *Hibiscus calyphyllus* |
| Tracheo-phyta | Magnoliopsida | Malvales | Malvaceae | *Hibiscus* | *Hibiscus cannabinus* |
| Tracheo-phyta | Magnoliopsida | Malvales | Malvaceae | *Hibiscus* | *Hibiscus engleri* |
| Tracheo-phyta | Magnoliopsida | Malvales | Malvaceae | *Hibiscus* | *Hibiscus lunariifolius* |
| Tracheo-phyta | Magnoliopsida | Malvales | Malvaceae | *Hibiscus* | *Hibiscus micranthus* |
| Tracheo-phyta | Magnoliopsida | Malvales | Malvaceae | *Hibiscus* | *Hibiscus palmatus* |
| Tracheo-phyta | Magnoliopsida | Malvales | Malvaceae | *Hibiscus* | *Hibiscus physaloides* |
| Tracheo-phyta | Magnoliopsida | Malvales | Malvaceae | *Hibiscus* | *Hibiscus praeteritus* |
| Tracheo-phyta | Magnoliopsida | Malvales | Malvaceae | *Hibiscus* | *Hibiscus pusillus* |
| Tracheo-phyta | Magnoliopsida | Malvales | Malvaceae | *Hibiscus* | *Hibiscus sabiensis* |
| Tracheo-phyta | Magnoliopsida | Malvales | Malvaceae | *Hibiscus* | *Hibiscus sidiformis* |
| Tracheo-phyta | Magnoliopsida | Malvales | Malvaceae | *Hibiscus* | *Hibiscus trionum* |
| Tracheo-phyta | Magnoliopsida | Malvales | Malvaceae | *Hibiscus* | *Hibiscus vitifolius* |
| Tracheo-phyta | Magnoliopsida | Malvales | Malvaceae | *Malvastrum* | *Malvastrum coromandelianum* |
| Tracheo-phyta | Magnoliopsida | Malvales | Malvaceae | *Melhania* | *Melhania acuminata* |
| Tracheo-phyta | Magnoliopsida | Malvales | Malvaceae | *Melhania* | *Melhania didyma* |
| Tracheo-phyta | Magnoliopsida | Malvales | Malvaceae | *Melhania* | *Melhania prostrata* |
| Tracheo-phyta | Magnoliopsida | Malvales | Malvaceae | *Pavonia* | *Pavonia burchellii* |
| Tracheo-phyta | Magnoliopsida | Malvales | Malvaceae | *Pavonia* | *Pavonia columella* |
| Tracheo-phyta | Magnoliopsida | Malvales | Malvaceae | *Sida* | *Sida alba* |
| Tracheo-phyta | Magnoliopsida | Malvales | Malvaceae | *Sida* | *Sida cordifolia* |
| Tracheo-phyta | Magnoliopsida | Malvales | Malvaceae | *Sida* | *Sida ovata* |
| Tracheo-phyta | Magnoliopsida | Malvales | Malvaceae | *Sida* | *Sida rhombifolia* |
| Tracheo-phyta | Magnoliopsida | Malvales | Malvaceae | *Sida* | *Sida spinosa* |
| Tracheo-phyta | Magnoliopsida | Malvales | Malvaceae | *Sterculia* | *Sterculia rogersii* |
| Tracheo-phyta | Magnoliopsida | Malvales | Malvaceae | *Triumfetta* | *Triumfetta annua* |
| Tracheo-phyta | Magnoliopsida | Malvales | Malvaceae | *Triumfetta* | *Triumfetta pentandra* |
| Tracheo-phyta | Magnoliopsida | Malvales | Malvaceae | *Triumfetta* | *Triumfetta rhomboidea* |
| Tracheo-phyta | Magnoliopsida | Malvales | Malvaceae | *Waltheria* | *Waltheria indica* |
| Tracheo-phyta | Magnoliopsida | Sapindales | Meliaceae | *Ekebergia* | *Ekebergia capensis* |
| Tracheo-phyta | Magnoliopsida | Sapindales | Meliaceae | *Melia* | *Melia azedarach* |
| Tracheo-phyta | Magnoliopsida | Sapindales | Meliaceae | *Trichilia* | *Trichilia emetica* |
| Tracheo-phyta | Magnoliopsida | Sapindales | Meliaceae | *Turraea* | *Turraea obtusifolia* |
| Tracheo-phyta | Magnoliopsida | Ranunculales | Menispermaceae | *Cissampelos* | *Cissampelos mucronata* |
| Tracheo-phyta | Magnoliopsida | Ranunculales | Menispermaceae | *Cocculus* | *Cocculus hirsutus* |
| Tracheo-phyta | Magnoliopsida | Caryophyllales | Molluginaceae | *Glinus* | *Glinus oppositifolius* |
| Tracheo-phyta | Magnoliopsida | Caryophyllales | Molluginaceae | *Mollugo* | *Mollugo nudicaulis* |
| Tracheo-phyta | Magnoliopsida | Rosales | Moraceae | *Ficus* | *Ficus abutilifolia* |
| Tracheo-phyta | Magnoliopsida | Rosales | Moraceae | *Ficus* | *Ficus capreifolia* |
| Tracheo-phyta | Magnoliopsida | Rosales | Moraceae | *Ficus* | *Ficus ingens* |
| Tracheo-phyta | Magnoliopsida | Rosales | Moraceae | *Ficus* | *Ficus salicifolia* |
| Tracheo-phyta | Magnoliopsida | Rosales | Moraceae | *Ficus* | *Ficus sur* |
| Tracheo-phyta | Magnoliopsida | Rosales | Moraceae | *Ficus* | *Ficus sycomorus* |
| Tracheo-phyta | Magnoliopsida | Rosales | Moraceae | *Ficus* | *Ficus thonningii* |
| Tracheo-phyta | Magnoliopsida | Fagales | Myricaceae | *Morella* | *Morella serrata* |
| Tracheo-phyta | Magnoliopsida | Myrtales | Myrtaceae | *Heteropyxis* | *Heteropyxis natalensis* |
| Tracheo-phyta | Magnoliopsida | Myrtales | Myrtaceae | *Psidium* | *Psidium guajava* |
| Tracheo-phyta | Magnoliopsida | Myrtales | Myrtaceae | *Syzygium* | *Syzygium guineense* |
| Tracheo-phyta | Magnoliopsida | Caryophyllales | Nyctaginaceae | *Boerhavia* | *Boerhavia diffusa* |
| Tracheo-phyta | Magnoliopsida | Malpighiales | Ochnaceae | *Ochna* | *Ochna inermis* |
| Tracheo-phyta | Magnoliopsida | Malpighiales | Ochnaceae | *Ochna* | *Ochna natalitia* |
| Tracheo-phyta | Magnoliopsida | Lamiales | Oleaceae | *Jasminum* | *Jasminum fluminense* |
| Tracheo-phyta | Magnoliopsida | Lamiales | Oleaceae | *Jasminum* | *Jasminum stenolobum* |
| Tracheo-phyta | Magnoliopsida | Myrtales | Onagraceae | *Ludwigia* | *Ludwigia adscendens* |
| Tracheo-phyta | Magnoliopsida | Myrtales | Onagraceae | *Ludwigia* | *Ludwigia octovalvis* |
| Tracheo-phyta | Magnoliopsida | Myrtales | Onagraceae | *Ludwigia* | *Ludwigia stolonifera* |
| Tracheo-phyta | Liliopsida | Asparagales | Orchidaceae | *Ansellia* | *Ansellia africana* |
| Tracheo-phyta | Magnoliopsida | Lamiales | Orobanchaceae | *Striga* | *Striga bilabiata* |
| Tracheo-phyta | Magnoliopsida | Lamiales | Orobanchaceae | *Striga* | *Striga elegans* |
| Tracheo-phyta | Magnoliopsida | Lamiales | Orobanchaceae | *Striga* | *Striga gesnerioides* |
| Tracheo-phyta | Magnoliopsida | Oxalidales | Oxalidaceae | *Oxalis* | *Oxalis latifolia* |
| Tracheo-phyta | Magnoliopsida | Oxalidales | Oxalidaceae | *Oxalis* | *Oxalis obliquifolia* |
| Tracheo-phyta | Magnoliopsida | Oxalidales | Oxalidaceae | *Oxalis* | *Oxalis smithiana* |
| Tracheo-phyta | Magnoliopsida | Ranunculales | Papaveraceae | *Argemone* | *Argemone mexicana* |
| Tracheo-phyta | Magnoliopsida | Malpighiales | Passifloraceae | *Adenia* | *Adenia digitata* |
| Tracheo-phyta | Magnoliopsida | Malpighiales | Passifloraceae | *Tricliceras* | *Tricliceras glanduliferum* |
| Tracheo-phyta | Magnoliopsida | Malpighiales | Passifloraceae | *Tricliceras* | *Tricliceras laceratum* |
| Tracheo-phyta | Magnoliopsida | Malpighiales | Passifloraceae | *Tricliceras* | *Tricliceras longepedunculatum* |
| Tracheo-phyta | Magnoliopsida | Lamiales | Pedaliaceae | *Ceratotheca* | *Ceratotheca triloba* |
| Tracheo-phyta | Magnoliopsida | Lamiales | Pedaliaceae | *Dicerocaryum* | *Dicerocaryum eriocarpum* |
| Tracheo-phyta | Magnoliopsida | Lamiales | Pedaliaceae | *Dicerocaryum* | *Dicerocaryum senecioides* |
| Tracheo-phyta | Magnoliopsida | Lamiales | Pedaliaceae | *Harpagophytum* | *Harpagophytum zeyheri* |
| Tracheo-phyta | Magnoliopsida | Lamiales | Pedaliaceae | *Holubia* | *Holubia saccata* |
| Tracheo-phyta | Magnoliopsida | Lamiales | Pedaliaceae | *Pterodiscus* | *Pterodiscus luridus* |
| Tracheo-phyta | Magnoliopsida | Lamiales | Pedaliaceae | *Sesamum* | *Sesamum alatum* |
| Tracheo-phyta | Magnoliopsida | Lamiales | Pedaliaceae | *Sesamum* | *Sesamum triphyllum* |
| Tracheo-phyta | Magnoliopsida | Malpighiales | Peraceae | *Clutia* | *Clutia pulchella* |
| Tracheo-phyta | Magnoliopsida | Malpighiales | Phyllanthaceae | *Thecacoris* | *Acalypha glabrata* |
| Tracheo-phyta | Magnoliopsida | Malpighiales | Phyllanthaceae | *Bridelia* | *Bridelia cathartica* |
| Tracheo-phyta | Magnoliopsida | Malpighiales | Phyllanthaceae | *Bridelia* | *Bridelia mollis* |
| Tracheo-phyta | Magnoliopsida | Malpighiales | Phyllanthaceae | *Flueggea* | *Flueggea virosa* |
| Tracheo-phyta | Magnoliopsida | Malpighiales | Phyllanthaceae | *Phyllanthus* | *Phyllanthus asperulatus* |
| Tracheo-phyta | Magnoliopsida | Malpighiales | Phyllanthaceae | *Phyllanthus* | *Phyllanthus incurvus* |
| Tracheo-phyta | Magnoliopsida | Malpighiales | Phyllanthaceae | *Phyllanthus* | *Phyllanthus maderaspatensis* |
| Tracheo-phyta | Magnoliopsida | Malpighiales | Phyllanthaceae | *Phyllanthus* | *Phyllanthus nummulariifolius* |
| Tracheo-phyta | Magnoliopsida | Malpighiales | Phyllanthaceae | *Phyllanthus* | *Phyllanthus parvulus* |
| Tracheo-phyta | Magnoliopsida | Malpighiales | Phyllanthaceae | *Phyllanthus* | *Phyllanthus pentandrus* |
| Tracheo-phyta | Magnoliopsida | Malpighiales | Phyllanthaceae | *Phyllanthus* | *Phyllanthus reticulatus* |
| Tracheo-phyta | Magnoliopsida | Piperales | Piperaceae | *Peperomia* | *Peperomia blanda* |
| Tracheo-phyta | Magnoliopsida | Lamiales | Plantaginaceae | *Bacopa* | *Bacopa floribunda* |
| Tracheo-phyta | Magnoliopsida | Caryophyllales | Plumbaginaceae | *Plumbago* | *Plumbago zeylanica* |
| Tracheo-phyta | Liliopsida | Poales | Poaceae | *Andropogon* | *Andropogon chinensis* |
| Tracheo-phyta | Liliopsida | Poales | Poaceae | *Andropogon* | *Andropogon gayanus* |
| Tracheo-phyta | Liliopsida | Poales | Poaceae | *Aristida* | *Aristida adscensionis* |
| Tracheo-phyta | Liliopsida | Poales | Poaceae | *Aristida* | *Aristida bipartita* |
| Tracheo-phyta | Liliopsida | Poales | Poaceae | *Aristida* | *Aristida canescens* |
| Tracheo-phyta | Liliopsida | Poales | Poaceae | *Aristida* | *Aristida congesta* |
| Tracheo-phyta | Liliopsida | Poales | Poaceae | *Aristida* | *Aristida meridionalis* |
| Tracheo-phyta | Liliopsida | Poales | Poaceae | *Aristida* | *Aristida mollissima* |
| Tracheo-phyta | Liliopsida | Poales | Poaceae | *Aristida* | *Aristida scabrivalvis* |
| Tracheo-phyta | Liliopsida | Poales | Poaceae | *Aristida* | *Aristida stipitata* |
| Tracheo-phyta | Liliopsida | Poales | Poaceae | *Aristida* | *Aristida vestita* |
| Tracheo-phyta | Liliopsida | Poales | Poaceae | *Bothriochloa* | *Bothriochloa bladhii* |
| Tracheo-phyta | Liliopsida | Poales | Poaceae | *Bothriochloa* | *Bothriochloa insculpta* |
| Tracheo-phyta | Liliopsida | Poales | Poaceae | *Bothriochloa* | *Bothriochloa radicans* |
| Tracheo-phyta | Liliopsida | Poales | Poaceae | *Brachiaria* | *Brachiaria deflexa* |
| Tracheo-phyta | Liliopsida | Poales | Poaceae | *Brachiaria* | *Brachiaria eruciformis* |
| Tracheo-phyta | Liliopsida | Poales | Poaceae | *Brachiaria* | *Brachiaria nigropedata* |
| Tracheo-phyta | Liliopsida | Poales | Poaceae | *Brachiaria* | *Brachiaria serrata* |
| Tracheo-phyta | Liliopsida | Poales | Poaceae | *Brachiaria* | *Brachiaria xantholeuca* |
| Tracheo-phyta | Liliopsida | Poales | Poaceae | *Cenchrus* | *Cenchrus ciliaris* |
| Tracheo-phyta | Liliopsida | Poales | Poaceae | *Chloris* | *Chloris gayana* |
| Tracheo-phyta | Liliopsida | Poales | Poaceae | *Chloris* | *Chloris pycnothrix* |
| Tracheo-phyta | Liliopsida | Poales | Poaceae | *Chloris* | *Chloris roxburghiana* |
| Tracheo-phyta | Liliopsida | Poales | Poaceae | *Chloris* | *Chloris virgata* |
| Tracheo-phyta | Liliopsida | Poales | Poaceae | *Cymbopogon* | *Cymbopogon excavatus* |
| Tracheo-phyta | Liliopsida | Poales | Poaceae | *Cymbopogon* | *Cymbopogon plurinodis* |
| Tracheo-phyta | Liliopsida | Poales | Poaceae | *Cynodon* | *Cynodon dactylon* |
| Tracheo-phyta | Liliopsida | Poales | Poaceae | *Dactyloctenium* | *Dactyloctenium aegyptium* |
| Tracheo-phyta | Liliopsida | Poales | Poaceae | *Dactyloctenium* | *Dactyloctenium australe* |
| Tracheo-phyta | Liliopsida | Poales | Poaceae | *Dactyloctenium* | *Dactyloctenium giganteum* |
| Tracheo-phyta | Liliopsida | Poales | Poaceae | *Dichanthium* | *Dichanthium annulatum* |
| Tracheo-phyta | Liliopsida | Poales | Poaceae | *Digitaria* | *Digitaria debilis* |
| Tracheo-phyta | Liliopsida | Poales | Poaceae | *Digitaria* | *Digitaria eriantha* |
| Tracheo-phyta | Liliopsida | Poales | Poaceae | *Digitaria* | *Digitaria ternata* |
| Tracheo-phyta | Liliopsida | Poales | Poaceae | *Digitaria* | *Digitaria velutina* |
| Tracheo-phyta | Liliopsida | Poales | Poaceae | *Diheteropogon* | *Diheteropogon amplectens* |
| Tracheo-phyta | Liliopsida | Poales | Poaceae | *Diplachne* | *Diplachne fusca* |
| Tracheo-phyta | Liliopsida | Poales | Poaceae | *Echinochloa* | *Echinochloa colona* |
| Tracheo-phyta | Liliopsida | Poales | Poaceae | *Elionurus* | *Elionurus muticus* |
| Tracheo-phyta | Liliopsida | Poales | Poaceae | *Enneapogon* | *Enneapogon cenchroides* |
| Tracheo-phyta | Liliopsida | Poales | Poaceae | *Enneapogon* | *Enneapogon scoparius* |
| Tracheo-phyta | Liliopsida | Poales | Poaceae | *Enteropogon* | *Enteropogon macrostachyus* |
| Tracheo-phyta | Liliopsida | Poales | Poaceae | *Eragrostis* | *Eragrostis aspera* |
| Tracheo-phyta | Liliopsida | Poales | Poaceae | *Eragrostis* | *Eragrostis biflora* |
| Tracheo-phyta | Liliopsida | Poales | Poaceae | *Eragrostis* | *Eragrostis capensis* |
| Tracheo-phyta | Liliopsida | Poales | Poaceae | *Eragrostis* | *Eragrostis chloromelas* |
| Tracheo-phyta | Liliopsida | Poales | Poaceae | *Eragrostis* | *Eragrostis cilianensis* |
| Tracheo-phyta | Liliopsida | Poales | Poaceae | *Eragrostis* | *Eragrostis curvula* |
| Tracheo-phyta | Liliopsida | Poales | Poaceae | *Eragrostis* | *Eragrostis cylindriflora* |
| Tracheo-phyta | Liliopsida | Poales | Poaceae | *Eragrostis* | *Eragrostis gummiflua* |
| Tracheo-phyta | Liliopsida | Poales | Poaceae | *Eragrostis* | *Eragrostis heteromera* |
| Tracheo-phyta | Liliopsida | Poales | Poaceae | *Eragrostis* | *Eragrostis inamoena* |
| Tracheo-phyta | Liliopsida | Poales | Poaceae | *Eragrostis* | *Eragrostis lehmanniana* |
| Tracheo-phyta | Liliopsida | Poales | Poaceae | *Eragrostis* | *Eragrostis nindensis* |
| Tracheo-phyta | Liliopsida | Poales | Poaceae | *Eragrostis* | *Eragrostis pallens* |
| Tracheo-phyta | Liliopsida | Poales | Poaceae | *Eragrostis* | *Eragrostis racemosa* |
| Tracheo-phyta | Liliopsida | Poales | Poaceae | *Eragrostis* | *Eragrostis rigidior* |
| Tracheo-phyta | Liliopsida | Poales | Poaceae | *Eragrostis* | *Eragrostis rotifer* |
| Tracheo-phyta | Liliopsida | Poales | Poaceae | *Eragrostis* | *Eragrostis stapfii* |
| Tracheo-phyta | Liliopsida | Poales | Poaceae | *Eragrostis* | *Eragrostis superba* |
| Tracheo-phyta | Liliopsida | Poales | Poaceae | *Eragrostis* | *Eragrostis trichophora* |
| Tracheo-phyta | Liliopsida | Poales | Poaceae | *Eriochloa* | *Eriochloa meyeriana* |
| Tracheo-phyta | Liliopsida | Poales | Poaceae | *Eustachys* | *Eustachys paspaloides* |
| Tracheo-phyta | Liliopsida | Poales | Poaceae | *Fingerhuthia* | *Fingerhuthia africana* |
| Tracheo-phyta | Liliopsida | Poales | Poaceae | *Hemarthria* | *Hemarthria altissima* |
| Tracheo-phyta | Liliopsida | Poales | Poaceae | *Heteropogon* | *Heteropogon contortus* |
| Tracheo-phyta | Liliopsida | Poales | Poaceae | *Hyparrhenia* | *Hyparrhenia filipendula* |
| Tracheo-phyta | Liliopsida | Poales | Poaceae | *Hyperthelia* | *Hyperthelia dissoluta* |
| Tracheo-phyta | Liliopsida | Poales | Poaceae | *Megathyrsus* | *Megathyrsus maximus* |
| Tracheo-phyta | Liliopsida | Poales | Poaceae | *Melinis* | *Melinis nerviglumis* |
| Tracheo-phyta | Liliopsida | Poales | Poaceae | *Melinis* | *Melinis repens* |
| Tracheo-phyta | Liliopsida | Poales | Poaceae | *Microchloa* | *Microchloa caffra* |
| Tracheo-phyta | Liliopsida | Poales | Poaceae | *Oropetium* | *Oropetium capense* |
| Tracheo-phyta | Liliopsida | Poales | Poaceae | *Panicum* | *Panicum coloratum* |
| Tracheo-phyta | Liliopsida | Poales | Poaceae | *Panicum* | *Panicum deustum* |
| Tracheo-phyta | Liliopsida | Poales | Poaceae | *Panicum* | *Panicum infestum* |
| Tracheo-phyta | Liliopsida | Poales | Poaceae | *Panicum* | *Panicum maximum* |
| Tracheo-phyta | Liliopsida | Poales | Poaceae | *Panicum* | *Panicum natalense* |
| Tracheo-phyta | Liliopsida | Poales | Poaceae | *Paspalum* | *Paspalum distichum* |
| Tracheo-phyta | Liliopsida | Poales | Poaceae | *Paspalum* | *Paspalum scrobiculatum* |
| Tracheo-phyta | Liliopsida | Poales | Poaceae | *Perotis* | *Perotis patens* |
| Tracheo-phyta | Liliopsida | Poales | Poaceae | *Phragmites* | *Phragmites australis* |
| Tracheo-phyta | Liliopsida | Poales | Poaceae | *Phragmites* | *Phragmites mauritianus* |
| Tracheo-phyta | Liliopsida | Poales | Poaceae | *Pogonarthria* | *Pogonarthria squarrosa* |
| Tracheo-phyta | Liliopsida | Poales | Poaceae | *Schmidtia* | *Schmidtia pappophoroides* |
| Tracheo-phyta | Liliopsida | Poales | Poaceae | *Setaria* | *Setaria incrassata* |
| Tracheo-phyta | Liliopsida | Poales | Poaceae | *Setaria* | *Setaria pumila* |
| Tracheo-phyta | Liliopsida | Poales | Poaceae | *Setaria* | *Setaria sagittifolia* |
| Tracheo-phyta | Liliopsida | Poales | Poaceae | *Setaria* | *Setaria sphacelata* |
| Tracheo-phyta | Liliopsida | Poales | Poaceae | *Setaria* | *Setaria verticillata* |
| Tracheo-phyta | Liliopsida | Poales | Poaceae | *Sorghum* | *Sorghum bicolor* |
| Tracheo-phyta | Liliopsida | Poales | Poaceae | *Sorghum* | *Sorghum versicolor* |
| Tracheo-phyta | Liliopsida | Poales | Poaceae | *Sporobolus* | *Sporobolus africanus* |
| Tracheo-phyta | Liliopsida | Poales | Poaceae | *Sporobolus* | *Sporobolus festivus* |
| Tracheo-phyta | Liliopsida | Poales | Poaceae | *Sporobolus* | *Sporobolus fimbriatus* |
| Tracheo-phyta | Liliopsida | Poales | Poaceae | *Sporobolus* | *Sporobolus ioclados* |
| Tracheo-phyta | Liliopsida | Poales | Poaceae | *Sporobolus* | *Sporobolus nitens* |
| Tracheo-phyta | Liliopsida | Poales | Poaceae | *Sporobolus* | *Sporobolus pectinatus* |
| Tracheo-phyta | Liliopsida | Poales | Poaceae | *Sporobolus* | *Sporobolus pyramidalis* |
| Tracheo-phyta | Liliopsida | Poales | Poaceae | *Sporobolus* | *Sporobolus stapfianus* |
| Tracheo-phyta | Liliopsida | Poales | Poaceae | *Stipagrostis* | *Stipagrostis hirtigluma* |
| Tracheo-phyta | Liliopsida | Poales | Poaceae | *Themeda* | *Themeda triandra* |
| Tracheo-phyta | Liliopsida | Poales | Poaceae | *Trachypogon* | *Trachypogon spicatus* |
| Tracheo-phyta | Liliopsida | Poales | Poaceae | *Tragus* | *Tragus berteronianus* |
| Tracheo-phyta | Liliopsida | Poales | Poaceae | *Tricholaena* | *Tricholaena monachne* |
| Tracheo-phyta | Liliopsida | Poales | Poaceae | *Trichoneura* | *Trichoneura grandiglumis* |
| Tracheo-phyta | Liliopsida | Poales | Poaceae | *Tripogon* | *Tripogon minimus* |
| Tracheo-phyta | Liliopsida | Poales | Poaceae | *Urochloa* | *Urochloa brachyura* |
| Tracheo-phyta | Liliopsida | Poales | Poaceae | *Urochloa* | *Urochloa mosambicensis* |
| Tracheo-phyta | Liliopsida | Poales | Poaceae | *Urochloa* | *Urochloa oligotricha* |
| Tracheo-phyta | Liliopsida | Poales | Poaceae | *Urochloa* | *Urochloa panicoides* |
| Tracheo-phyta | Magnoliopsida | Fabales | Polygalaceae | *Polygala* | *Polygala erioptera* |
| Tracheo-phyta | Magnoliopsida | Fabales | Polygalaceae | *Polygala* | *Polygala producta* |
| Tracheo-phyta | Magnoliopsida | Fabales | Polygalaceae | *Polygala* | *Polygala sphenoptera* |
| Tracheo-phyta | Magnoliopsida | Fabales | Polygalaceae | *Polygala* | *Polygala uncinata* |
| Tracheo-phyta | Magnoliopsida | Caryophyllales | Polygonaceae | *Oxygonum* | *Oxygonum delagoense* |
| Tracheo-phyta | Magnoliopsida | Caryophyllales | Polygonaceae | *Oxygonum* | *Oxygonum dregeanum* |
| Tracheo-phyta | Magnoliopsida | Caryophyllales | Polygonaceae | *Oxygonum* | *Oxygonum sinuatum* |
| Tracheo-phyta | Magnoliopsida | Caryophyllales | Polygonaceae | *Persicaria* | *Persicaria decipiens* |
| Tracheo-phyta | Magnoliopsida | Caryophyllales | Portulacaceae | *Portulaca* | *Portulaca hereroensis* |
| Tracheo-phyta | Magnoliopsida | Caryophyllales | Portulacaceae | *Portulaca* | *Portulaca kermesina* |
| Tracheo-phyta | Magnoliopsida | Caryophyllales | Portulacaceae | *Portulaca* | *Portulaca oleracea* |
| Tracheo-phyta | Magnoliopsida | Caryophyllales | Portulacaceae | *Portulaca* | *Portulaca quadrifida* |
| Tracheo-phyta | Polypodiopsida | Polypodiales | Pteridaceae | *Cheilanthes* | *Cheilanthes viridis* |
| Tracheo-phyta | Polypodiopsida | Polypodiales | Pteridaceae | *Pellaea* | *Pellaea calomelanos* |
| Tracheo-phyta | Magnoliopsida | Malpighiales | Putranjivaceae | *Drypetes* | *Drypetes gerrardii* |
| Tracheo-phyta | Magnoliopsida | Ranunculales | Ranunculaceae | *Clematis* | *Clematis brachiata* |
| Tracheo-phyta | Magnoliopsida | Rosales | Rhamnaceae | *Berchemia* | *Berchemia discolor* |
| Tracheo-phyta | Magnoliopsida | Rosales | Rhamnaceae | *Berchemia* | *Berchemia zeyheri* |
| Tracheo-phyta | Magnoliopsida | Rosales | Rhamnaceae | *Ziziphus* | *Ziziphus mucronata* |
| Tracheo-phyta | Magnoliopsida | Gentianales | Rubiaceae | *Agathisanthemum* | *Agathisanthemum bojeri* |
| Tracheo-phyta | Magnoliopsida | Gentianales | Rubiaceae | *Breonadia* | *Breonadia salicina* |
| Tracheo-phyta | Magnoliopsida | Gentianales | Rubiaceae | *Canthium* | *Canthium armatum* |
| Tracheo-phyta | Magnoliopsida | Gentianales | Rubiaceae | *Canthium* | *Canthium ciliatum* |
| Tracheo-phyta | Magnoliopsida | Gentianales | Rubiaceae | *Catunaregam* | *Catunaregam spinosa* |
| Tracheo-phyta | Magnoliopsida | Gentianales | Rubiaceae | *Conostomium* | *Conostomium natalense* |
| Tracheo-phyta | Magnoliopsida | Gentianales | Rubiaceae | *Gardenia* | *Gardenia volkensii* |
| Tracheo-phyta | Magnoliopsida | Gentianales | Rubiaceae | *Hyperacanthus* | *Hyperacanthus amoenus* |
| Tracheo-phyta | Magnoliopsida | Gentianales | Rubiaceae | *Kohautia* | *Kohautia amatymbica* |
| Tracheo-phyta | Magnoliopsida | Gentianales | Rubiaceae | *Kohautia* | *Kohautia caespitosa* |
| Tracheo-phyta | Magnoliopsida | Gentianales | Rubiaceae | *Kohautia* | *Kohautia cynanchica* |
| Tracheo-phyta | Magnoliopsida | Gentianales | Rubiaceae | *Kohautia* | *Kohautia virgata* |
| Tracheo-phyta | Magnoliopsida | Gentianales | Rubiaceae | *Kraussia* | *Kraussia floribunda* |
| Tracheo-phyta | Magnoliopsida | Gentianales | Rubiaceae | *Oldenlandia* | *Oldenlandia herbacea* |
| Tracheo-phyta | Magnoliopsida | Gentianales | Rubiaceae | *Pavetta* | *Pavetta catophylla* |
| Tracheo-phyta | Magnoliopsida | Gentianales | Rubiaceae | *Pavetta* | *Pavetta gardeniifolia* |
| Tracheo-phyta | Magnoliopsida | Gentianales | Rubiaceae | *Pavetta* | *Pavetta lanceolata* |
| Tracheo-phyta | Magnoliopsida | Gentianales | Rubiaceae | *Pavetta* | *Pavetta schumanniana* |
| Tracheo-phyta | Magnoliopsida | Gentianales | Rubiaceae | *Pentodon* | *Pentodon pentandrus* |
| Tracheo-phyta | Magnoliopsida | Gentianales | Rubiaceae | *Psydrax* | *Psydrax obovata* |
| Tracheo-phyta | Magnoliopsida | Gentianales | Rubiaceae | *Pyrostria* | *Pyrostria hystrix* |
| Tracheo-phyta | Magnoliopsida | Gentianales | Rubiaceae | *Richardia* | *Richardia brasiliensis* |
| Tracheo-phyta | Magnoliopsida | Gentianales | Rubiaceae | *Spermacoce* | *Spermacoce senensis* |
| Tracheo-phyta | Magnoliopsida | Gentianales | Rubiaceae | *Tricalysia* | *Tricalysia junodii* |
| Tracheo-phyta | Magnoliopsida | Gentianales | Rubiaceae | *Vangueria* | *Vangueria infausta* |
| Tracheo-phyta | Magnoliopsida | Sapindales | Rutaceae | *Clausena* | *Clausena anisata* |
| Tracheo-phyta | Magnoliopsida | Sapindales | Rutaceae | *Ptaeroxylon* | *Ptaeroxylon obliquum* |
| Tracheo-phyta | Magnoliopsida | Sapindales | Rutaceae | *Vepris* | *Vepris carringtoniana* |
| Tracheo-phyta | Magnoliopsida | Sapindales | Rutaceae | *Zanthoxylum* | *Zanthoxylum humile* |
| Tracheo-phyta | Magnoliopsida | Malpighiales | Salicaceae | *Dovyalis* | *Dovyalis caffra* |
| Tracheo-phyta | Magnoliopsida | Malpighiales | Salicaceae | *Flacourtia* | *Flacourtia indica* |
| Tracheo-phyta | Magnoliopsida | Malpighiales | Salicaceae | *Scolopia* | *Scolopia zeyheri* |
| Tracheo-phyta | Magnoliopsida | Sapindales | Sapindaceae | *Allophylus* | *Allophylus decipiens* |
| Tracheo-phyta | Magnoliopsida | Sapindales | Sapindaceae | *Cardiospermum* | *Cardiospermum corindum* |
| Tracheo-phyta | Magnoliopsida | Sapindales | Sapindaceae | *Cardiospermum* | *Cardiospermum halicacabum* |
| Tracheo-phyta | Magnoliopsida | Ericales | Sapotaceae | *Manilkara* | *Manilkara mochisia* |
| Tracheo-phyta | Magnoliopsida | Lamiales | Scrophulariaceae | *Aptosimum* | *Aptosimum lineare* |
| Tracheo-phyta | Magnoliopsida | Solanales | Solanaceae | *Datura* | *Datura stramonium* |
| Tracheo-phyta | Magnoliopsida | Solanales | Solanaceae | *Solanum* | *Solanum aculeastrum* |
| Tracheo-phyta | Magnoliopsida | Solanales | Solanaceae | *Solanum* | *Solanum catombelense* |
| Tracheo-phyta | Magnoliopsida | Solanales | Solanaceae | *Solanum* | *Solanum delagoense* |
| Tracheo-phyta | Magnoliopsida | Solanales | Solanaceae | *Solanum* | *Solanum incanum* |
| Tracheo-phyta | Magnoliopsida | Solanales | Solanaceae | *Solanum* | *Solanum lichtensteinii* |
| Tracheo-phyta | Magnoliopsida | Solanales | Solanaceae | *Solanum* | *Solanum nigrum* |
| Tracheo-phyta | Magnoliopsida | Solanales | Solanaceae | *Solanum* | *Solanum panduriforme* |
| Tracheo-phyta | Magnoliopsida | Solanales | Solanaceae | *Solanum* | *Solanum seaforthianum* |
| Tracheo-phyta | Magnoliopsida | Solanales | Solanaceae | *Solanum* | *Solanum tettense* |
| Tracheo-phyta | Magnoliopsida | Solanales | Solanaceae | *Withania* | *Withania somnifera* |
| Tracheo-phyta | Magnoliopsida | Lamiales | Stilbaceae | *Nuxia* | *Nuxia oppositifolia* |
| Tracheo-phyta | Magnoliopsida | Caryophyllales | Talinaceae | *Talinum* | *Talinum arnotii* |
| Tracheo-phyta | Magnoliopsida | Caryophyllales | Talinaceae | *Talinum* | *Talinum caffrum* |
| Tracheo-phyta | Magnoliopsida | Caryophyllales | Talinaceae | *Talinum* | *Talinum portulacifolium* |
| Tracheo-phyta | Magnoliopsida | Caryophyllales | Talinaceae | *Talinum* | *Talinum tenuissimum* |
| Tracheo-phyta | Magnoliopsida | Malvales | Thymelaeaceae | *Gnidia* | *Gnidia rubescens* |
| Tracheo-phyta | Magnoliopsida | Rosales | Urticaceae | *Pouzolzia* | *Pouzolzia mixta* |
| Tracheo-phyta | Magnoliopsida | Vahliales | Vahliaceae | *Vahlia* | *Vahlia capensis* |
| Tracheo-phyta | Magnoliopsida | Vitales | Vataceae | *Rhoicissus* | *Rhoicissus revoilii* |
| Tracheo-phyta | Magnoliopsida | Vitales | Vataceae | *Rhoicissus* | *Rhoicissus tridentata* |
| Tracheo-phyta | Liliopsida | Pandanales | Velloziaceae | *Xerophyta* | *Xerophyta retinervis* |
| Tracheo-phyta | Magnoliopsida | Lamiales | Verbenaceae | *Chascanum* | *Chascanum pinnatifidum* |
| Tracheo-phyta | Magnoliopsida | Lamiales | Verbenaceae | *Lantana* | *Lantana camara* |
| Tracheo-phyta | Magnoliopsida | Lamiales | Verbenaceae | *Lantana* | *Lantana rugosa* |
| Tracheo-phyta | Magnoliopsida | Lamiales | Verbenaceae | *Lippia* | *Lippia javanica* |
| Tracheo-phyta | Magnoliopsida | Lamiales | Verbenaceae | *Priva* | *Priva cordifolia* |
| Tracheo-phyta | Magnoliopsida | Lamiales | Verbenaceae | *Priva* | *Priva meyeri* |
| Tracheo-phyta | Magnoliopsida | Lamiales | Verbenaceae | *Verbena* | *Verbena bonariensis* |
| Tracheo-phyta | Magnoliopsida | Malpighiales | Violaceae | *Hybanthus* | *Hybanthus enneaspermus* |
| Tracheo-phyta | Magnoliopsida | Vitales | Vitaceae | *Cissus* | *Cissus cornifolia* |
| Tracheo-phyta | Magnoliopsida | Vitales | Vitaceae | *Cissus* | *Cissus quadrangularis* |
| Tracheo-phyta | Magnoliopsida | Vitales | Vitaceae | *Cissus* | *Cissus rotundifolia* |
| Tracheo-phyta | Magnoliopsida | Vitales | Vitaceae | *Cyphostemma* | *Cyphostemma cirrhosum* |
| Tracheo-phyta | Magnoliopsida | Vitales | Vitaceae | *Cyphostemma* | *Cyphostemma puberulum* |
| Tracheo-phyta | Magnoliopsida | Vitales | Vitaceae | *Cyphostemma* | *Cyphostemma schlechteri* |
| Tracheo-phyta | Magnoliopsida | Vitales | Vitaceae | *Cyphostemma* | *Cyphostemma woodii* |
| Tracheo-phyta | Magnoliopsida | Santalales | Ximeniaceae | *Ximenia* | *Ximenia americana* |
| Tracheo-phyta | Magnoliopsida | Santalales | Ximeniaceae | *Ximenia* | *Ximenia caffra* |
| Tracheo-phyta | Magnoliopsida | Zygophyllales | Zygophyllaceae | *Balanites* | *Balanites maughamii* |
| Tracheo-phyta | Magnoliopsida | Zygophyllales | Zygophyllaceae | *Tribulus* | *Tribulus terrestris* |
| Tracheo-phyta | Magnoliopsida | Zygophyllales | Zygophyllaceae | *Tribulus* | *Tribulus zeyheri* |
