# Supplementary material for: Introducing an rbcL and a trnL reference library to aid in the metabarcoding analysis of foraged plants from two semi-arid eastern South African savanna bioregions
Source: PLoS One. 2023 May 19;18(5):e0286144. doi: 10.1371/journal.pone.0286144 (PMC10198553; doi:10.1371/journal.pone.0286144)
Supplement: S2 Table — (DOCX) [file pone.0286144.s002.docx]

Supplementary Table 2: List of species, for which *rbc*L and/or *trn*L barcode sequences were available in public databases, included in the *rbc*L and *trn*L reference datasets with their selected accession numbers (GenBank) and/or Process IDs (BOLD).

| Family | Genus | Species | Bar-code | Accession/Process ID | | |
| --- | --- | --- | --- | --- | --- | --- |
| Acanthaceae | Asystasia | *Asystasia mysorensis* | trnL | MK261330 | MK261517 |  |
| Acanthaceae | Asystasia | *Asystasia mysorensis* | rbcL | MK285106 |  |  |
| Acanthaceae | *Asystasia* | *Asystasia sp.* | trnL | KR738111 | KR737908 | KR737825 |
| Acanthaceae | *Asystasia* | *Asystasia sp.* | rbcL | KR736918 | KR736699 | KR736619 |
| Acanthaceae | *Barleria* | *Barleria elegans* | trnL | MZ461549 |  |  |
| Acanthaceae | *Barleria* | *Barleria elegans* | rbcL | MZ461573 |  |  |
| Acanthaceae | *Barleria* | *Barleria prionitis* | trnL | KT075002 | MZ461550 |  |
| Acanthaceae | *Barleria* | *Barleria prionitis* | rbcL | JQ231001 | MZ461574 |  |
| Acanthaceae | *Blepharis* | *Blepharis innocua* | trnL | MZ461551 |  |  |
| Acanthaceae | *Blepharis* | *Blepharis innocua* | rbcL | MZ461575 |  |  |
| Acanthaceae | *Blepharis* | *Blepharis integrifolia* | trnL | DQ054861 | DQ054860 |  |
| Acanthaceae | *Blepharis* | *Blepharis integrifolia* | rbcL | MZ461576 |  |  |
| Acanthaceae | *Blepharis* | *Blepharis subvolubilis* | trnL | DQ054864 |  |  |
| Acanthaceae | *Crabbea* | *Crabbea velutina* | trnL | MK261649 | MK261682 | MZ461555 |
| Acanthaceae | *Crabbea* | *Crabbea velutina* | rbcL | MZ461579 |  |  |
| Acanthaceae | *Ecbolium* | *Ecbolium glabratum* | trnL | MZ461556 |  |  |
| Acanthaceae | *Ecbolium* | *Ecbolium glabratum* | rbcL | MZ461580 |  |  |
| Acanthaceae | *Elytraria* | *Elytraria acaulis* | rbcL | GENG1064-15.rbcL | GENG1085-15.rbcL |  |
| Acanthaceae | *Hygrophila* | *Hygrophila auriculata* | rbcL | GENG235-14.rbcL | TCHDC029-19.rbcLa | TRM288-15.rbcL |
| Acanthaceae | *Hypoestes* | *Hypoestes forskaolii* | trnL | MK261658 | MK261738 | MK261478 |
| Acanthaceae | *Hypoestes* | *Hypoestes forskaolii* | rbcL | MK285173 | MK285223 | KR736995 |
| Acanthaceae | *Justicia* | *Justicia anagalloides* | trnL | KR737885 | KR737996 | KR738092 |
| Acanthaceae | *Justicia* | *Justicia anagalloides* | rbcL | KR736679 | KR736795 | KR737420 |
| Acanthaceae | *Justicia* | *Justicia betonica* | trnL | KP744162 | KT075011 |  |
| Acanthaceae | *Justicia* | *Justicia betonica* | rbcL | KF669390 |  |  |
| Acanthaceae | *Justicia* | *Justicia flava* | trnL | MK282374 | MZ461562 |  |
| Acanthaceae | *Justicia* | *Justicia flava* | rbcL | MZ461584 |  |  |
| Acanthaceae | *Justicia* | *Justicia protracta* | trnL | MZ461563 |  |  |
| Acanthaceae | *Justicia* | *Justicia protracta* | rbcL | MZ461585 |  |  |
| Acanthaceae | *Monechma* | *Monechma debile* | trnL | KR738598 | KR738710 | KR737588 |
| Acanthaceae | *Monechma* | *Monechma debile* | rbcL | KR736640 | KR737446 | KR737565 |
| Acanthaceae | *Peristrophe* | *Peristrophe cernua* | rbcL | AM234782 |  |  |
| Acanthaceae | *Rhinacanthus* | *Rhinacanthus xerophilus* | trnL | MK282400 |  |  |
| Acanthaceae | *Ruellia* | *Ruellia patula* | trnL | KR738685 | KR738215 | KR738247 |
| Acanthaceae | *Ruellia* | *Ruellia patula* | rbcL | KR736978 | KR737038 | KR737070 |
| Acanthaceae | *Ruellia* | *Ruellia prostrata* | trnL | KR738713 | KR737762 | KR737781 |
| Acanthaceae | *Ruellia* | *Ruellia prostrata* | rbcL | MK285130 | KR737568 | KR736549 |
| Acanthaceae | *Thunbergia* | *Thunbergia alata* | rbcL | KR736371 | KR737000 | KR737099 |
| Aizoaceae | *Aizoon* | *Aizoon canariense* | trnL | KX197553 | HE585091 |  |
| Aizoaceae | *Aizoon* | *Aizoon canariense* | rbcL | MF694659 | KX282515 | MK097151 |
| Aizoaceae | *Zaleya* | *Zaleya pentandra* | trnL | KR738493 |  |  |
| Aizoaceae | *Zaleya* | *Zaleya pentandra* | rbcL | KR737328 |  |  |
| Amaranthaceae | Achyranthes | *Achyranthes aspera* | trnL | MK186959 | KR738556 | KR738119 |
| Amaranthaceae | Achyranthes | *Achyranthes aspera* | rbcL | MF694653 | AY270048 | MK959201 |
| Amaranthaceae | Achyropsis | *Achyropsis leptostachya* | trnL | LT993028 |  |  |
| Amaranthaceae | Aerva | *Aerva leucura* | rbcL | PNG209-18.rbcL | PNG499-18.rbcL |  |
| Amaranthaceae | *Alternanthera* | *Alternanthera pungens* | trnL | MK261727 | KR738256 |  |
| Amaranthaceae | *Alternanthera* | *Alternanthera pungens* | rbcL | KR737216 | KR737079 | KR736427 |
| Amaranthaceae | *Alternanthera* | *Alternanthera sessilis* | trnL | MK261546 |  |  |
| Amaranthaceae | *Alternanthera* | *Alternanthera sessilis* | rbcL | IPSUB108-18.rbcL | MK285216 | KJ773257 |
| Amaranthaceae | *Amaranthus* | *Amaranthus hybridus* | trnL | MK261721 | MK261365 | MK261389 |
| Amaranthaceae | *Amaranthus* | *Amaranthus hybridus* | rbcL | MK285206 | MK285186 |  |
| Amaranthaceae | *Amaranthus* | *Amaranthus praetermissus* | trnL | MG685468 |  |  |
| Amaranthaceae | *Amaranthus* | *Amaranthus thunbergii* | trnL | MG685487 | MG685486 |  |
| Amaranthaceae | *Celosia* | *Celosia trigyna* | trnL | MK261542 | MK261316 | MK187005 |
| Amaranthaceae | *Chenopodium* | *Chenopodium (Dysphania) ambrosioides* | trnL | MF073670 | MF073668 | MF073667 |
| Amaranthaceae | *Chenopodium* | *Chenopodium (Dysphania) ambrosioides* | rbcL | MH658693 | MK525735 | MK285083 |
| Amaranthaceae | *Chenopodium* | *Chenopodium album* | trnL | MF073603 | MF073605 | MF073636 |
| Amaranthaceae | *Chenopodium* | *Chenopodium album* | rbcL | JX848451 | HM849888 | MF69468 |
| Amaranthaceae | *Cyathula* | *Cyathula lanceolata* | trnL | LT993035 | LT995128 |  |
| Amaranthaceae | *Gomphrena* | *Gomphrena celosioides* | trnL | LT993038 | MF039680 | MK187073 |
| Amaranthaceae | *Gomphrena* | *Gomphrena celosioides* | rbcL | MH049891 | MH049893 | MH049894 |
| Amaranthaceae | *Guilleminea* | *Guilleminea densa* | trnL | LT993061 | EF688764 |  |
| Amaranthaceae | *Guilleminea* | *Guilleminea densa* | rbcL | AY270091 |  | KP149536 |
| Amaranthaceae | *Kyphocarpa* | *Kyphocarpa angustifolia* | trnL | LT993017 | EF688778 |  |
| Amaranthaceae | *Kyphocarpa* | *Kyphocarpa angustifolia* | rbcL | JQ693464 |  |  |
| Amaranthaceae | *Pupalia* | *Pupalia lappacea* | trnL | LT993050 | LT993042 | LT993040 |
| Amaranthaceae | *Pupalia* | *Pupalia lappacea* | rbcL | AY270122 | GENG454-14.rbcL | PNG503-18.rbcL |
| Amaranthaceae | *Sericorema* | *Sericorema remotiflora* | trnL | LT993031 | EF688788 |  |
| Amaryllidaceae | *Ammocharis* | *Ammocharis coranica* | trnL | JX464335 | AY139152 |  |
| Amaryllidaceae | *Boophane* | *Boophane disticha* | trnL | JX464336 |  |  |
| Amaryllidaceae | *Boophone* | *Boophone disticha* | rbcL | JQ025023 |  |  |
| Amaryllidaceae | *Crinum* | *Crinum buphanoides* | trnL | AY139157 | EF119732 | EU523774 |
| Amaryllidaceae | *Crinum* | *Crinum macowanii* | trnL | KR738102 | KR737706 | KR737604 |
| Amaryllidaceae | *Crinum* | *Crinum macowanii* | rbcL | KR736910 | KR736485 | KR736375 |
| Amaryllidaceae | *Crinum* | *Crinum moorei* | trnL | JX464342 |  |  |
| Amaryllidaceae | *Crinum* | *Crinum moorei* | rbcL | KY627303 |  |  |
| Amaryllidaceae | *Tulbaghia* | *Tulbaghia leucantha* | trnL | KU692108 | KU692107 |  |
| Anacardiaceae | *Lannea* | *Lannea discolor* | trnL | PNG347-18.trnL-F |  |  |
| Anacardiaceae | *Lannea* | *Lannea discolor* | rbcL | JF265496 | PNG347-18.rbcL |  |
| Anacardiaceae | *Lannea* | *Lannea edulis* | rbcL | JF265497 | JQ025059 | JX572710 |
| Anacardiaceae | *Lannea* | *Lannea schweinfurthii* | trnL | AY594552 |  |  |
| Anacardiaceae | *Lannea* | *Lannea schweinfurthii* | rbcL | GU935430 | JF265498 | JX572711 |
| Anacardiaceae | *Ozoroa* | *Ozoroa albicans* | rbcL | KF147498 |  |  |
| Anacardiaceae | *Ozoroa* | *Ozoroa engleri* | rbcL | JF265536 | JX572820 |  |
| Anacardiaceae | *Ozoroa* | *Ozoroa insignis* | trnL | AY594444 |  |  |
| Anacardiaceae | *Ozoroa* | *Ozoroa insignis* | rbcL | KU568163 |  |  |
| Anacardiaceae | *Ozoroa* | *Ozoroa paniculosa* | rbcL | JX572822 |  |  |
| Anacardiaceae | *Sclerocarya* | *Sclerocarya birrea* | trnL | KR081762 | KR081763 | KC479214 |
| Anacardiaceae | *Sclerocarya* | *Sclerocarya birrea* | rbcL | MN166719 | MN216516 | MN216531 |
| Anacardiaceae | *Searsia* | *Searsia gueinzii* | rbcL | EU213504 | EU213506 | EU213505 |
| Anacardiaceae | *Searsia* | *Searsia pentheri* | rbcL | JX572976 | JF265592 |  |
| Anacardiaceae | *Searsia* | *Searsia pyroides* | trnL | AY640467 | FJ946002 |  |
| Anacardiaceae | *Searsia* | *Searsia pyroides* | rbcL | JF265593 | JX572929 | JX572977 |
| Annonaceae | *Hexalobus* | *Hexalobus monopetalus* | rbcL | JF265472 | JX572672 |  |
| Annonaceae | *Monanthotaxis* | *Monanthotaxis caffra* | rbcL | MF353750 | JF265520 |  |
| Apiaceae | *Centella* | *Centella asiatica* | rbcL | MK905059 | MK905037 | MK905036 |
| Apiaceae | *Pappea* | *Pappea capensis* | trnL | KF180245 | KF180246 | KF180320 |
| Apiaceae | *Pappea* | *Pappea capensis* | rbcL | AM235130 | JF265540 | JX572827 |
| Apocynaceae | Adenium | *Adenium multiflorum* | rbcL | JF265270 | JX572243 |  |
| Apocynaceae | Adenium | *Adenium swazicum* | rbcL | JF265271 | JX572244 |  |
| Apocynaceae | *Carissa* | *Carissa bispinosa* | trnL | AF214172 |  |  |
| Apocynaceae | *Carissa* | *Carissa bispinosa* | rbcL | JF265326 | JX572375 | AJ419738 |
| Apocynaceae | *Carissa* | *Carissa edulis* | rbcL | JX572376 | JF265327 | MT231387 |
| Apocynaceae | *Catharanthus* | *Catharanthus roseus* | trnL | AF214175 |  |  |
| Apocynaceae | *Catharanthus* | *Catharanthus roseus* | rbcL | MN125628 | MH549765 | MH069755 |
| Apocynaceae | *Cryptolepis* | *Cryptolepis oblongifolia* | trnL | DQ916872 | AF214422 | KT280301 |
| Apocynaceae | *Cynanchum* | *Cynanchum viminale* | trnL | LN896759 | HG530580.2 | LN896724 |
| Apocynaceae | *Cynanchum* | *Cynanchum viminale* | rbcL | JQ025085 | KR736494 | KR736504 |
| Apocynaceae | *Fockea* | *Fockea angustifolia* | trnL | AM233365 | AM233366 | AM233367 |
| Apocynaceae | *Gomphocarpus* | *Gomphocarpus tomentosus* | trnL | AM295753 |  | AM295755 |
| Apocynaceae | *Pentarrhinum* | *Pentarrhinum insipidum* | trnL | AJ410233 | AJ410232 | AJ410234 |
| Apocynaceae | *Pentarrhinum* | *Pentarrhinum insipidum* | rbcL | HG530565 |  |  |
| Apocynaceae | *Pergularia* | *Pergularia daemia* | trnL | AJ290891 | AJ290893 |  |
| Apocynaceae | *Pergularia* | *Pergularia daemia* | rbcL | JQ025071 | API105-12.rbcL | GENG635-14.rbcL |
| Apocynaceae | *Raphionacme* | *Raphionacme elata* | trnL | AJ581816 | AJ581814 |  |
| Apocynaceae | *Riocreuxia* | *Riocreuxia torulosa* | trnL | LT595694 | LT595695 | LT595681 |
| Apocynaceae | *Riocreuxia* | *Riocreuxia torulosa* | rbcL | AM234841 |  |  |
| Apocynaceae | *Secamone* | *Secamone parvifolia* | trnL | HE805520 |  |  |
| Apocynaceae | *Stapelia* | *Stapelia gigantea* | trnL | KF678326 | KF678044 |  |
| Apocynaceae | *Stapelia* | *Stapelia gigantea* | rbcL | JQ025094 |  |  |
| Apocynaceae | *Stomatostemma* | *Stomatostemma monteiroae* | trnL | AJ431780 | AJ581833 | AF214428 |
| Apocynaceae | *Tacazzea* | *Tacazzea apiculata* | trnL | DQ221206 | AY899972 | DQ221164 |
| Apocynaceae | *Tacazzea* | *Tacazzea apiculata* | rbcL | AJ419764 |  |  |
| Arecaceae | *Hyphaene* | *Hyphaene coriacea* | trnL | AM903235 |  |  |
| Arecaceae | *Hyphaene* | *Hyphaene coriacea* | rbcL | AM903195 | AY012470 | MG437594 |
| Arecaceae | *Phoenix* | *Phoenix reclinata* | trnL | AJ241272 | MK261397 |  |
| Arecaceae | *Phoenix* | *Phoenix reclinata* | rbcL | AJ404767 | MG437546 | GU135280 |
| Aristolochiaceae | *Aristolochia* | *Aristolochia elegans* | trnL | AY689164 | AY781544 |  |
| Aristolochiaceae | *Aristolochia* | *Aristolochia elegans* | rbcL | INB163-12.rbcLa |  |  |
| Asparagaceae | *Albuca* | *Albuca setosa* | rbcL | AM902292 |  |  |
| Asparagaceae | *Asparagus* | *Asparagus africanus* | trnL | MH092175 | MK186979 | KJ774039 |
| Asparagaceae | *Asparagus* | *Asparagus africanus* | rbcL | JQ014166 | PNG388-18.rbcL | PNG539-18.rbcL |
| Asparagaceae | *Asparagus* | *Asparagus crassicladus* | rbcL | JQ014167 |  |  |
| Asparagaceae | *Asparagus* | *Asparagus falcatus* | trnL | KR737978 | AF508514 | MH092174 |
| Asparagaceae | *Asparagus* | *Asparagus falcatus* | rbcL | MH630260 | KR737442 | KR737020 |
| Asparagaceae | *Asparagus* | *Asparagus plumosus (setaceus)* | trnL | KJ774038 |  |  |
| Asparagaceae | *Asparagus* | *Asparagus plumosus (setaceus)* | rbcL | JQ734483 | JQ734482 | KX783831 |
| Asparagaceae | *Asparagus* | *Asparagus retrofractus* | trnL | KJ774035 |  |  |
| Asparagaceae | *Asparagus* | *Asparagus retrofractus* | rbcL | KP110202 |  |  |
| Asparagaceae | *Asparagus* | *Asparagus suaveolens* | rbcL | JQ014168 |  |  |
| Asparagaceae | *Chlorophytum* | *Chlorophytum galpinii* | trnL | EU000031 |  |  |
| Asparagaceae | *Dipcadi* | *Dipcadi viride* | trnL | AM902446 |  |  |
| Asparagaceae | *Dipcadi* | *Dipcadi viride* | rbcL | AM902300 |  |  |
| Asparagaceae | *Eriospermum* | *Eriospermum flagelliforme* | rbcL | HM640475 |  |  |
| Asparagaceae | *Ledebouria* | *Ledebouria revoluta* | trnL | AJ507944 | AJ507945 | Z99145 |
| Asparagaceae | *Ledebouria* | *Ledebouria revoluta* | rbcL | MF349645 |  |  |
| Asparagaceae | *Ornithogalum* | *Ornithogalum seineri* | trnL | AM902461 |  |  |
| Asparagaceae | *Ornithogalum* | *Ornithogalum seineri* | rbcL | AM902314 |  |  |
| Asparagaceae | *Sansevieria* | *Sansevieria hyacinthoides* | trnL | MH092217 |  |  |
| Asparagaceae | *Sansevieria* | *Sansevieria hyacinthoides* | rbcL | GU135195 | JQ025084 | GENG747-14.rbcL |
| Asparagaceae | *Sansevieria* | *Sansevieria pearsonii* | trnL | MH092220 |  |  |
| Asparagaceae | *Urginea* | *Drimia altissima* | trnL | AM410909 |  |  |
| Asparagaceae | *Urginea* | *Drimia altissima* | rbcL | JQ273914 | HM640505 | HM640515 |
| Asphodelaceae | *Aloe* | *Aloe chabaudii* | rbcL | KNPA978-09.rbcLa |  |  |
| Asphodelaceae | *Aloe* | *Aloe marlothii* | rbcL | JF265285 |  |  |
| Asphodelaceae | *Bulbine* | *Bulbine abyssinica* | trnL | MK261638 |  |  |
| Asphodelaceae | *Bulbine* | *Bulbine abyssinica* | rbcL | MK285204 | MK285205 |  |
| Asteraceae | Acanthospermum | *Acanthospermum hispidum* | trnL | MK261615 |  |  |
| Asteraceae | Acanthospermum | *Acanthospermum hispidum* | rbcL | MG224060 | MG221964 |  |
| Asteraceae | Ageratina | *Ageratina altissima* | rbcL | KP643669 | KJ841084 | HQ590095 |
| Asteraceae | *Ageratum* | *Ageratum conyzoides* | trnL | KR738616 | KR738644 | KR738674 |
| Asteraceae | *Ageratum* | *Ageratum conyzoides* | rbcL | MG707331 | KR737496 | KR737527 |
| Asteraceae | *Ambrosia* | *Ambrosia artemisiifolia* | trnL | MG709415 |  |  |
| Asteraceae | *Ambrosia* | *Ambrosia artemisiifolia* | rbcL | MN812527 | MT642638 | MK900737 |
| Asteraceae | *Aspilia* | *Aspilia mossambicensis* | trnL | MK186981 |  |  |
| Asteraceae | *Aspilia* | *Aspilia mossambicensis* | rbcL | KR736520 | KR737379 | KR736733 |
| Asteraceae | *Baccharoides* | *Baccharoides adoensis* | trnL | EF155833 |  |  |
| Asteraceae | *Bidens* | *Bidens biternata* | rbcL | MK903548 | MH168544 |  |
| Asteraceae | *Bidens* | *Bidens pilosa* | rbcL | MF688949 | MH658694 | KU958561 |
| Asteraceae | *Cirsium* | *Cirsium vulgare* | trnL | MN314896 | MN919154 | AF129826 |
| Asteraceae | *Cirsium* | *Cirsium vulgare* | rbcL | HM849899 | MT189198 | HQ590038 |
| Asteraceae | *Conyza* | *Conyza scabrida* | rbcL | AM234861 |  |  |
| Asteraceae | *Geigeria* | *Geigeria burkei* | trnL | LN607098 |  |  |
| Asteraceae | *Geigeria* | *Geigeria ornativa* | trnL | LN607105 |  |  |
| Asteraceae | *Hirpicium* | *Hirpicium bechuanense* | trnL | EU527288 | JQ220177 | MG833204 |
| Asteraceae | *Parthenium* | *Parthenium hysterophorus* | trnL | MH017920 | AY216215 | MH017895 |
| Asteraceae | *Parthenium* | *Parthenium hysterophorus* | rbcL | AY215155 | JQ933433 | KJ773721 |
| Asteraceae | *Pegolettia* | *Pegolettia senegalensis* | trnL | LN607149 |  |  |
| Asteraceae | *Pluchea* | *Pluchea bojeri* | trnL | LN607155 |  |  |
| Asteraceae | *Pseudoconyza* | *Pseudoconyza viscosa* | trnL | LN607183 | EF211021 |  |
| Asteraceae | *Pseudognaphalium* | *Pseudognaphalium luteoalbum* | trnL | FN645763 |  |  |
| Asteraceae | *Pseudognaphalium* | *Pseudognaphalium luteoalbum* | rbcL | KT626769 | HM850034 | KP149531 |
| Asteraceae | *Schkuhria* | *Schkuhria pinnata* | trnL | KR738521 | KR738458 |  |
| Asteraceae | *Schkuhria* | *Schkuhria pinnata* | rbcL | KR737289 | KR737362 |  |
| Asteraceae | *Schkuhria* | *Schkuhria pinnata* | rbcL | KR737362 | KR737289 |  |
| Asteraceae | *Sphaeranthus* | *Sphaeranthus incisus* | trnL | LN607208 |  |  |
| Asteraceae | *Tagetes* | *Tagetes minuta* | trnL | KR738171 | KR738696 | KR738034 |
| Asteraceae | *Tagetes* | *Tagetes minuta* | rbcL | MF694781 | KR736990 | KR736837 |
| Asteraceae | *Tridax* | *Tridax procumbens* | rbcL | JQ933511 | KJ773956 | MH767506 |
| Asteraceae | *Vernonia* | *Vernonia (Hilliardiella) oligocephala* | trnL | EF155870 |  |  |
| Asteraceae | *Vernonia* | *Vernonia (Hilliardiella) oligocephala* | rbcL | JQ025103 | JQ025104 | MF688955 |
| Asteraceae | *Vernonia* | *Vernonia (Linzia) glabra* | trnL | JN715856 | JN715857 |  |
| Asteraceae | *Vernonia* | *Vernonia (Parapolydora) fastigiata* | trnL | EF155885 | EF155905 |  |
| Asteraceae | *Vernonia* | *Vernonia (Polydora) poskeana* | trnL | JN837198 |  |  |
| Asteraceae | *Vernonia* | *Vernonia colorata* | trnL | JN715853 |  |  |
| Asteraceae | *Vernonia* | *Vernonia colorata* | rbcL | JF265649 | PNG290-18.rbcL |  |
| Asteraceae | *Xanthium* | *Xanthium strumarium* | trnL | MK261333 | MK261387 | MH985486 |
| Asteraceae | *Xanthium* | *Xanthium strumarium* | rbcL | MK285087 | MK285086 | MH049963 |
| Asteraceae | *Zinnia* | *Zinnia peruviana* | rbcL | MT189265 | MH028872 |  |
| Bignoniaceae | *Kigelia* | *Kigelia africana* | trnL | AF034880 | EF105072 |  |
| Bignoniaceae | *Kigelia* | *Kigelia africana* | rbcL | JN114810 | JN114811 | KY988316 |
| Bignoniaceae | *Rhigozum* | *Rhigozum zambesiacum* | trnL | KNPA552-09.rbcLa | JF265571 | JX572924 |
| Burseraceae | *Commiphora* | *Commiphora africana* | trnL | MK261492 |  |  |
| Burseraceae | *Commiphora* | *Commiphora africana* | rbcL | JX572461 | JF265357 |  |
| Burseraceae | *Commiphora* | *Commiphora angolensis* | trnL | KM516812 |  |  |
| Burseraceae | *Commiphora* | *Commiphora edulis* | trnL | FJ466480 | GU246096 |  |
| Burseraceae | *Commiphora* | *Commiphora edulis* | rbcL | FJ466630 | JF265358 | JX572462 |
| Burseraceae | *Commiphora* | *Commiphora glandulosa* | rbcL | JF265359 |  |  |
| Burseraceae | *Commiphora* | *Commiphora mollis* | rbcL | JF265362 | JX572464 |  |
| Burseraceae | *Commiphora* | *Commiphora pyracanthoides* | rbcL | JX572465 | JF265364 |  |
| Cactaceae | *Opuntia* | *Opuntia ficus-indica* | trnL | MK261524 | JF712757 | JF712756 |
| Cactaceae | *Opuntia* | *Opuntia ficus-indica* | rbcL | MK285242 | SDH786-14.rbcLa | KR737121 |
| Cactaceae | *Opuntia* | *Opuntia stricta* | trnL | JF712826 |  |  |
| Cactaceae | *Opuntia* | *Opuntia stricta* | rbcL | KJ773705 | JQ412402 |  |
| Campanula-ceae | *Wahlenbergia* | *Wahlenbergia krebsii* | trnL | KC147797 | HQ823521 |  |
| Campanula-ceae | *Wahlenbergia* | *Wahlenbergia undulata* | trnL | HQ823547 |  |  |
| Cannabaceae | *Trema* | *Trema orientale* | trnL | KY827124 | KY827125 | AY488689 |
| Cannabaceae | *Trema* | *Trema orientale* | rbcL | MH778733 | MH767744 | KY827166 |
| Capparaceae | *Boscia* | *Boscia albitrunca* | rbcL | JF265307 | KNPA495-09.rbcLa | JX572338 |
| Capparaceae | *Boscia* | *Boscia angustifolia* | trnL | KR738286 | KR738042 | KR737829 |
| Capparaceae | *Boscia* | *Boscia angustifolia* | rbcL | KR736845 | KR736623 | KR736458 |
| Capparaceae | *Boscia* | *Boscia mossambicensis* | rbcL | JX572340 | JF265310 | MN166695 |
| Capparaceae | *Capparis* | *Capparis sepiaria* | rbcL | JF265324 | KY558529 | JX572373 |
| Capparaceae | *Capparis* | *Capparis tomentosa* | trnL | KR737813 | KR738333 | KR737775 |
| Capparaceae | *Capparis* | *Capparis tomentosa* | rbcL | JF265325 | JX572374 | JQ898576 |
| Capparaceae | *Maerua* | *Maerua angolensis* | trnL | KR738053 | KR738016 | KR737752 |
| Capparaceae | *Maerua* | *Maerua angolensis* | rbcL | KR736859 | KR736816 | KR736539 |
| Capparaceae | *Maerua* | *Maerua juncea* | rbcL | JF265510 | JX572747 |  |
| Capparaceae | *Maerua* | *Maerua parvifolia* | rbcL | JF265511 |  |  |
| Caryophylla-ceae | *Pollichia* | *Pollichia campestris* | trnL | KR738142 | KR738068 | KR737857 |
| Caryophylla-ceae | *Pollichia* | *Pollichia campestris* | rbcL | KR736875 | KR737200 | KR736651 |
| Celastraceae | *Elaeodendron* | *Elaeodendron transvaalense* | trnL | MZ461557 |  |  |
| Celastraceae | *Elaeodendron* | *Elaeodendron transvaalense* | rbcL | AM234953 | JF265407 | JX572547 |
| Celastraceae | *Gymnosporia* | *Gymnosporia heterophylla* | rbcL | JF265458 |  |  |
| Celastraceae | *Gymnosporia* | *Gymnosporia senegalensis* | trnL | EU328782 | EU328834 | EU328860 |
| Celastraceae | *Gymnosporia* | *Gymnosporia senegalensis* | rbcL | JF265463 | JX572661 | AY380353 |
| Celastraceae | *Hippocratea* | *Pristimera longipetiolata* | trnL | HM230232 | HM230276 |  |
| Celastraceae | *Hippocratea* | *Pristimera longipetiolata* | rbcL | JQ025074 | JF265558 | JX572876 |
| Celastraceae | *Maytenus* | *Maytenus acuminata* | rbcL | JX572760 |  |  |
| Celastraceae | *Mystroxylon* | *Mystroxylon aethiopicum* | trnL | MK261627 | DQ217490 |  |
| Celastraceae | *Mystroxylon* | *Mystroxylon aethiopicum* | rbcL | AM234958 | JF265523 | JX572790 |
| Celastraceae | *Robsonodendron* | *Robsonodendron eucleiforme* | trnL | DQ217468 | DQ217495 |  |
| Celastraceae | *Robsonodendron* | *Robsonodendron eucleiforme* | rbcL | JX572935 |  |  |
| Cleomaceae | *Cleome* | *Cleome hirta* | trnL | KR737787 | KR737898 | KR737852 |
| Cleomaceae | *Cleome* | *Cleome hirta* | rbcL | KU739620 | KR736690 | KR736577 |
| Cleomaceae | *Cleome* | *Cleome monophylla* | trnL | KR738280 | KR738358 | KR737778 |
| Cleomaceae | *Cleome* | *Cleome monophylla* | rbcL | KT588792 | KT588793 | KR737104 |
| Cleomaceae | *Gynandropsis* | *Cleome (Gynandropsis) gynandra* | trnL | KR738198 |  |  |
| Cleomaceae | *Gynandropsis* | *Cleome (Gynandropsis) gynandra* | rbcL | KU739630 | KR737017 |  |
| Colchicaceae | *Camptorrhiza* | *Camptorrhiza strumosa* | rbcL | KC796864 |  |  |
| Colchicaceae | *Gloriosa* | *Gloriosa superba* | trnL | AJ551362 | EU044686 | AJ551361 |
| Colchicaceae | *Gloriosa* | *Gloriosa superba* | rbcL | KC796869 | KR737424 | KR737353 |
| Combretaceae | *Combretum* | *Combretum apiculatum* | rbcL | KC158503 | EU338143 | EU338142 |
| Combretaceae | *Combretum* | *Combretum collinum* | rbcL | EU338158 | EU338159 | FJ381790 |
| Combretaceae | *Combretum* | *Combretum erythrophyllum* | rbcL | EU338133 | JF265347 | JX572439 |
| Combretaceae | *Combretum* | *Combretum hereroense* | rbcL | KC158530 | EU213457 | EU338138 |
| Combretaceae | *Combretum* | *Combretum imberbe* | trnL | PNG471-18.trnL-F |  |  |
| Combretaceae | *Combretum* | *Combretum imberbe* | rbcL | EU338155 | EU338154 | JF265349 |
| Combretaceae | *Combretum* | *Combretum microphyllum* | rbcL | EU338130 | JF265351 | KC158531 |
| Combretaceae | *Combretum* | *Combretum molle* | trnL | MK261347 |  |  |
| Combretaceae | *Combretum* | *Combretum molle* | rbcL | EU338146 | EU338147 | JF265352 |
| Combretaceae | *Combretum* | *Combretum mossambicense* | rbcL | EU338131 | JF265353 | JX572448 |
| Combretaceae | *Combretum* | *Combretum zeyheri* | trnL | MZ461553 |  |  |
| Combretaceae | *Combretum* | *Combretum zeyheri* | rbcL | EU338166 | JF265356 | MZ461578 |
| Combretaceae | *Terminalia* | *Terminalia phanerophlebia* | rbcL | EU338119 | JX573038 | JF265624 |
| Combretaceae | *Terminalia* | *Terminalia prunioides* | rbcL | EU338120 | JF265625 |  |
| Combretaceae | *Terminalia* | *Terminalia sericea* | rbcL | EU338121 | JF265626 | JX573041 |
| Commelina-ceae | *Commelina* | *Commelina africana* | trnL | KR738448 | EF092863 |  |
| Commelina-ceae | *Commelina* | *Commelina africana* | rbcL | JQ025036 | KR737249 | KR737279 |
| Commelina-ceae | *Commelina* | *Commelina benghalensis* | trnL | KR738418 | KR738678 | KR737616 |
| Commelina-ceae | *Commelina* | *Commelina benghalensis* | rbcL | KR737245 | MH767487 | JF941286 |
| Commelina-ceae | *Commelina* | *Commelina diffusa* | rbcL | KJ773395 | KU564782 |  |
| Commelina-ceae | *Commelina* | *Commelina eckloniana* | trnL | EF092851 | EF092853 |  |
| Commelina-ceae | *Commelina* | *Commelina erecta* | trnL | KR737593 | EF092858 |  |
| Commelina-ceae | *Commelina* | *Commelina erecta* | rbcL | KY626767 | MK525585 | JQ591125 |
| Commelina-ceae | *Murdannia* | *Murdannia simplex* | trnL | EF092845 |  |  |
| Commelina-ceae | *Murdannia* | *Murdannia simplex* | rbcL | GENG1253-15.rbcL |  |  |
| Convolvulaceae | *Convolvulus* | *Convolvulus farinosus* | rbcL | HM849910 | KC529208 | KC529210 |
| Convolvulaceae | *Convolvulus* | *Convolvulus sagittatus* | rbcL | KC529271 |  |  |
| Convolvulaceae | *Evolvulus* | *Evolvulus alsinoides* | trnL | KR737964 | KR738653 | KR738561 |
| Convolvulaceae | *Evolvulus* | *Evolvulus alsinoides* | rbcL | KR737505 | KR737409 | MH670080 |
| Convolvulaceae | *Ipomoea* | *Ipomoea cairica* | trnL | KR738692 | KR738517 | KR738278 |
| Convolvulaceae | *Ipomoea* | *Ipomoea cairica* | rbcL | MK285102 | MK285101 | KR737546 |
| Convolvulaceae | *Ipomoea* | *Ipomoea coptica* | rbcL | GENG1655-16.rbcL | PNG087-18.rbcL | PNG461-18.rbcL |
| Convolvulaceae | *Ipomoea* | *Ipomoea crassipes* | trnL | MZ461561 |  |  |
| Convolvulaceae | *Ipomoea* | *Ipomoea obscura* | trnL | AY101077 | KP236630 | KR737758 |
| Convolvulaceae | *Ipomoea* | *Ipomoea obscura* | rbcL | KR737466 | MH767515 | KR737034 |
| Convolvulaceae | *Ipomoea* | *Ipomoea plebeia* | rbcL | MT084545 | MT084546 | MT084547 |
| Convolvulaceae | *Ipomoea* | *Ipomoea sinensis* | trnL | KP236639 | KR738378 | KR738555 |
| Convolvulaceae | *Ipomoea* | *Ipomoea sinensis* | rbcL | KR737202 | KR737403 | KR737417 |
| Convolvulaceae | *Jacquemontia* | *Jacquemontia tamnifolia* | trnL | MK187114 | MK187113 |  |
| Convolvulaceae | *Jacquemontia* | *Jacquemontia tamnifolia* | rbcL | KY627481 | PNG089-18.rbcL | PNG156-18.rbcL |
| Convolvulaceae | *Merremia* | *Merremia kentrocaulos* | trnL | KP236668 |  |  |
| Convolvulaceae | *Merremia* | *Merremia palmata* | trnL | KP236674 |  |  |
| Convolvulaceae | *Xenostegia* | *Xenostegia tridentata* | trnL | MH825729 | MH825726 | MH825728 |
| Convolvulaceae | *Xenostegia* | *Xenostegia tridentata* | rbcL | MT053434 | MT053435 |  |
| Corbichonia-ceae | *Corbichonia* | *Corbichonia decumbens* | rbcL | FN824475 | KF724212 | MH014583 |
| Crassulaceae | *Cotyledon* | *Cotyledon barbeyi* | trnL | MH503217 | LN878839 |  |
| Cucurbitaceae | *Coccinia* | *Coccinia adoensis* | trnL | HQ608403 | HQ608396 |  |
| Cucurbitaceae | *Coccinia* | *Coccinia adoensis* | rbcL | HQ608499 | HQ608498 | HQ608509 |
| Cucurbitaceae | *Coccinia* | *Coccinia rehmannii* | trnL | DQ536799 | HQ625496 | HQ608423 |
| Cucurbitaceae | *Coccinia* | *Coccinia rehmannii* | rbcL | DQ535793 |  |  |
| Cucurbitaceae | *Coccinia* | *Coccinia sessilifolia* | rbcL | AY968520 |  |  |
| Cucurbitaceae | *Cucumis* | *Cucumis anguria* | trnL | DQ785869 | MK797191 | KY434441 |
| Cucurbitaceae | *Cucumis* | *Cucumis anguria* | rbcL | DQ785827 | KY434397 | KY627502 |
| Cucurbitaceae | *Cucumis* | *Cucumis hirsutus* | trnL | DQ536804 |  |  |
| Cucurbitaceae | *Cucumis* | *Cucumis hirsutus* | rbcL | DQ535799 |  |  |
| Cucurbitaceae | *Cucumis* | *Cucumis metuliferus* | trnL | DQ785876 | DQ785877 | DQ536805 |
| Cucurbitaceae | *Cucumis* | *Cucumis metuliferus* | rbcL | DQ535801 | DQ785834 | DQ785835 |
| Cucurbitaceae | *Cucumis* | *Cucumis zeyheri* | trnL | DQ536807 |  |  |
| Cucurbitaceae | *Cucumis* | *Cucumis zeyheri* | rbcL | DQ535803 |  |  |
| Cucurbitaceae | *Kedrostis* | *Kedrostis africana* | rbcL | AJ235782 |  |  |
| Cucurbitaceae | *Kedrostis* | *Kedrostis foetidissima* | trnL | MK261580 | MK261369 | MK261412 |
| Cucurbitaceae | *Lagenaria* | *Lagenaria sphaerica* | rbcL | GQ248627 | EF590539 |  |
| Cucurbitaceae | *Momordica* | *Momordica balsamina* | trnL | GQ163228 | GQ163230 | GQ163227 |
| Cucurbitaceae | *Momordica* | *Momordica balsamina* | rbcL | GENG1047-15.rbcL | GENG1228-15.rbcL | GENG911-15.rbcL |
| Cucurbitaceae | *Momordica* | *Momordica boivinii* | trnL | GQ163231 | GQ163232 | GQ163233 |
| Cucurbitaceae | *Momordica* | *Momordica charantia* | trnL | DQ501269 | GQ163246 | GQ163247 |
| Cucurbitaceae | *Momordica* | *Momordica charantia* | rbcL | DQ535760 | KT779314 | MF071479 |
| Cucurbitaceae | *Momordica* | *Momordica foetida* | trnL | GQ163276 | GQ163278 | GQ163279 |
| Cucurbitaceae | *Momordica* | *Momordica foetida* | rbcL | DQ535829 |  |  |
| Cucurbitaceae | *Zehneria* | *Zehneria scabra* | trnL | KY523393 | KY523392 |  |
| Cucurbitaceae | *Zehneria* | *Zehneria scabra* | rbcL | AM234970 |  |  |
| Cyperaceae | *Bulbostylis* | *Bulbostylis burchellii* | rbcL | AM999780 |  |  |
| Cyperaceae | *Bulbostylis* | *Bulbostylis hispidula* | trnL | MG844244 |  |  |
| Cyperaceae | *Bulbostylis* | *Bulbostylis hispidula* | rbcL | AM999781 |  |  |
| Cyperaceae | *Cyperus* | *Cyperus compressus* | trnL | MK797200 |  |  |
| Cyperaceae | *Cyperus* | *Cyperus compressus* | rbcL | MH658803 | KY627252 |  |
| Cyperaceae | *Cyperus* | *Cyperus cyperoides* | trnL | JX644800 | KR737822 | KR738588 |
| Cyperaceae | *Cyperus* | *Cyperus cyperoides* | rbcL | AB369947 | JX644655 | KR736614 |
| Cyperaceae | *Cyperus* | *Cyperus esculentus* | rbcL | HM849937 | KJ773425 | LK029902 |
| Cyperaceae | *Cyperus* | *Cyperus rotundus* | trnL | JX644807 |  |  |
| Cyperaceae | *Cyperus* | *Cyperus rotundus* | rbcL | KJ773433 | JX644667 |  |
| Cyperaceae | [*Cyperus*](https://www.google.com/search?sxsrf=ALeKk03N8LpAdLTw8-jhj-YfzPW5hsoZpg:1616004783728&q=Pogonarthria+squarrosa&sa=X&ved=2ahUKEwjWj9v49rfvAhXsSxUIHXNCC1EQ7xYoAHoECAMQMA) | [*Cyperus rubicundus*](https://www.google.com/search?sxsrf=ALeKk03N8LpAdLTw8-jhj-YfzPW5hsoZpg:1616004783728&q=Pogonarthria+squarrosa&sa=X&ved=2ahUKEwjWj9v49rfvAhXsSxUIHXNCC1EQ7xYoAHoECAMQMA) | rbcL | KR736751 | KR737027 | KR737116 |
| Cyperaceae | *Fimbristylis* | *Fimbristylis complanata* | trnL | JX644781 | MG844232 |  |
| Cyperaceae | *Fimbristylis* | *Fimbristylis complanata* | rbcL | JX644687 |  |  |
| Cyperaceae | *Rhynchospora* | *Rhynchospora brownii* | rbcL | DQ058353 |  |  |
| Cyperaceae | *Schoenoplectus* | *Schoenoplectus corymbosus* | trnL | EF178607 | KM462532 |  |
| Cyperaceae | *Schoenoplectus* | *Schoenoplectus corymbosus* | rbcL | EF178570 |  |  |
| Dioscoreaceae | *Dioscorea* | *Dioscorea cotinifolia* | trnL | KR070863 | KR070862 | KR070864 |
| Dioscoreaceae | *Dioscorea* | *Dioscorea cotinifolia* | rbcL | EF614219 | KR087034 | KR087035 |
| Ebenaceae | *Diospyros* | *Diospyros mespiliformis* | trnL | DQ924256 | FJ238259 |  |
| Ebenaceae | *Diospyros* | *Diospyros mespiliformis* | rbcL | EU980712 | JF265390 | KP996854 |
| Ebenaceae | *Euclea* | *Euclea crispa* | trnL | DQ924290 | FJ238241 |  |
| Ebenaceae | *Euclea* | *Euclea crispa* | rbcL | EU980789 | JX572574 |  |
| Ebenaceae | *Euclea* | *Euclea divinorum* | trnL | DQ924291 | KR737679 | KR737729 |
| Ebenaceae | *Euclea* | *Euclea divinorum* | rbcL | EU980790 | KR736572 | KR737313 |
| Ebenaceae | *Euclea* | *Euclea natalensis* | trnL | DQ924292 | FJ238243 |  |
| Ebenaceae | *Euclea* | *Euclea natalensis* | rbcL | EU980791 | JF265419 | JF265420 |
| Ebenaceae | *Euclea* | *Euclea schimperi* | trnL | FJ238246 |  |  |
| Ebenaceae | *Euclea* | *Euclea schimperi* | rbcL | JF265421 | JF265422 |  |
| Ebenaceae | *Euclea* | *Euclea undulata* | trnL | DQ924293 | FJ238248 |  |
| Ebenaceae | *Euclea* | *Euclea undulata* | rbcL | EU980792 | EU042179 | JF265423 |
| Ebenaceae | *Royena* | *Royena lycioides* | trnL | FJ238254 |  |  |
| Ebenaceae | *Royena* | *Royena lycioides* | rbcL | EU980802 | JF265389 | JX572516 |
| Ehretiaceae | *Cordia* | *Cordia caffra* | rbcL | JF265366 |  |  |
| Ehretiaceae | *Cordia* | *Cordia grandicalyx* | rbcL | JF265367 |  |  |
| Ehretiaceae | *Cordia* | *Cordia monoica* | trnL | KF158202 | KR737768 | KR738540 |
| Ehretiaceae | *Cordia* | *Cordia monoica* | rbcL | KF158112 | KR736555 | KR737206 |
| Ehretiaceae | *Ehretia* | *Ehretia amoena* | trnL | PNG303-18.trnL-F |  |  |
| Ehretiaceae | *Ehretia* | *Ehretia amoena* | rbcL | MN166723 | JX572543 | JF265404 |
| Ehretiaceae | *Ehretia* | *Ehretia rigida* | trnL | KF673278 |  |  |
| Ehretiaceae | *Ehretia* | *Ehretia rigida* | rbcL | EU042183 | JF265405 | JX572544 |
| Euphorbiaceae | Acalypha | *Acalypha indica* | rbcL | API024-12.rbcL | KF381097 | GENG676-14.rbcL |
| Euphorbiaceae | Acalypha | *Acalypha villicaulis* | rbcL | PNG352-18.rbcL |  |  |
| Euphorbiaceae | *Croton* | *Croton gratissimus* | trnL | AY794696 |  |  |
| Euphorbiaceae | *Croton* | *Croton gratissimus* | rbcL | EU213460 | EU213461 | EU213459 |
| Euphorbiaceae | *Croton* | *Croton megalobotrys* | trnL | KP878430 |  |  |
| Euphorbiaceae | *Croton* | *Croton megalobotrys* | rbcL | EU213463 | EU213464 | EU213462 |
| Euphorbiaceae | *Euphorbia* | *Chamaesyce (Euphorbia) inaequilatera* | trnL | KR738541 | KR737625 |  |
| Euphorbiaceae | *Euphorbia* | *Chamaesyce (Euphorbia) inaequilatera* | rbcL | KR737384 | KR737306 | KR737195 |
| Euphorbiaceae | *Euphorbia* | *Chamaesyce (Euphorbia) neopolycnemoides* | trnL | HQ645613 |  |  |
| Euphorbiaceae | *Euphorbia* | *Chamaesyce (Euphorbia) prostrata* | trnL | MH490421 | HQ645634 |  |
| Euphorbiaceae | *Euphorbia* | *Chamaesyce (Euphorbia) prostrata* | rbcL | MH767543 | HM849994 |  |
| Euphorbiaceae | *Euphorbia* | *Euphorbia cyathophora* | trnL | JN249622 |  |  |
| Euphorbiaceae | *Euphorbia* | *Euphorbia cyathophora* | rbcL | MH014775 |  |  |
| Euphorbiaceae | *Euphorbia* | *Euphorbia hirta* | trnL | MH490420 | HQ645584 | PNG215-18.trnL-F |
| Euphorbiaceae | *Euphorbia* | *Euphorbia indica* | trnL | HQ645653 | HQ645655 |  |
| Euphorbiaceae | *Euphorbia* | *Euphorbia indica* | rbcL | GENG1063-15.rbcL | GENG1256-15.rbcL |  |
| Euphorbiaceae | *Euphorbia* | *Euphorbia ingens* | trnL | JN207678 |  |  |
| Euphorbiaceae | *Euphorbia* | *Euphorbia tirucalli* | trnL | JN207723 | JN249733 | JN249734 |
| Euphorbiaceae | *Euphorbia* | *Euphorbia tirucalli* | rbcL | JQ952252 | JQ952253 | JQ952254 |
| Euphorbiaceae | *Jatropha* | *Jatropha zeyheri* | rbcL | JQ025058 |  |  |
| Euphorbiaceae | *Ricinus* | *Ricinus communis* | trnL | MK261654 | MK261515 | MK261385 |
| Euphorbiaceae | *Spirostachys* | *Spirostachys africana* | trnL | AY794621 |  |  |
| Euphorbiaceae | *Spirostachys* | *Spirostachys africana* | rbcL | AY794838 | AB267960 | JF265602 |
| Fabaceae | Abrus | *Abrus precatorius* | trnL | KX268134 | EF543423 | MK186948 |
| Fabaceae | Abrus | *Abrus precatorius* | rbcL | GU135185 | MN216761 | PNG357-18.rbcL |
| Fabaceae | Acacia | *Acacia senegal* | trnL | KX268139 | GQ872293 | AF522976 |
| Fabaceae | Acacia | *Acacia senegal* | rbcl | KNPA133-08.rbcLa | JX572227 | JF265258 |
| Fabaceae | Aeschynomene | *Aeschynomene indica* | rbcL | AF308701 | MN726221 | MN726220 |
| Fabaceae | *Albizia* | *Albizia anthelmintica* | trnL | JQ230209 |  |  |
| Fabaceae | *Albizia* | *Albizia anthelmintica* | rbcL | JF265275 | JX572254 |  |
| Fabaceae | *Albizia* | *Albizia forbesii* | rbcL | JF265277 | JX572255 |  |
| Fabaceae | *Albizia* | *Albizia harveyi* | trnL | EU439977 | PNG168-18.trnL-F |  |
| Fabaceae | *Albizia* | *Albizia harveyi* | rbcL | JX572257 | JF265278 | PNG168-18.rbcL |
| Fabaceae | *Albizia* | *Albizia petersiana* | trnL | JQ230213 | JQ230212 |  |
| Fabaceae | *Albizia* | *Albizia petersiana* | rbcL | JF265279 | JX572258 |  |
| Fabaceae | *Albizia* | *Albizia versicolor* | trnL | MK186969 | JQ230218 |  |
| Fabaceae | *Albizia* | *Albizia versicolor* | rbcL | JF265281 | JX572260 |  |
| Fabaceae | *Alysicarpus* | *Alysicarpus glumaceus* | trnL | KX268145 |  |  |
| Fabaceae | *Alysicarpus* | *Alysicarpus rugosus* | trnL | KX268147 |  |  |
| Fabaceae | *Alysicarpus* | *Alysicarpus rugosus* | rbcL | KX119259 |  |  |
| Fabaceae | *Bauhinia* | *Bauhinia galpinii* | trnL | PNG353-18.trnL-F | FJ801056 | FJ801055 |
| Fabaceae | *Bauhinia* | *Bauhinia galpinii* | rbcL | JF265301 | KNPA557-09.rbcLa | AM234262 |
| Fabaceae | *Bolusanthus* | *Bolusanthus speciosus* | rbcL | JF265305 |  |  |
| Fabaceae | *Cassia* | *Cassia abbreviata* | rbcL | JX572384 | JX572385 |  |
| Fabaceae | *Chamaecrista* | *Chamaecrista absus* | trnL | FJ009886 |  |  |
| Fabaceae | *Chamaecrista* | *Chamaecrista mimosoides* | trnL | MK187007 | KX268162 | PNG171-18.trnL-F |
| Fabaceae | *Chamaecrista* | *Chamaecrista mimosoides* | rbcL | GENG1014-15.rbcL | GENG592-14.rbcL |  |
| Fabaceae | *Colophospermum* | *Colophospermum mopane* | trnL | AF549260 |  |  |
| Fabaceae | *Colophospermum* | *Colophospermum mopane* | rbcL | JF265343 | JX572425 | KY988333 |
| Fabaceae | *Crotalaria* | *Crotalaria laburnifolia* | rbcL | JF265373 | JX120599 | JX120600 |
| Fabaceae | *Crotalaria* | *Crotalaria monteiroi* | rbcL | JQ041241 |  |  |
| Fabaceae | *Crotalaria* | *Crotalaria podocarpa* | rbcL | JQ067598 |  |  |
| Fabaceae | *Crotalaria* | *Crotalaria sp.* | trnL | KR737909 | KR738394 | KR738152 |
| Fabaceae | *Crotalaria* | *Crotalaria sphaerocarpa* | rbcL | JQ041269 | JQ067599 |  |
| Fabaceae | *Crotalaria* | *Crotalaria virgulata* | rbcL | JQ041275 |  |  |
| Fabaceae | *Dalbergia* | *Dalbergia melanoxylon* | rbcL | KM510271 | JF265384 | JX572502 |
| Fabaceae | *Dichrostachys* | *Dichrostachys cinerea* | trnL | KX268182 | EU439993 | PNG507-18.trnL-F |
| Fabaceae | *Dichrostachys* | *Dichrostachys cinerea* | rbcL | MN591166 | KX119290 | JF265387 |
| Fabaceae | *Dumasia* | *Dumasia villosa* | trnL | MK501220 | MK501221 | MK501222 |
| Fabaceae | *Dumasia* | *Dumasia villosa* | rbcL | EU717265 | MK501095 |  |
| Fabaceae | *Erythrina* | *Erythrina humeana* | rbcL | JF265413 | JX571824 | MK238900 |
| Fabaceae | *Erythrina* | *Erythrina latissima* | rbcL | JF265414 | MK238850 | MK238951 |
| Fabaceae | *Indigastrum* | *Indigastrum costatum* | rbcL | KNPA564-09.rbcLa |  |  |
| Fabaceae | *Indigofera* | *Indigofera arrecta* | trnL | KX268190 | MK261454 | MK261429 |
| Fabaceae | *Indigofera* | *Indigofera arrecta* | rbcL | KX119296 | KM894230 |  |
| Fabaceae | *Indigofera* | *Indigofera astragalina* | trnL | MK187106 | PNG041-18.trnL-F |  |
| Fabaceae | *Indigofera* | *Indigofera astragalina* | rbcL | GENG1258-15.rbcL | PNG041-18.rbcL | PNG074-18.rbcL |
| Fabaceae | *Indigofera* | *Indigofera lupatana* | rbcL | MH630254 | MN166680 |  |
| Fabaceae | *Indigofera* | *Indigofera rhytidocarpa* | rbcL | JX905964 |  |  |
| Fabaceae | *Indigofera* | *Indigofera schimperi* | trnL | MK261464 | MK261414 |  |
| Fabaceae | *Indigofera* | *Indigofera spicata* | trnL | MK187107 | PNG081-18.trnL-F |  |
| Fabaceae | *Indigofera* | *Indigofera spicata* | rbcL | KX119303 | KJ773588 | KY627231 |
| Fabaceae | *Indigofera* | *Indigofera tinctoria* | trnL | MK187108 | PNG482-18.trnL-F |  |
| Fabaceae | *Indigofera* | *Indigofera tinctoria* | rbcL | JF265485 | KJ939238 | KJ939239 |
| Fabaceae | *Macrotyloma* | *Macrotyloma uniflorum* | trnL | EU717341 |  |  |
| Fabaceae | *Macrotyloma* | *Macrotyloma uniflorum* | rbcL | EU717269 |  |  |
| Fabaceae | *Mundulea* | *Mundulea sericea* | rbcL | JF265522 | JQ025063 | JX572788 |
| Fabaceae | *Ormocarpum* | *Ormocarpum trichocarpum* | rbcL | JF265535 | JX572810 | KNPA542-09.rbcLa |
| Fabaceae | *Peltophorum* | *Peltophorum africanum* | trnL | AY899712 |  |  |
| Fabaceae | *Peltophorum* | *Peltophorum africanum* | rbcL | AY904399 | JF265546 | JX571871 |
| Fabaceae | *Philenoptera* | *Philenoptera violacea* | trnL | EU717357 | MK187167 | MK187168 |
| Fabaceae | *Philenoptera* | *Philenoptera violacea* | rbcL | JF265547 |  |  |
| Fabaceae | *Pterocarpus* | *Pterocarpus angolensis* | trnL | JN083646 |  |  |
| Fabaceae | *Pterocarpus* | *Pterocarpus angolensis* | rbcL | JN083717 | JN083716 | JF265564 |
| Fabaceae | *Pterocarpus* | *Pterocarpus rotundifolius* | trnL | JN083679 | JN083677 | JN083678 |
| Fabaceae | *Pterocarpus* | *Pterocarpus rotundifolius* | rbcL | JN083748 | JN083749 | JN083751 |
| Fabaceae | *Rhynchosia* | *Rhynchosia fleckii* | trnL | MT547522 |  |  |
| Fabaceae | *Rhynchosia* | *Rhynchosia fleckii* | rbcL | MZ461592 |  |  |
| Fabaceae | *Rhynchosia* | *Rhynchosia minima* | trnL | KR738160 | KR738354 | MT547495 |
| Fabaceae | *Rhynchosia* | *Rhynchosia minima* | rbcL | KR737567 | MH767691 | MH549966 |
| Fabaceae | *Rhynchosia* | *Rhynchosia monophylla* | rbcL | KF147510 |  |  |
| Fabaceae | *Schotia* | *Schotia brachypetala* | trnL | AY232752 | GQ405142 |  |
| Fabaceae | *Schotia* | *Schotia brachypetala* | rbcL | JF265583 | JQ025087 |  |
| Fabaceae | *Schotia* | *Schotia capitata* | trnL | GQ405167 |  |  |
| Fabaceae | *Schotia* | *Schotia capitata* | rbcL | JF265584 |  |  |
| Fabaceae | Senegalia | *Acacia (Senegalia) galpinii* | trnL | EU440000 | GQ872268 | KY688589 |
| Fabaceae | Senegalia | *Acacia (Senegalia) galpinii* | rbcL | JX572194 |  |  |
| Fabaceae | Senegalia | *Acacia (Senegalia) nigrescens* | trnL | KY688630 | KY688631 |  |
| Fabaceae | Senegalia | *Acacia (Senegalia) nigrescens* | rbcL | EU213438 | EU213439 | JX518103 |
| Fabaceae | Senegalia | *Acacia (Senegalia) schweinfurthii* | trnL | AF522979 | EU439976 | GQ872290 |
| Fabaceae | Senegalia | *Acacia (Senegalia) schweinfurthii* | rbcL | JF265257 | JX572225 |  |
| Fabaceae | Senegalia | *Acacia (Senegalia) welwitschii* | trnL | KY688668 | KY688667 | GQ872299 |
| Fabaceae | Senegalia | *Acacia (Senegalia) welwitschii* | rbcl | JF265262 | KNPA125-08.rbcLa | KNPA144-08.rbcLa |
| Fabaceae | Senegalia | *Acacia burkei* | trnL | GQ872260 |  |  |
| Fabaceae | Senegalia | *Acacia burkei* | rbcL | JX572186 |  |  |
| Fabaceae | Senegalia | *Acacia caffra* | trnL | GQ872261 | KY688585 |  |
| Fabaceae | Senegalia | *Acacia caffra* | rbcL | JX572187 | JF265246 |  |
| Fabaceae | Senegalia | *Acacia erubescens* | trnL | GQ872266 | KY688587 | KY688586 |
| Fabaceae | Senegalia | *Acacia erubescens* | rbcL | JF265248 |  |  |
| Fabaceae | *Senegalia* | *Senegalia mellifera* | trnL | KR737826 | KR738010 | KR738258 |
| Fabaceae | *Senegalia* | *Senegalia mellifera* | rbcL | JQ025017 | JX572211 | JX572212 |
| Fabaceae | *Senna* | *Senna bicapsularis* | rbcL | MF135382 | LC385911 | LC385914 |
| Fabaceae | *Senna* | *Senna didymobotrya* | trnL | MK261343 |  |  |
| Fabaceae | *Senna* | *Senna italica* | trnL | KX268211 |  |  |
| Fabaceae | *Senna* | *Senna italica* | rbcL | KY464112 | KY464113 | KY623359 |
| Fabaceae | *Senna* | *Senna obtusifolia* | trnL | KX268212 |  |  |
| Fabaceae | *Senna* | *Senna obtusifolia* | rbcL | KJ773878 | MF135419 | GQ436733 |
| Fabaceae | *Senna* | *Senna occidentalis* | trnL | KX268213 | AF365030 | EU361836 |
| Fabaceae | *Senna* | *Senna occidentalis* | rbcL | KJ773879 | MH767474 |  |
| Fabaceae | *Sesbania* | *Sesbania bispinosa* | trnL | KX268217 |  |  |
| Fabaceae | *Sesbania* | *Sesbania bispinosa* | rbcL | MH767693 |  |  |
| Fabaceae | *Stylosanthes* | *Stylosanthes fruticosa* | rbcL | KR736382 | KR736378 | KR736946 |
| Fabaceae | *Tephrosia* | *Tephrosia purpurea* | rbcL | MG946868 | MG946869 | MH767700 |
| Fabaceae | *Tephrosia* | *Tephrosia rhodesica* | rbcL | EU717288 |  |  |
| Fabaceae | *Teramnus* | *Teramnus labialis* | trnL | LC315106 | EU717329 | LC315120 |
| Fabaceae | *Teramnus* | *Teramnus labialis* | rbcL | API314-14.rbcL | GENG1146-15.rbcL | GENG1708-16.rbcL |
| Fabaceae | *Tylosema* | *Tylosema fassoglense* | trnL | FJ801091 | FJ801124 | EU361743 |
| Fabaceae | Vachellia | *Acacia (Vachellia) exuvialis* | trnL | GQ872267 |  |  |
| Fabaceae | Vachellia | *Acacia (Vachellia) exuvialis* | rbcL | EU213437 | EU213436 | EU213435 |
| Fabaceae | Vachellia | *Acacia (Vachellia) gerrardii* | trnL | KR738572 | KR738237 | KR738524 |
| Fabaceae | Vachellia | *Acacia (Vachellia) gerrardii* | rbcL | JF265250 | KR737061 | KR737364 |
| Fabaceae | Vachellia | *Acacia (Vachellia) grandicornuta* | trnL | EU440026 | GQ872271 |  |
| Fabaceae | Vachellia | *Acacia (Vachellia) grandicornuta* | rbcL | JF265251 | JX572197 |  |
| Fabaceae | Vachellia | *Acacia (Vachellia) karroo* | trnL | AF522972 | GQ872274 |  |
| Fabaceae | Vachellia | *Acacia (Vachellia) karroo* | rbcL | EU042178 | JX572203 | AM235003 |
| Fabaceae | Vachellia | *Acacia (Vachellia) luederitzii* | trnL | GQ872278 | JQ230220 | JQ230219 |
| Fabaceae | Vachellia | *Acacia (Vachellia) luederitzii* | rbcL | JX572208 | JX572207 | JF265253 |
| Fabaceae | Vachellia | *Acacia (Vachellia) nilotica* | trnL | PNG427-18.trnL-F | AF522973 | KR738665 |
| Fabaceae | Vachellia | *Acacia (Vachellia) nilotica* | rbcL | JF265255 | JX572217 | MH560452 |
| Fabaceae | Vachellia | *Acacia (Vachellia) tortilis* | trnL | HM020822 | AF522974 | KR738179 |
| Fabaceae | Vachellia | *Acacia (Vachellia) tortilis* | rbcL | JF265261 | JX572233 | MN592492 |
| Fabaceae | Vachellia | *Acacia (Vachellia) xanthophloea* | trnL | KR738566 | KR738442 | KR737864 |
| Fabaceae | Vachellia | *Acacia (Vachellia) xanthophloea* | rbcL | JF265263 | JX572235 | KR737272 |
| Fabaceae | Vachellia | *Acacia borleae* | rbcL | JF265243 | JX572185 |  |
| Fabaceae | Vachellia | *Acacia robusta* | trnL | MK186956 |  |  |
| Fabaceae | Vachellia | *Acacia robusta* | rbcL | JF265256 | JX572223 | PNG466-18.rbcL |
| Fabaceae | *Vigna* | *Vigna luteola* | rbcL | KJ773979 | MH550040 |  |
| Fabaceae | *Vigna* | *Vigna unguiculata* | trnL | AB304074 | GQ411715 | AB304023 |
| Fabaceae | *Vigna* | *Vigna unguiculata* | rbcL | EU717266 | GQ411659 | API148-12.rbcL |
| Fabaceae | *Vigna* | *Vigna vexillata* | trnL | AB304024 |  |  |
| Fabaceae | *Xanthocercis* | *Xanthocercis zambesiaca* | rbcL | JX573092 |  |  |
| Gentianaceae | *Enicostema* | *Enicostema axillare* | rbcL | API204-12.rbcL | GENG1589-16.rbcL | GENG790-14.rbcL |
| Geraniaceae | *Monsonia* | *Monsonia angustifolia* | trnL | KR737612 | KR737696 | KR737737 |
| Geraniaceae | *Monsonia* | *Monsonia angustifolia* | rbcL | KR737553 | KR736723 | KR737054 |
| Geraniaceae | *Monsonia* | *Monsonia emarginata* | trnL | HE795473 |  |  |
| Gisekiaceae | *Gisekia* | *Gisekia africana* | trnL | HE585086 |  |  |
| Heliotropiaceae | *Heliotropium* | *Heliotropium ovalifolium* | trnL | KY764559 | MK187085 | PNG267-18.trnL-F |
| Heliotropiaceae | *Heliotropium* | *Heliotropium ovalifolium* | rbcL | KU711591 | KU711592 | KU711590 |
| Heliotropiaceae | *Heliotropium* | *Heliotropium strigosum* | trnL | MK261620 |  |  |
| Heliotropiaceae | *Heliotropium* | *Heliotropium strigosum* | rbcL | MF694729 |  |  |
| Hypoxidaceae | *Hypoxis* | *Hypoxis hemerocallidea* | trnL | HM459507 |  |  |
| Kewaceae | *Kewa* | *Kewa bowkeriana* | trnL | MK261354 | MK261495 | MK261437 |
| Kewaceae | *Kewa* | *Kewa bowkeriana* | rbcL | FN824476 |  |  |
| Kewaceae | *Kewa* | *Kewa salsoloides* | trnL | KX197538 |  |  |
| Kewaceae | *Kewa* | *Kewa salsoloides* | rbcL | FN824477 | FN824478 |  |
| Kirkiaceae | *Kirkia* | *Kirkia wilmsii* | rbcL | JF265493 |  |  |
| Lamiaceae | Acrotome | *Acrotome hispida* | trnL | EU138376 | EU138299 |  |
| Lamiaceae | Acrotome | *Acrotome inflata* | trnL | EU138378 | EU138377 | EU138301 |
| Lamiaceae | *Clerodendrum* | *Clerodendrum glabrum* | rbcL | JF265341 | JX572414 |  |
| Lamiaceae | *Clerodendrum* | *Clerodendrum ternatum* | rbcL | KF147460 |  |  |
| Lamiaceae | *Leonotis* | *Leonotis nepetifolia* | trnL | EU138385 | EU138386 | KR737719 |
| Lamiaceae | *Leonotis* | *Leonotis nepetifolia* | rbcL | JF265501 | KR736498 | KR736355 |
| Lamiaceae | *Leonotis* | *Leonotis ocymifolia* | trnL | EU138388 | EU138389 | EU138387 |
| Lamiaceae | *Leucas* | *Leucas capensis* | trnL | EU138399 | EU138322 |  |
| Lamiaceae | *Leucas* | *Leucas glabrata* | trnL | MK261556 | EU138407 | EU138408 |
| Lamiaceae | *Leucas* | *Leucas martinicensis* | trnL | EU138416 | MK261661 | EU138339 |
| Lamiaceae | *Leucas* | *Leucas martinicensis* | rbcL | JQ673540 |  |  |
| Lamiaceae | *Leucas* | *Leucas neuflizeana* | trnL | EU138421 | MK261529 | EU138344 |
| Lamiaceae | *Leucas* | *Leucas sexdentata* | trnL | EU138424 | EU138347 |  |
| Lamiaceae | *Ocimum* | *Becium (Ocimum) filamentosum* | trnL | KR738704 | KR738267 | KR737770 |
| Lamiaceae | *Ocimum* | *Becium (Ocimum) filamentosum* | rbcL | KR737559 | KR737440 | KR737090 |
| Lamiaceae | *Ocimum* | *Ocimum americanum* | trnL | AJ505464 | MK261541 | MK261707 |
| Lamiaceae | *Ocimum* | *Ocimum americanum* | rbcL | MF468186 | MF468187 | MF468188 |
| Lamiaceae | *Ocimum* | *Ocimum gratissimum* | trnL | AJ505467 | AJ505468 | AJ505469 |
| Lamiaceae | *Ocimum* | *Ocimum gratissimum* | rbcL | MF468192 | MF468193 | MF468194.2 |
| Lamiaceae | *Ocimum* | *Ocimum labiatum* | trnL | AJ505470 | AJ505471 |  |
| Lamiaceae | *Ocimum* | *Ocimum labiatum* | trnL | EU543142 | EU543143 |  |
| Lamiaceae | *Plectranthus* | *Plectranthus caninus* | trnL | MK261509 | MK261511 |  |
| Lamiaceae | *Stachys* | *Stachys hyssopoides* | trnL | FJ854319 | FJ854218 |  |
| Lamiaceae | *Tetradenia* | *Tetradenia riparia* | trnL | MK261372 |  |  |
| Lamiaceae | *Tetradenia* | *Tetradenia riparia* | rbcL | MK285231 |  |  |
| Lamiales | Bignoniaceae | *Rhigozum zambesiacum* | rbcL | MZ461591 |  |  |
| Loganiaceae | *Strychnos* | *Strychnos madagascariensis* | trnL | MK187225 | MK187226 | KM365132 |
| Loganiaceae | *Strychnos* | *Strychnos madagascariensis* | rbcL | EU213525 | EU213524 | EU213523 |
| Loganiaceae | *Strychnos* | *Strychnos spinosa* | rbcL | EU213526 | EU213528 | EU213527 |
| Malpighiaceae | *Triaspis* | *Triaspis hypericoides* | rbcL | HQ247598 | HQ247596 | JF265632 |
| Malvaceae | Abutilon | *Abutilon angulatum* | trnL | PNG488-18.trnL-F |  |  |
| Malvaceae | Abutilon | *Abutilon angulatum* | rbcL | JX572177 | JF265241 | PNG020-18.rbcL |
| Malvaceae | Abutilon | *Abutilon austroafricanum* | rbcL | MZ461571 |  |  |
| Malvaceae | Abutilon | *Abutilon grandiflorum* | trnL | MK261506 |  |  |
| Malvaceae | Abutilon | *Abutilon mauritianum* | trnL | KR738146 | KR737889 | KR737749 |
| Malvaceae | Abutilon | *Abutilon mauritianum* | rbcL | KR737168 | KR737093 | KR736682 |
| Malvaceae | Abutilon | *Abutilon ramosum* | rbcL | GENG1244-15.rbcL | GENG480-14.rbcL |  |
| Malvaceae | Abutilon | *Abutilon sonneratianum* | rbcL | JX572178 |  |  |
| Malvaceae | *Cienfuegosia* | *Cienfuegosia hildebrandtii* | rbcL | PNG143-18.rbcL |  |  |
| Malvaceae | *Corchorus* | *Corchorus trilocularis* | trnL | MK261361 | MK261747 |  |
| Malvaceae | *Corchorus* | *Corchorus trilocularis* | rbcL | MK285157 |  |  |
| Malvaceae | *Dombeya* | *Dombeya rotundifolia* | rbcL | MH630256 | JF265396 | JQ025044 |
| Malvaceae | *Gossypium* | *Gossypium herbaceum* | rbcL | JF265447 | JN243187 | JN243188 |
| Malvaceae | *Grewia* | *Grewia bicolor* | trnL | MK261494 | MK261473 | MK261547 |
| Malvaceae | *Grewia* | *Grewia bicolor* | rbcL | EU213485 | EU213486 | MZ461582 |
| Malvaceae | *Grewia* | *Grewia flava* | rbcL | KM894221 |  |  |
| Malvaceae | *Grewia* | *Grewia flavescens* | trnL | MK187077 |  |  |
| Malvaceae | *Grewia* | *Grewia flavescens* | rbcL | EU213487 | EU213488 | JF265450 |
| Malvaceae | *Grewia* | *Grewia hexamita* | rbcL | JF265452 |  |  |
| Malvaceae | *Grewia* | *Grewia monticola* | trnL | MK187080 |  |  |
| Malvaceae | *Grewia* | *Grewia monticola* | rbcL | JF265454 | JX572641 |  |
| Malvaceae | *Grewia* | *Grewia villosa* | rbcL | EU213491 | EU213489 | EU213490 |
| Malvaceae | *Hermannia* | *Hermannia sp.* | rbcL | AM235025 |  |  |
| Malvaceae | *Hibiscus* | *Hibiscus calyphyllus* | trnL | KR737859 | KR737981 | KR738580 |
| Malvaceae | *Hibiscus* | *Hibiscus calyphyllus* | rbcL | KR736819 | KR737307 | KR737425 |
| Malvaceae | *Hibiscus* | *Hibiscus cannabinus* | trnL | MK187087 | JQ625353 | AY727227 |
| Malvaceae | *Hibiscus* | *Hibiscus cannabinus* | rbcL | DBHI004-16.rbcL | DBHI005-16.rbcL | DBHI006-16.rbcL |
| Malvaceae | *Hibiscus* | *Hibiscus lunariifolius* | trnL | KR738153 | KR737989 | KR738077 |
| Malvaceae | *Hibiscus* | *Hibiscus lunariifolius* | rbcL | KR736772 | KR736788 | KR736969 |
| Malvaceae | *Hibiscus* | *Hibiscus micranthus* | trnL | MK261364 | MK261519 | MK261642 |
| Malvaceae | *Hibiscus* | *Hibiscus micranthus* | rbcL | JF265474 | JX572675 | KR737285 |
| Malvaceae | *Hibiscus* | *Hibiscus physaloides* | trnL | PNG058-18.trnL-F |  |  |
| Malvaceae | *Hibiscus* | *Hibiscus physaloides* | rbcL | PNG058-18.rbcL |  |  |
| Malvaceae | *Hibiscus* | *Hibiscus pusillus* | rbcL | KBGPP016-18.rbcLa | KBGPP029-18.rbcLa | KBGPP056-18.rbcLa |
| Malvaceae | *Hibiscus* | *Hibiscus sabiensis* | rbcL | MZ461583 |  |  |
| Malvaceae | *Hibiscus* | *Hibiscus trionum* | trnL | KR737595 | KR737899 | KR738670 |
| Malvaceae | *Hibiscus* | *Hibiscus trionum* | rbcL | HM850051 | MH658267 | MG246509 |
| Malvaceae | *Hibiscus* | *Hibiscus vitifolius* | trnL | MK187091 |  |  |
| Malvaceae | *Hibiscus* | *Hibiscus vitifolius* | rbcL | PNG350-18.rbcL |  |  |
| Malvaceae | *Malvastrum* | *Malvastrum coromandelianum* | rbcL | MH050033 |  |  |
| Malvaceae | *Melhania* | *Melhania prostrata* | rbcL | MZ461587 |  |  |
| Malvaceae | *Pavonia* | *Pavonia burchellii* | rbcL | KNPA551-09.rbcLa |  |  |
| Malvaceae | *Sida* | *Sida alba* | trnL | MK261545 | MK261526 |  |
| Malvaceae | *Sida* | *Sida cordifolia* | trnL | MK187213 | MK187212 |  |
| Malvaceae | *Sida* | *Sida cordifolia* | rbcL | KJ773888 | MH767640 |  |
| Malvaceae | *Sida* | *Sida ovata* | trnL | KR737694 | KR738415 | KR738638 |
| Malvaceae | *Sida* | *Sida ovata* | rbcL | KR737490 | KR737242 | KR736471 |
| Malvaceae | *Sida* | *Sida rhombifolia* | rbcL | HM850353 | KJ773889 |  |
| Malvaceae | *Sida* | *Sida spinosa* | trnL | MK261545 | MK261526 |  |
| Malvaceae | *Sida* | *Sida spinosa* | rbcL | DQ006105 | MG245969 | MG247242 |
| Malvaceae | *Triumfetta* | *Triumfetta annua* | rbcL | JQ933514 |  |  |
| Malvaceae | *Triumfetta* | *Triumfetta pentandra* | rbcL | GENG1254-15.rbcL |  |  |
| Malvaceae | *Triumfetta* | *Triumfetta rhomboidea* | rbcL | KX527080 | GENG1186-15.rbcL |  |
| Malvaceae | *Waltheria* | *Waltheria indica* | trnL | MK797729 | KR738059 | KR737724 |
| Malvaceae | *Waltheria* | *Waltheria indica* | rbcL | KR736865 | MH767737 | MF135410 |
| Meliaceae | *Ekebergia* | *Ekebergia capensis* | trnL | KU939116 |  |  |
| Meliaceae | *Ekebergia* | *Ekebergia capensis* | rbcL | AJ402947 | AY128228 | KM894215 |
| Meliaceae | *Melia* | *Melia azedarach* | trnL | FM179536 | FN599481 | AB057535 |
| Meliaceae | *Melia* | *Melia azedarach* | rbcL | MG946851 | AY128234 | FN599453 |
| Meliaceae | *Trichilia* | *Trichilia emetica* | trnL | MK187234 | KU939125 |  |
| Meliaceae | *Trichilia* | *Trichilia emetica* | rbcL | U39082.2 | AY128244 | JX307354 |
| Meliaceae | *Turraea* | *Turraea obtusifolia* | rbcL | JF265641 |  |  |
| Menisperma-ceae | *Cissampelos* | *Cissampelos mucronata* | rbcL | PNG372-18.rbcL |  |  |
| Menisperma-ceae | *Cocculus* | *Cocculus hirsutus* | trnL | MT153833 |  |  |
| Menisperma-ceae | *Cocculus* | *Cocculus hirsutus* | rbcL | MT153748 | MT153750 | MK482693 |
| Molluginaceae | *Glinus* | *Glinus oppositifolius* | trnL | MK187070 | MK187071 |  |
| Molluginaceae | *Glinus* | *Glinus oppositifolius* | rbcL | FN824414 | FN824415 | FN824416 |
| Molluginaceae | *Mollugo* | *Mollugo nudicaulis* | rbcL | FN824444 | FN824445 | FN824446 |
| Moraceae | *Ficus* | *Ficus abutilifolia* | rbcL | EU213477 | EU213475 | EU213476 |
| Moraceae | *Ficus* | *Ficus capreifolia* | rbcL | JX572601 |  |  |
| Moraceae | *Ficus* | *Ficus ingens* | trnL | MK261593 |  |  |
| Moraceae | *Ficus* | *Ficus ingens* | rbcL | MK285222 |  |  |
| Moraceae | *Ficus* | *Ficus salicifolia* | rbcL | JF265436 | JX572609 |  |
| Moraceae | *Ficus* | *Ficus sur* | trnL | GQ504506 |  |  |
| Moraceae | *Ficus* | *Ficus sur* | rbcL | JF265438 |  |  |
| Moraceae | *Ficus* | *Ficus sycomorus* | trnL | MK261538 | MK261530 | GQ504508 |
| Moraceae | *Ficus* | *Ficus sycomorus* | rbcL | EU213481 | EU213482 | EU213483 |
| Moraceae | *Ficus* | *Ficus thonningii* | trnL | GQ504484 | GQ504493 |  |
| Moraceae | *Ficus* | *Ficus thonningii* | rbcL | JF265432 | JX571841 |  |
| Myrtaceae | *Heteropyxis* | *Heteropyxis natalensis* | rbcL | AM235662 | JF265471 | JQ025054 |
| Myrtaceae | *Psidium* | *Psidium guajava* | trnL | MN443100 | KC428590 |  |
| Myrtaceae | *Psidium* | *Psidium guajava* | rbcL | KX527097 | KX021127 | MH069835 |
| Myrtaceae | *Syzygium* | *Syzygium guineense* | rbcL | JX573018 | JX573019 | JX573020 |
| Nyctaginaceae | *Boerhavia* | *Boerhavia diffusa* | trnL | KU172536 |  |  |
| Ochnaceae | *Ochna* | *Ochna inermis* | rbcL | JF265529 |  |  |
| Ochnaceae | *Ochna* | *Ochna natalitia* | rbcL | JF265530 | KF147495 | KF263366 |
| Oleaceae | *Jasminum* | *Jasminum fluminense* | trnL | KR738052 | KR738347 | KR738188 |
| Oleaceae | *Jasminum* | *Jasminum fluminense* | rbcL | GU135177 | MH549891 | JF265486 |
| Oleaceae | *Jasminum* | *Jasminum stenolobum* | rbcL | JF265488 | JX572699 |  |
| Onagraceae | *Ludwigia* | *Ludwigia adscendens* | trnL | KU941993 |  |  |
| Onagraceae | *Ludwigia* | *Ludwigia adscendens* | rbcL | KX527470 | PNG070-18.rbcL |  |
| Onagraceae | *Ludwigia* | *Ludwigia octovalvis* | trnL | KX168176 | KX168172 | KX168173 |
| Onagraceae | *Ludwigia* | *Ludwigia octovalvis* | rbcL | MH050043 | JF265505 |  |
| Onagraceae | *Ludwigia* | *Ludwigia stolonifera* | trnL | KR738124 | KR737926 |  |
| Onagraceae | *Ludwigia* | *Ludwigia stolonifera* | rbcL | KR736936 | KR736719 |  |
| Orchidaceae | *Ansellia* | *Ansellia africana* | rbcL | EU213444 | EU213445 | MH748844 |
| Orobanchaceae | *Striga* | *Striga bilabiata* | trnL | EU256642 |  |  |
| Orobanchaceae | *Striga* | *Striga elegans* | rbcL | EU213519 |  |  |
| Orobanchaceae | *Striga* | *Striga gesnerioides* | trnL | EU264195 |  |  |
| Orobanchaceae | *Striga* | *Striga gesnerioides* | rbcL | PNG110-18.rbcL |  |  |
| Oxalidaceae | *Oxalis* | *Oxalis latifolia* | trnL | JN639556 | JN639573 | JN639574 |
| Oxalidaceae | *Oxalis* | *Oxalis latifolia* | rbcL | EU002282 | HM850223 |  |
| Papaveraceae | *Argemone* | *Argemone mexicana* | trnL | PNG151-18.trnL-F | AY328248 |  |
| Papaveraceae | *Argemone* | *Argemone mexicana* | rbcL | GQ436730 | GENG647-14.rbcL | MG249174 |
| Passifloraceae | Adenia | *Adenia digitata* | trnL | MK186963 |  |  |
| Passifloraceae | *Tricliceras* | *Tricliceras laceratum* | rbcL | AB536573 |  |  |
| Pedaliaceae | *Ceratotheca* | *Ceratotheca triloba* | trnL | KJ743178 | AF482611 |  |
| Pedaliaceae | *Ceratotheca* | *Ceratotheca triloba* | rbcL | JQ025031 |  |  |
| Pedaliaceae | *Dicerocaryum* | *Dicerocaryum eriocarpum* | trnL | KJ743186 |  |  |
| Pedaliaceae | *Dicerocaryum* | *Dicerocaryum senecioides* | rbcL | PNG392-18.rbcL |  |  |
| Pedaliaceae | *Harpagophytum* | *Harpagophytum zeyheri* | trnL | KJ743192 |  |  |
| Pedaliaceae | *Harpagophytum* | *Harpagophytum zeyheri* | rbcL | KT717180 | KT717179 | KT717181 |
| Pedaliaceae | *Holubia* | *Holubia saccata* | trnL | KJ743197 |  |  |
| Pedaliaceae | *Sesamum* | *Sesamum alatum* | trnL | KJ743201 |  |  |
| Pedaliaceae | *Sesamum* | *Sesamum triphyllum* | trnL | KJ743202 |  |  |
| Peraceae | *Clutia* | *Clutia pulchella* | rbcL | JX572416 | AM234976 |  |
| Phyllanthaceae | *Bridelia* | *Bridelia cathartica* | trnL | MK186997 | FJ440011 |  |
| Phyllanthaceae | *Bridelia* | *Bridelia cathartica* | rbcL | JF265314 | JX572350 |  |
| Phyllanthaceae | *Bridelia* | *Bridelia mollis* | trnL | FJ440006 |  |  |
| Phyllanthaceae | *Bridelia* | *Bridelia mollis* | rbcL | JX572351 | KNPA342-09.rbcLa | JF265316 |
| Phyllanthaceae | *Flueggea* | *Flueggea virosa* | trnL | MK187066 |  |  |
| Phyllanthaceae | *Flueggea* | *Flueggea virosa* | rbcL | AY663585 | GQ436328 | MZ461581 |
| Phyllanthaceae | *Phyllanthus* | *Phyllanthus asperulatus* | rbcL | MZ461588 |  |  |
| Phyllanthaceae | *Phyllanthus* | *Phyllanthus incurvus* | trnL | MZ461567 |  |  |
| Phyllanthaceae | *Phyllanthus* | *Phyllanthus incurvus* | rbcL | MZ461589 |  |  |
| Phyllanthaceae | *Phyllanthus* | *Phyllanthus maderaspatensis* | trnL | MK261613 |  |  |
| Phyllanthaceae | *Phyllanthus* | *Phyllanthus maderaspatensis* | rbcL | MK244691 | GENG858-14.rbcL |  |
| Phyllanthaceae | *Phyllanthus* | *Phyllanthus nummulariifolius* | rbcL | AY663609 |  |  |
| Phyllanthaceae | *Phyllanthus* | *Phyllanthus reticulatus* | trnL | MK187174 |  |  |
| Phyllanthaceae | Thecacoris | *Acalypha glabrata* | rbcL | JF265264 | JX572237 | JX572238 |
| Piperaceae | *Peperomia* | *Peperomia blanda* | trnL | EU519741 |  |  |
| Piperaceae | *Peperomia* | *Peperomia blanda* | rbcL | KM895679 |  |  |
| Plantaginaceae | *Bacopa* | *Bacopa floribunda* | rbcL | LC214993 |  |  |
| Plumbagina-ceae | *Plumbago* | *Plumbago zeylanica* | trnL | AJ312248 | MH561153 |  |
| Plumbagina-ceae | *Plumbago* | *Plumbago zeylanica* | rbcL | MG946923 | MH582834 | LC637763 |
| Poaceae | *Andropogon* | *Andropogon chinensis* | rbcL | KNPA380-09.rbcLa |  |  |
| Poaceae | *Andropogon* | *Andropogon gayanus* | rbcL | JQ593287 | JQ593288 | MF998308 |
| Poaceae | *Aristida* | *Aristida adscensionis* | trnL | KR737828 | GQ924358 | GQ924356 |
| Poaceae | *Aristida* | *Aristida adscensionis* | rbcL | EF423002 | AM849349 | HE573453 |
| Poaceae | *Aristida* | *Aristida congesta* | trnL | KR738288 | GQ924369 | GQ924368 |
| Poaceae | *Aristida* | *Aristida congesta* | rbcL | FR821334 | KR737225 | KR737111 |
| Poaceae | *Aristida* | *Aristida meridionalis* | trnL | GQ924405 | GQ924295 |  |
| Poaceae | *Aristida* | *Aristida meridionalis* | rbcL | KNPA217-09.rbcLa | KNPA255-09.rbcLa |  |
| Poaceae | *Aristida* | *Aristida mollissima* | rbcL | HG970259 |  |  |
| Poaceae | *Aristida* | *Aristida scabrivalvis* | rbcL | KNPA259-09.rbcLa | KNPA275-09.rbcLa | KNPA320-09.rbcLa |
| Poaceae | *Aristida* | *Aristida stipitata* | trnL | GQ924418 | GQ924417 |  |
| Poaceae | *Aristida* | *Aristida stipitata* | rbcL | KNPA236-09.rbcLa | MK570614 |  |
| Poaceae | *Aristida* | *Aristida vestita* | trnL | GQ924425 | GQ924424 |  |
| Poaceae | *Aristida* | *Aristida vestita* | rbcL | HG970265 |  |  |
| Poaceae | *Bothriochloa* | *Bothriochloa bladhii* | trnL | DQ004960 |  |  |
| Poaceae | *Bothriochloa* | *Bothriochloa bladhii* | rbcL | MF998321 |  |  |
| Poaceae | *Bothriochloa* | *Bothriochloa insculpta* | trnL | KR738475 | KR738158 | KR737713 |
| Poaceae | *Bothriochloa* | *Bothriochloa insculpta* | rbcL | KNPA315-09.rbcLa | LN907891 | KNPA456-09.rbcLa |
| Poaceae | *Bothriochloa* | *Bothriochloa radicans* | rbcL | KNPA307-09.rbcLa | KNPA426-09.rbcLa | KNPA431-09.rbcLa |
| Poaceae | *Brachiaria* | *Brachiaria (Moorochloa) eruciformis* | trnL | GU594550 | KR738351 | KR738047 |
| Poaceae | *Brachiaria* | *Brachiaria (Moorochloa) eruciformis* | rbcL | KNPA428-09.rbcLa | KR736853 | KR736671 |
| Poaceae | *Brachiaria* | *Brachiaria (Urochloa) nigropedata* | trnL | GU594556 |  |  |
| Poaceae | *Brachiaria* | *Brachiaria (Urochloa) xantholeuca* | trnL | MK261501 |  |  |
| Poaceae | *Brachiaria* | *Brachiaria (Urochloa) xantholeuca* | rbcL | HE575847 | MF998173 |  |
| Poaceae | *Brachiaria* | *Brachiaria deflexa* | trnL | KR738230 | GU594571 | KR738079 |
| Poaceae | *Brachiaria* | *Brachiaria deflexa* | rbcL | KNPA216-09.rbcLa | KNPA280-09.rbcLa | MF998175 |
| Poaceae | *Brachiaria* | *Brachiaria serrata* | trnL | GU594558 |  |  |
| Poaceae | *Brachiaria* | *Brachiaria serrata* | rbcL | KNPA230-09.rbcLa | MF998176 | KJ740990 |
| Poaceae | *Cenchrus* | *Cenchrus ciliaris* | trnL | EU940006 | MK261388 | AF499168 |
| Poaceae | *Cenchrus* | *Cenchrus ciliaris* | rbcL | LN907904 | IPSUB369-18.rbcL | KNPA197-09.rbcLa |
| Poaceae | *Chloris* | *Chloris (Tetrapogon) roxburghiana* | trnL | MK261499 | MK261500 | MK261607 |
| Poaceae | *Chloris* | *Chloris (Tetrapogon) roxburghiana* | rbcL | KNPA195-09.rbcLa | KNPA196-09.rbcLa | MF998368 |
| Poaceae | *Chloris* | *Chloris gayana* | trnL | KR738355 | MK261700 | KR738264 |
| Poaceae | *Chloris* | *Chloris gayana* | rbcL | MF998383 | AM849409 | KR737178 |
| Poaceae | *Chloris* | *Chloris pycnothrix* | trnL | KR737740 | KR737623 | KR738569 |
| Poaceae | *Chloris* | *Chloris pycnothrix* | rbcL | KR736522 | KR736398 | KR737415 |
| Poaceae | *Chloris* | *Chloris virgata* | trnL | EF137560 | DQ655855 | DQ655856 |
| Poaceae | *Chloris* | *Chloris virgata* | rbcL | KNPA254-09.rbcLa | KNPA305-09.rbcLa | HE575821 |
| Poaceae | *Cymbopogon* | *Cymbopogon excavatus (caesius)* | rbcL | LN907911 | KJ740991 |  |
| Poaceae | *Cymbopogon* | *Cymbopogon plurinodis (pospischilii)* | rbcL | KNPA251-09.rbcLa | KNPA314-09.rbcLa |  |
| Poaceae | *Cynodon* | *Cynodon dactylon* | trnL | EF156685 | DQ655854 | MG709452 |
| Poaceae | *Cynodon* | *Cynodon dactylon* | rbcL | MF998366 | AM849393 |  |
| Poaceae | *Dactyloctenium* | *Dactyloctenium aegyptium* | trnL | EF156686 | DQ655864 | PNG506-18.trnL-F |
| Poaceae | *Dactyloctenium* | *Dactyloctenium aegyptium* | rbcL | EF125106 | AM887877 | MK285146 |
| Poaceae | *Dactyloctenium* | *Dactyloctenium giganteum* | rbcL | KNPA289-09.rbcLa |  |  |
| Poaceae | *Dichanthium* | *Dichanthium annulatum* | trnL | DQ004973 | DQ004975 | DQ004974 |
| Poaceae | *Dichanthium* | *Dichanthium annulatum* | rbcL | MF998322 | MK166796 | KX282694 |
| Poaceae | *Digitaria* | *Digitaria debilis* | rbcL | MF998283 |  |  |
| Poaceae | *Digitaria* | *Digitaria ternata* | trnL | MK261450 | MK261653 | MK261631 |
| Poaceae | *Digitaria* | *Digitaria ternata* | rbcL | MF998284 |  |  |
| Poaceae | *Digitaria* | *Digitaria velutina* | trnL | KR737704 | KR737800 | KR738531 |
| Poaceae | *Digitaria* | *Digitaria velutina* | rbcL | LN907922 | KR736483 | KR737373 |
| Poaceae | *Diheteropogon* | *Diheteropogon amplectens* | trnL | LN907923 | FN870803 |  |
| Poaceae | *Diplachne* | *Diplachne fusca* | trnL | EF156708 | DQ655862 |  |
| Poaceae | *Diplachne* | *Diplachne fusca* | rbcL | HE575827 | KP056888 | KP056889 |
| Poaceae | *Echinochloa* | *Echinochloa colona (crus-galli)* | trnL | KP879103 | MK797237 | KF163575 |
| Poaceae | *Echinochloa* | *Echinochloa colona (crus-galli)* | rbcL | HE573377 | KT365286 | KT365287 |
| Poaceae | *Elionurus* | *Elionurus muticus* | rbcL | MF998314 | KC123358 | FN870812 |
| Poaceae | *Enneapogon* | *Enneapogon cenchroides* | trnL | DQ655894 | KR738224 | KR737620 |
| Poaceae | *Enneapogon* | *Enneapogon cenchroides* | rbcL | MF998418 | LN907929 | KR737512 |
| Poaceae | *Enneapogon* | *Enneapogon scoparius* | trnL | DQ655895 | GU990367 | DQ655893 |
| Poaceae | *Enteropogon* | *Enteropogon macrostachyus* | trnL | DQ655863 | KR737769 | KR738020 |
| Poaceae | *Enteropogon* | *Enteropogon macrostachyus* | rbcL | KR736556 | KR736600 | KR737271 |
| Poaceae | *Eragrostis* | *Eragrostis aspera* | trnL | GU990374 |  |  |
| Poaceae | *Eragrostis* | *Eragrostis aspera* | rbcL | MF998415 |  |  |
| Poaceae | *Eragrostis* | *Eragrostis biflora* | trnL | DQ655879 |  |  |
| Poaceae | *Eragrostis* | *Eragrostis capensis* | trnL | DQ655881 | DQ655882 |  |
| Poaceae | *Eragrostis* | *Eragrostis capensis* | rbcL | MF998405 |  |  |
| Poaceae | *Eragrostis* | *Eragrostis chloromelas* | trnL | DQ655876 | DQ655877 |  |
| Poaceae | *Eragrostis* | *Eragrostis cilianensis* | trnL | GU990381 | KP711154 | MK261443 |
| Poaceae | *Eragrostis* | *Eragrostis cilianensis* | rbcL | MF596765 | MG228051 | MG228233 |
| Poaceae | *Eragrostis* | *Eragrostis cylindriflora* | trnL | MK261404 |  |  |
| Poaceae | *Eragrostis* | *Eragrostis cylindriflora* | rbcL | KNPA239-09.rbcLa | KNPA313-09.rbcLa | KNPA318-09.rbcLa |
| Poaceae | *Eragrostis* | *Eragrostis gummiflua* | trnL | KNPA270-09.rbcLa |  |  |
| Poaceae | *Eragrostis* | *Eragrostis heteromera* | trnL | GU990393 | MK261562 | MK261539 |
| Poaceae | *Eragrostis* | *Eragrostis lehmanniana* | trnL | DQ655883 | DQ655875 | GU990398 |
| Poaceae | *Eragrostis* | *Eragrostis lehmanniana* | rbcL | KNPA281-09.rbcLa | KNPA298-09.rbcLa | KNPA432-09.rbcLa |
| Poaceae | *Eragrostis* | *Eragrostis racemosa* | trnL | DQ655878 | KR738281 | KR738663 |
| Poaceae | *Eragrostis* | *Eragrostis racemosa* | rbcL | LN907940 | KR737517 | KR737105 |
| Poaceae | *Eragrostis* | *Eragrostis rigidior* | trnL | MK261336 | MK261448 | MK261716 |
| Poaceae | *Eragrostis* | *Eragrostis rotifer* | rbcL | KNPA283-09.rbcLa |  |  |
| Poaceae | *Eragrostis* | *Eragrostis superba* | trnL | DQ655880 | MG709444 | KR737798 |
| Poaceae | *Eragrostis* | *Eragrostis superba* | rbcL | KR736588 | KR736685 |  |
| Poaceae | *Eragrostis* | *Eragrostis trichophora* | trnL | GU990386 |  |  |
| Poaceae | *Eragrostis* | *Eragrostis trichophora* | rbcL | KNPA250-09.rbcLa | KNPA383-09.rbcLa | KNPA383-09.rbcLa |
| Poaceae | *Eriochloa* | *Eriochloa meyeriana* | trnL | GU594583 |  |  |
| Poaceae | *Eustachys* | *Eustachys paspaloides* | trnL | DQ655857 | MK261614 |  |
| Poaceae | *Eustachys* | *Eustachys paspaloides* | rbcL | AM235058 | KNPA206-09.rbcLa | KNPA228-09.rbcLa |
| Poaceae | *Fingerhuthia* | *Fingerhuthia africana* | trnL | EF156701 | DQ655891 |  |
| Poaceae | *Fingerhuthia* | *Fingerhuthia africana* | rbcL | HE575825 | AM235059 | KNPA442-09.rbcLa |
| Poaceae | *Hemarthria* | *Hemarthria altissima* | rbcL | FN870839 | GU135209 |  |
| Poaceae | *Heteropogon* | *Heteropogon contortus* | trnL | KY991322 | DQ004984 | KR737831 |
| Poaceae | *Heteropogon* | *Heteropogon contortus* | rbcL | HE575844 | AM235061 | KR737296 |
| Poaceae | *Hyparrhenia* | *Hyparrhenia filipendula* | trnL | KR737880 | KR737709 | KR738081 |
| Poaceae | *Hyparrhenia* | *Hyparrhenia filipendula* | rbcL | KR736673 | KR736488 | KR736888 |
| Poaceae | *Hyperthelia* | *Hyperthelia dissoluta* | trnL | DQ004985 |  |  |
| Poaceae | *Hyperthelia* | *Hyperthelia dissoluta* | rbcL | MF998325 | KNPA191-09.rbcLa | KNPA261-09.rbcLa |
| Poaceae | *Megathyrsus* | *Megathyrsus maximus* | trnL | GU594547 | KR738050 | KR738423 |
| Poaceae | *Megathyrsus* | *Megathyrsus maximus* | rbcL | LN907967 | FR667682 | MH050060 |
| Poaceae | *Melinis* | *Melinis nerviglumis* | rbcL | KJ740994 |  |  |
| Poaceae | *Melinis* | *Melinis repens* | trnL | GU594561 | KP879139 | KR737705 |
| Poaceae | *Melinis* | *Melinis repens* | rbcL | EF125136 | HE573397 | AM235064 |
| Poaceae | *Microchloa* | *Microchloa caffra* | rbcL | EF125102 | KNPA200-09.rbcLa |  |
| Poaceae | *Oropetium* | *Oropetium capense* | rbcL | HE575831 | FN870887 |  |
| Poaceae | *Panicum* | *Panicum coloratum* | trnL | PNG449-18.trnL-F |  |  |
| Poaceae | *Panicum* | *Panicum coloratum* | rbcL | AM849415 | KNPA282-09.rbcLa | KNPA310-09.rbcLa |
| Poaceae | *Panicum* | *Panicum deustum* | trnL | GU594591 | MK261732 | MK261752 |
| Poaceae | *Panicum* | *Panicum deustum* | rbcL | KNPA223-09.rbcLa | KNPA267-09.rbcLa |  |
| Poaceae | *Panicum* | *Panicum infestum* | rbcL | MF998177 |  |  |
| Poaceae | *Panicum* | *Panicum maximum* | trnL | KR738467 | KR738050 | KR738423 |
| Poaceae | *Panicum* | *Panicum maximum* | rbcL | KR737299 | KR736856 | KR736953 |
| Poaceae | *Paspalum* | *Paspalum distichum* | trnL | EU871100 | KX372461 |  |
| Poaceae | *Paspalum* | *Paspalum distichum* | rbcL | FN870399 | KC164328 | KF163532 |
| Poaceae | *Paspalum* | *Paspalum scrobiculatum* | rbcL | LN907994 | API322-14.rbcL |  |
| Poaceae | *Perotis* | *Perotis patens* | trnL | DQ655869 |  |  |
| Poaceae | *Perotis* | *Perotis patens* | rbcL | MF998375 | MF998377 | FR821340 |
| Poaceae | *Phragmites* | *Phragmites australis* | trnL | KC989616 | EF137518 | KX372469 |
| Poaceae | *Phragmites* | *Phragmites australis* | rbcL | EF423005 | KP056891 | MF065283 |
| Poaceae | *Phragmites* | *Phragmites mauritianus* | rbcL | LN908033 | PNG153-18.rbcL |  |
| Poaceae | *Pogonarthria* | *Pogonarthria squarrosa* | trnL | EF156724 | DQ655885 | DQ655884 |
| Poaceae | *Pogonarthria* | *Pogonarthria squarrosa* | rbcL | MF998403 | JN681701 | KNPA215-09.rbcLa |
| Poaceae | *Schmidtia* | *Schmidtia pappophoroides* | trnL | GU990368 | DQ655896 |  |
| Poaceae | *Schmidtia* | *Schmidtia pappophoroides* | rbcL | KNPA214-09.rbcLa | KNPA257-09.rbcLa | KNPA271-09.rbcLa |
| Poaceae | *Setaria* | *Setaria incrassata* | rbcL | LN908008 | KNPA233-09.rbcLa | KNPA265-09.rbcLa |
| Poaceae | *Setaria* | *Setaria pumila* | trnL | KX372487 | MK261548 | MK261576 |
| Poaceae | *Setaria* | *Setaria pumila* | rbcL | KC164338 | KF163543 | KJ841567 |
| Poaceae | *Setaria* | *Setaria sagittifolia* | trnL | PNG430-18.trnL-F |  |  |
| Poaceae | *Setaria* | *Setaria sagittifolia* | rbcL | MF998260 | KNPA222-09.rbcLa | KNPA296-09.rbcLa |
| Poaceae | *Setaria* | *Setaria sphacelata* | trnL | GU561494 | KR737631 | KR737695 |
| Poaceae | *Setaria* | *Setaria sphacelata* | rbcL | HE577880 | LN908011 | KR736472 |
| Poaceae | *Setaria* | *Setaria verticillata* | trnL | KX372489 | AF499154 | KR738477 |
| Poaceae | *Setaria* | *Setaria verticillata* | rbcL | LN908012 | FR821349 | HQ600366 |
| Poaceae | *Sorghum* | *Sorghum bicolor* | trnL | KX372491 | GQ867663 |  |
| Poaceae | *Sorghum* | *Sorghum bicolor* | rbcL | MG227513 | FSSM057-14.rbcLa | SDH2517-14.rbcLa |
| Poaceae | *Sorghum* | *Sorghum versicolor* | rbcL | MG772772 | KNPA234-09.rbcLa | KNPA404-09.rbcLa |
| Poaceae | *Sporobolus* | *Sporobolus africanus* | trnL | DQ655899 | KR737946 | KR738650 |
| Poaceae | *Sporobolus* | *Sporobolus africanus* | rbcL | HM850383 | KR736386 | KR737502 |
| Poaceae | *Sporobolus* | *Sporobolus festivus* | trnL | KR738150 | KR738123 | MK261557 |
| Poaceae | *Sporobolus* | *Sporobolus festivus* | rbcL | AM849383 | MF998397 | KR736966 |
| Poaceae | *Sporobolus* | *Sporobolus fimbriatus* | trnL | DQ655901 | DQ655903 |  |
| Poaceae | *Sporobolus* | *Sporobolus fimbriatus* | rbcL | KNPA248-09.rbcLa | KNPA276-09.rbcLa | KNPA336-09.rbcLa |
| Poaceae | *Sporobolus* | *Sporobolus ioclados* | trnL | KR737837 | KR737893 | KR738462 |
| Poaceae | *Sporobolus* | *Sporobolus ioclados* | rbcL | KR737293 | KR737149 | KR736630 |
| Poaceae | *Sporobolus* | *Sporobolus nitens* | rbcL | KNPA203-09.rbcLa | KNPA213-09.rbcLa | KNPA392-09.rbcLa |
| Poaceae | *Sporobolus* | *Sporobolus pyramidalis* | trnL | DQ655902 | KR738244 |  |
| Poaceae | *Sporobolus* | *Sporobolus pyramidalis* | rbcL | LN908017 | MF998396 | MH464348 |
| Poaceae | *Sporobolus* | *Sporobolus stapfianus* | trnL | KR738190 | KR738279 | KR737677 |
| Poaceae | *Sporobolus* | *Sporobolus stapfianus* | rbcL | KR736593 | KR737009 | KR737103 |
| Poaceae | *Stipagrostis* | *Stipagrostis hirtigluma* | trnL | GQ924330 | GQ924333 | GQ924331 |
| Poaceae | *Themeda* | *Themeda triandra* | trnL | KY991343 | KY991323 | KY991333 |
| Poaceae | *Themeda* | *Themeda triandra* | rbcL | LN908022 | KF163556 | KF163557 |
| Poaceae | *Trachypogon* | *Trachypogon spicatus* | rbcL | EF125123 | LN908023 | MG772773 |
| Poaceae | *Tragus* | *Tragus berteronianus* | trnL | DQ655865 | KR737710 | KR738371 |
| Poaceae | *Tragus* | *Tragus berteronianus* | rbcL | MF998388 | MN185264 | KR736801 |
| Poaceae | *Tricholaena* | *Tricholaena monachne* | trnL | GU594574 |  |  |
| Poaceae | *Tricholaena* | *Tricholaena monachne* | rbcL | MF998184 | HE577881 |  |
| Poaceae | *Trichoneura* | *Trichoneura grandiglumis* | rbcL | HE575835 | KC123388 | FN870947 |
| Poaceae | *Tripogon* | *Tripogon minimus* | rbcL | AM849342 |  |  |
| Poaceae | *Urochloa* | *Urochloa brachyura* | trnL | KR737656 | KR738144 | KR738185 |
| Poaceae | *Urochloa* | *Urochloa brachyura* | rbcL | MF998166 | KR736435 | KR737004 |
| Poaceae | *Urochloa* | *Urochloa mosambicensis* | trnL | GU594532 | PNG431-18.trnL-F |  |
| Poaceae | *Urochloa* | *Urochloa mosambicensis* | rbcL | MF998168 | KNPA247-09.rbcLa | KNPA278-09.rbcLa |
| Poaceae | *Urochloa* | *Urochloa panicoides* | rbcL | LT608220 | MF998167 | HE573318 |
| Polygalaceae | *Polygala* | *Polygala erioptera* | trnL | AF366990 | GQ889131 | AM234331 |
| Polygalaceae | *Polygala* | *Polygala erioptera* | rbcL | AM234219 | KU551064 |  |
| Polygalaceae | *Polygala* | *Polygala sphenoptera* | trnL | KR737872 | KR738419 | KR738325 |
| Polygalaceae | *Polygala* | *Polygala sphenoptera* | rbcL | KR736665 | KR737150 | KR737085 |
| Polygalaceae | *Polygala* | *Polygala uncinata* | trnL | GQ889215 | AM234318 | GQ888867 |
| Polygalaceae | *Polygala* | *Polygala uncinata* | rbcL | AM234192 |  |  |
| Polygonaceae | *Oxygonum* | *Oxygonum dregeanum* | trnL | JN161140 |  |  |
| Polygonaceae | *Oxygonum* | *Oxygonum sinuatum* | trnL | KR537752 | KR738018 | KR737905 |
| Polygonaceae | *Oxygonum* | *Oxygonum sinuatum* | rbcL | KR736460 | KR736970 | KR736745 |
| Polygonaceae | *Persicaria* | *Persicaria decipiens* | trnL | KR737613 | KR738471 | KR738613 |
| Polygonaceae | *Persicaria* | *Persicaria decipiens* | rbcL | KR737461 | KR736496 | KR737303 |
| Portulacaceae | *Portulaca* | *Portulaca oleracea* | rbcL | MG946858 | HQ621340 | AY875249 |
| Portulacaceae | *Portulaca* | *Portulaca quadrifida* | trnL | MK261720 | MK261577 | KR738472 |
| Portulacaceae | *Portulaca* | *Portulaca quadrifida* | rbcL | KR737010 | KR737304 | KR736957 |
| Pteridaceae | *Cheilanthes* | *Cheilanthes viridis* | trnL | GU935603 | GU935601 |  |
| Pteridaceae | *Pellaea* | *Pellaea calomelanos* | trnL | KR737669 | KR737688 | KR737675 |
| Pteridaceae | *Pellaea* | *Pellaea calomelanos* | rbcL | KR736447 | KR736465 | KR736453 |
| Rhamnaceae | *Berchemia* | *Berchemia discolor* | rbcL | KNPA181-08.rbcLa | JF265302 | JX572329 |
| Rhamnaceae | *Berchemia* | *Berchemia zeyheri* | rbcL | JF265303 | JX572330 |  |
| Rhamnaceae | *Ziziphus* | *Ziziphus mucronata* | trnL | KR083151 | MK261551 |  |
| Rhamnaceae | *Ziziphus* | *Ziziphus mucronata* | rbcL | JF265666 | JX573107 |  |
| Rubiaceae | Agathisanthemum | *Agathisanthemum bojeri* | trnL | EU543077 |  |  |
| Rubiaceae | Agathisanthemum | *Agathisanthemum bojeri* | rbcL | Z68787 |  |  |
| Rubiaceae | *Breonadia* | *Breonadia salicina* | rbcL | JF265313 | AJ346967 | JX572348 |
| Rubiaceae | *Canthium* | *Canthium armatum* | trnL | AJ620155 | JQ958229 |  |
| Rubiaceae | *Canthium* | *Canthium armatum* | rbcL | AM117259 | JF265553 | JX572859 |
| Rubiaceae | *Canthium* | *Canthium ciliatum* | trnL | JQ958230 | AJ620121 |  |
| Rubiaceae | *Canthium* | *Canthium ciliatum* | rbcL | JX572365 |  |  |
| Rubiaceae | *Catunaregam* | *Catunaregam spinosa* | rbcL | JF265332 | KNPA153-08.rbcLa | KX910827 |
| Rubiaceae | *Conostomium* | *Conostomium natalense* | trnL | EU543085 |  |  |
| Rubiaceae | *Conostomium* | *Conostomium natalense* | rbcL | EU542927 |  |  |
| Rubiaceae | *Gardenia* | *Gardenia volkensii* | rbcL | JF265446 | JX572624 |  |
| Rubiaceae | *Hyperacanthus* | *Hyperacanthus amoenus* | trnL | KF965209 |  |  |
| Rubiaceae | *Hyperacanthus* | *Hyperacanthus amoenus* | rbcL | KF964840 | JF265481 | JX572687 |
| Rubiaceae | *Kohautia* | *Kohautia amatymbica* | trnL | EU543117 | GU951652 |  |
| Rubiaceae | *Kohautia* | *Kohautia caespitosa* | trnL | EU543118 | GU951659 |  |
| Rubiaceae | *Kohautia* | *Kohautia caespitosa* | rbcL | GU951593 | GU951592 | FJ695364 |
| Rubiaceae | *Kohautia* | *Kohautia cynanchica* | trnL | EU543120 |  |  |
| Rubiaceae | *Kohautia* | *Kohautia cynanchica* | rbcL | EU542965 |  |  |
| Rubiaceae | *Kohautia* | *Kohautia virgata* | trnL | EU543124 |  |  |
| Rubiaceae | *Kohautia* | *Kohautia virgata* | rbcL | EU542969 |  |  |
| Rubiaceae | *Kraussia* | *Kraussia floribunda* | trnL | KF965216 | AM117368 | KT218924 |
| Rubiaceae | *Kraussia* | *Kraussia floribunda* | rbcL | KF964848 |  |  |
| Rubiaceae | *Pavetta* | *Pavetta catophylla* | rbcL | JF265542 | JX572837 | KNPA160-08.rbcLa |
| Rubiaceae | *Pavetta* | *Pavetta lanceolata* | trnL | AM117377 |  |  |
| Rubiaceae | *Pavetta* | *Pavetta lanceolata* | rbcL | JF265544 | JX572841 |  |
| Rubiaceae | *Pavetta* | *Pavetta schumanniana* | trnL | KM592135 | JN053647 | JN053646 |
| Rubiaceae | *Pavetta* | *Pavetta schumanniana* | rbcL | JX572843 | JF265545 | KNPA106-08.rbcLa |
| Rubiaceae | *Pentodon* | *Pentodon pentandrus* | trnL | EU543154 |  |  |
| Rubiaceae | *Psydrax* | *Psydrax obovata* | trnL | AJ620161 | JQ958217 |  |
| Rubiaceae | *Psydrax* | *Psydrax obovata* | rbcL | HM164176 | JX572899 |  |
| Rubiaceae | *Pyrostria* | *Pyrostria hystrix* | trnL | AJ620168 | JQ958226 |  |
| Rubiaceae | *Pyrostria* | *Pyrostria hystrix* | rbcL | AM117262 | JF265568 | JX572915 |
| Rubiaceae | *Richardia* | *Richardia brasiliensis* | rbcL | KJ773839 | KY626893 |  |
| Rubiaceae | *Tricalysia* | *Tricalysia junodii* | trnL | KT218952 |  |  |
| Rubiaceae | *Tricalysia* | *Tricalysia junodii* | rbcL | JF265633 | JX573060 | KNPA169-08.rbcLa |
| Rubiaceae | *Vangueria* | *Vangueria infausta* | trnL | JQ958289 | KF488321 |  |
| Rubiaceae | *Vangueria* | *Vangueria infausta* | rbcL | DQ131794 | JF265644 | JX573076 |
| Rutaceae | *Clausena* | *Clausena anisata* | trnL | MK261535 |  |  |
| Rutaceae | *Clausena* | *Clausena anisata* | rbcL | MK285240 |  |  |
| Rutaceae | *Ptaeroxylon* | *Ptaeroxylon obliquum* | rbcL | FR747838 | JF265562 |  |
| Rutaceae | *Vepris* | *Vepris carringtoniana* | rbcL | KU567994 |  |  |
| Rutaceae | *Zanthoxylum* | *Zanthoxylum humile* | rbcL | JX573103 | JF265664 |  |
| Salicaceae | *Dovyalis* | *Dovyalis caffra* | rbcL | JF265397 | JQ412354 | JX572533 |
| Salicaceae | *Flacourtia* | *Flacourtia indica* | rbcL | MH332467 | MH549859 | GU135218 |
| Salicaceae | *Scolopia* | *Scolopia zeyheri* | rbcL | MT231417 | JF265587 |  |
| Sapindaceae | *Allophylus* | *Allophylus decipiens* | trnL | KX584992 |  |  |
| Sapindaceae | *Allophylus* | *Allophylus decipiens* | rbcL | JF265283 |  |  |
| Sapindaceae | *Cardiospermum* | *Cardiospermum corindum* | trnL | KX585007 |  |  |
| Sapindaceae | *Cardiospermum* | *Cardiospermum halicacabum* | trnL | AY594504 | JN681469 | MK187002 |
| Sapindaceae | *Cardiospermum* | *Cardiospermum halicacabum* | rbcL | GU935448 | KJ773336 | MK348962 |
| Sapotaceae | *Manilkara* | *Manilkara mochisia* | rbcL | JF265514 | KNPA122-08.rbcLa | PNG408-18.rbcL |
| Scrophulariaceae | *Aptosimum* | *Aptosimum lineare* | trnL | MZ461548 |  |  |
| Scrophulariaceae | *Aptosimum* | *Aptosimum lineare* | rbcL | MZ461572 |  |  |
| Solanaceae | *Datura* | *Datura stramonium* | trnL | MK261363 |  |  |
| Solanaceae | *Datura* | *Datura stramonium* | rbcL | MK285235 |  |  |
| Solanaceae | *Solanum* | *Solanum aculeastrum* | trnL | DQ812102 | HQ721896 | HM016460 |
| Solanaceae | *Solanum* | *Solanum aculeastrum* | rbcL | JQ025091 |  |  |
| Solanaceae | *Solanum* | *Solanum catombelense* | trnL | HQ721909 |  |  |
| Solanaceae | *Solanum* | *Solanum catombelense* | rbcL | JF265599 |  |  |
| Solanaceae | *Solanum* | *Solanum incanum* | trnL | EU176144 | GQ163522 | HQ721924 |
| Solanaceae | *Solanum* | *Solanum lichtensteinii* | trnL | EU176146 | EU176147 | KU719798 |
| Solanaceae | *Solanum* | *Solanum lichtensteinii* | rbcL | JF265600 | SLNM001-19.rbcL | SLNM021-19.rbcL |
| Solanaceae | *Solanum* | *Solanum nigrum* | trnL | MK261424 | MK261691 | MK261711 |
| Solanaceae | *Solanum* | *Solanum nigrum* | rbcL | MK285279 | MK285277 | MK285280 |
| Solanaceae | *Solanum* | *Solanum panduriforme* | trnL | EU176143 | MK187217 | MK187218 |
| Solanaceae | *Solanum* | *Solanum panduriforme* | rbcL | EU213513 | EU213514 | EU213515 |
| Solanaceae | *Solanum* | *Solanum seaforthianum* | rbcL | MH722380 | SLNM016-19.rbcL |  |
| Solanaceae | *Solanum* | *Solanum tettense* | trnL | HQ721949 |  |  |
| Solanaceae | *Withania* | *Withania somnifera* | trnL | EU581069 | KR737662 | KR738232 |
| Solanaceae | *Withania* | *Withania somnifera* | rbcL | MG946905 | FJ914179 | KY656708 |
| Stilbaceae | *Nuxia* | *Nuxia oppositifolia* | trnL | AJ621117 |  |  |
| Stilbaceae | *Nuxia* | *Nuxia oppositifolia* | rbcL | JF265527 | JX572793 |  |
| Talinaceae | *Talinum* | *Talinum caffrum* | trnL | KR738610 | KR738655 |  |
| Talinaceae | *Talinum* | *Talinum caffrum* | rbcL | KR736800 | KR737509 | KR736715 |
| Talinaceae | *Talinum* | *Talinum portulacifolium* | trnL | KM261958 | MK261382 |  |
| Urticaceae | *Pouzolzia* | *Pouzolzia mixta* | rbcL | JQ025073 |  |  |
| Vahliaceae | *Vahlia* | *Vahlia capensis* | trnL | HQ412984 | MK187240 |  |
| Vataceae | *Rhoicissus* | *Rhoicissus revoilii* | trnL | JQ182555 |  |  |
| Vataceae | *Rhoicissus* | *Rhoicissus revoilii* | rbcL | JQ182456 | JX572926 | JF265572 |
| Vataceae | *Rhoicissus* | *Rhoicissus tridentata* | trnL | JF437341 |  |  |
| Vataceae | *Rhoicissus* | *Rhoicissus tridentata* | rbcL | JQ182443 | JF265574 | JQ025083 |
| Velloziaceae | *Xerophyta* | *Xerophyta retinervis* | trnL | JN016936 | JX287056 |  |
| Velloziaceae | *Xerophyta* | *Xerophyta retinervis* | rbcL | KT204817 | JX032732 | EU213530 |
| Verbenaceae | *Chascanum* | *Chascanum pinnatifidum* | rbcL | KBGPP004-18.rbcLa |  |  |
| Verbenaceae | *Lantana* | *Lantana camara* | trnL | HM216633 | AF231884 |  |
| Verbenaceae | *Lantana* | *Lantana camara* | rbcL | AF156736 | HM850104 |  |
| Verbenaceae | *Lantana* | *Lantana rugosa* | trnL | HM216637 |  |  |
| Verbenaceae | *Lantana* | *Lantana rugosa* | rbcL | JF265500 | JX572712 | KBGPP079-18.rbcLa |
| Verbenaceae | *Lippia* | *Lippia javanica* | trnL | HM216644 | KR737647 | KR737720 |
| Verbenaceae | *Lippia* | *Lippia javanica* | rbcL | KR736512 | JF265503 | JQ025061 |
| Verbenaceae | *Verbena* | *Verbena bonariensis* | trnL | EF571518 | KR738057 | KR738492 |
| Verbenaceae | *Verbena* | *Verbena bonariensis* | rbcL | HM850443 | KF724243 | KR737327 |
| Violaceae | *Hybanthus* | *Hybanthus enneaspermus* | trnL | MK261549 | MK261582 |  |
| Violaceae | *Hybanthus* | *Hybanthus enneaspermus* | rbcL | AB354418 | KC699584 | GENG1060-15.rbcL |
| Vitaceae | *Cissus* | *Cissus cornifolia* | trnL | JF437308 | JX476869 | JX476870 |
| Vitaceae | *Cissus* | *Cissus cornifolia* | rbcL | JX476449 | KNPA583-09.rbcLa | JF265339 |
| Vitaceae | *Cissus* | *Cissus quadrangularis* | trnL | JX476898 | JF437313 | JX313444 |
| Vitaceae | *Cissus* | *Cissus quadrangularis* | trnL | AJ419720 | KX951084 | MN125636 |
| Vitaceae | *Cissus* | *Cissus rotundifolia* | trnL | JF437314 | MK187017 | KR737718 |
| Vitaceae | *Cissus* | *Cissus rotundifolia* | rbcL | MF349686 | GENG1713-16.rbcL | PNG311-18.rbcL |
| Vitaceae | *Cyphostemma* | *Cyphostemma cirrhosum* | trnL | MH086946 |  |  |
| Vitaceae | *Cyphostemma* | *Cyphostemma cirrhosum* | rbcL | MH086886 | MH086835 |  |
| Vitaceae | *Cyphostemma* | *Cyphostemma schlechteri* | trnL | MH086936 |  |  |
| Vitaceae | *Cyphostemma* | *Cyphostemma schlechteri* | rbcL | MH086876 | MH086826 |  |
| Ximeniaceae | *Ximenia* | *Ximenia americana* | rbcL | GQ997898 | MH390668 | DQ790149 |
| Ximeniaceae | *Ximenia* | *Ximenia caffra* | rbcL | MN166693 | MN216453 | MN216487 |
| Zygophyllaceae | *Balanites* | *Balanites maughamii* | rbcL | JX572324 |  |  |
| Zygophyllaceae | *Tribulus* | *Tribulus terrestris* | trnL | MN587215 | KP087733 | KT377266 |
| Zygophyllaceae | *Tribulus* | *Tribulus terrestris* | rbcL | MG946874 | AM235167 | MN525779 |
