# Supplementary material for: Introducing an rbcL and a trnL reference library to aid in the metabarcoding analysis of foraged plants from two semi-arid eastern South African savanna bioregions
Source: PLoS One. 2023 May 19;18(5):e0286144. doi: 10.1371/journal.pone.0286144 (PMC10198553; doi:10.1371/journal.pone.0286144)
Supplement: S3 Table — (DOCX) [file pone.0286144.s003.docx]

Supplementary Table 3:. List of species, for which *rbc*L and/or *trn*L barcode sequences were not available in public databases, that were sequenced at the NWU with the *rbc*L and *trn*L barcode, submitted to GenBank, and included in the *rbc*L and *trn*L reference datasets respectively.

| Barcode | Family | Genus | Species | GenBank accession number |
| --- | --- | --- | --- | --- |
| rbcL (TOTAL: 24) | Malvaceae | *Abutilon* | *Abutilon austroafricanum* | MZ461571 |
|  | Scrophulariaceae | *Aptosimum* | *Aptosimum lineare* | MZ461572 |
|  | Acanthaceae | *Barleria* | *Barleria elegans* | MZ461573 |
|  | Acanthaceae | *Barleria* | *Barleria prionitis* | MZ461574 |
|  | Acanthaceae | *Blepharis* | *Blepharis innocua* | MZ461575 |
|  | Acanthaceae | *Blepharis* | *Blepharis integrifolia* | MZ461576 |
|  | Fabaceae | *Chamaecrista* | *Chamaecrista mimosoides* | MZ461577 |
|  | Combretaceae | *Combretum* | *Combretum zeyheri* | MZ461578 |
|  | Acanthaceae | *Crabbea* | *Crabbea velutina* | MZ461579 |
|  | Acanthaceae | *Ecbolium* | *Ecbolium glabratum* | MZ461580 |
|  | Phyllanthaceae | *Flueggea* | *Flueggea virosa* | MZ461581 |
|  | Malvaceae | *Grewia* | *Grewia bicolor* | MZ461582 |
|  | Malvaceae | *Hibiscus* | *Hibiscus sabiensis* | MZ461583 |
|  | Acanthaceae | *Justicia* | *Justicia flava* | MZ461584 |
|  | Acanthaceae | *Justicia* | *Justicia protracta* | MZ461585 |
|  | Lamiaceae | *Leucas* | *Leucas glabrata* | MZ461586 |
|  | Malvaceae | *Melhania* | *Melhania prostrata* | MZ461587 |
|  | Phyllanthaceae | *Phyllanthus* | *Phyllanthus asperulatus* | MZ461588 |
|  | Phyllanthaceae | *Phyllanthus* | *Phyllanthus incurvus* | MZ461589 |
|  | Portulacaceae | *Portulaca* | *Portulaca quadrifida* | MZ461590 |
|  | Bignoniaceae | *Rhigozum* | *Rhigozum zambesiacum* | MZ461591 |
|  | Fabaceae | *Rhynchosia* | *Rhynchosia fleckii* | MZ461592 |
|  | Solanaceae | *Solanum* | *Solanum delagoense* | MZ461593 |
|  | Euphorbiaceae | *Tragia* | *Tragia dioica* | MZ461594 |
| trnL (TOTAL: 24) | Malvaceae | *Abutilon* | *Abutilon austroafricanum* | MZ461547 |
|  | Scrophulariaceae | *Aptosimum* | *Aptosimum lineare* | MZ461548 |
|  | Acanthaceae | *Barleria* | *Barleria elegans* | MZ461549 |
|  | Acanthaceae | *Barleria* | *Barleria prionitis* | MZ461550 |
|  | Acanthaceae | *Blepharis* | *Blepharis innocua* | MZ461551 |
|  | Pedaliaceae | *Ceratotheca* | *Ceratotheca triloba* | MZ461552 |
|  | Combretaceae | *Combretum* | *Combretum zeyheri* | MZ461553 |
|  | Burseraceae | *Commiphora* | *Commiphora africana* | MZ461554 |
|  | Acanthaceae | *Crabbea* | *Crabbea velutina* | MZ461555 |
|  | Acanthaceae | *Ecbolium* | *Ecbolium glabratum* | MZ461556 |
|  | Celastraceae | *Elaeodendron* | *Elaeodendron transvaalense* | MZ461557 |
|  | Convolvulaceae | *Evolvulus* | *Evolvulus alsinoides* | MZ461558 |
|  | Asteraceae | *Geigeria* | *Geigeria burkei* | MZ461559 |
|  | Fabaceae | *Indigofera* | *Indigofera tinctoria* | MZ461560 |
|  | Convolvulaceae | *Ipomoea* | *Ipomoea crassipes* | MZ461561 |
|  | Acanthaceae | *Justicia* | *Justicia flava* | MZ461562 |
|  | Acanthaceae | *Justicia* | *Justicia protracta* | MZ461563 |
|  | Malvaceae | *Melhania* | *Melhania prostrata* | MZ461564 |
|  | Convolvulaceae | *Xenostegia* | *Xenostegia tridentata* | MZ461565 |
|  | Phyllanthaceae | *Phyllanthus* | *Phyllanthus asperulatus* | MZ461566 |
|  | Phyllanthaceae | *Phyllanthus* | *Phyllanthus incurvus* | MZ461567 |
|  | Fabaceae | *Rhynchosia* | *Rhynchosia fleckii* | MZ461568 |
|  | Convolvulaceae | *Seddera* | *Seddera capensis* | MZ461569 |
|  | Malvaceae | *Triumfetta* | *Triumfetta pentandra* | MZ461570 |
