# Supplementary material for: Introducing an rbcL and a trnL reference library to aid in the metabarcoding analysis of foraged plants from two semi-arid eastern South African savanna bioregions
Source: PLoS One. 2023 May 19;18(5):e0286144. doi: 10.1371/journal.pone.0286144 (PMC10198553; doi:10.1371/journal.pone.0286144)
Supplement: S1 File — (DOCX) [file pone.0286144.s004.docx]

Links to shapefiles are used to create the map in Figure 1.

**[56]. SANBI Vegetation Map**: <https://bgis.sanbi.org/SpatialDataset/Detail/18>

**[57]. Municipal Demarcation Board**: <https://dataportal-mdb-sa.opendata.arcgis.com/datasets/37d790cf4f3b4b0ebd0ac501eae9b17d_0/explore?location=-29.911228%2C26.005192%2C5.96>

**[58]. The National Geographic Society**: <https://services.arcgisonline.com/arcgis/rest/services/NatGeo_World_Map/MapServer>
